# Rationalization of the Color Properties of Fluorescein in the Solid State: A Combined Computational and Experimental Study

**DOI:** 10.1002/chem.201601340

**Published:** 2016-06-15

**Authors:** Mihails Arhangelskis, Mark D. Eddleston, David G. Reid, Graeme M. Day, Dejan‐Krešimir Bučar, Andrew J. Morris, William Jones

**Affiliations:** ^1^ Department of ChemistryUniversity of CambridgeLensfield RoadCambridgeCB2 1EWUK; ^2^ School of ChemistryUniversity of Southampton, HighfieldSouthamptonSO17 1BJUK; ^3^ Department of ChemistryUniversity College London20 Gordon StreetLondonWC1H 0AJUK; ^4^ Theory of Condensed Matter GroupCavendish LaboratoryUniversity of CambridgeJ J Thomson AvenueCambridgeCB3 0HEUK

**Keywords:** density functional calculations, fluorescein, optical spectroscopy, surface analysis, tautomerism

## Abstract

Fluorescein is known to exist in three tautomeric forms defined as quinoid, zwitterionic, and lactoid. In the solid state, the quinoid and zwitterionic forms give rise to red and yellow materials, respectively. The lactoid form has not been crystallized pure, although its cocrystal and solvate forms exhibit colors ranging from yellow to green. An explanation for the observed colors of the crystals is found using a combination of UV/Vis spectroscopy and plane‐wave DFT calculations. The role of cocrystal coformers in modifying crystal color is also established. Several new crystal structures are determined using a combination of X‐ray and electron diffraction, solid‐state NMR spectroscopy, and crystal structure prediction (CSP). The protocol presented herein may be used to predict color properties of materials prior to their synthesis.

## Introduction

Organic pigments are of great importance to society. They are used in the production of photovoltaic materials,^1^ optical data storage devices,^2^ and also in the coloration of plastics^3^ and in the textile industry. With the current rapid population growth there is an ever‐increasing demand for pigments tailored for very specific applications.^4^ The ability to design new pigment materials with desirable properties, however, requires a deep theoretical understanding of the underlying physical processes that involve the interaction of light with crystalline materials. The development of computational methods, particularly plane‐wave electronic structure calculations,^5^ coupled with constantly increasing available computer power have allowed researchers to study solid‐state phenomena responsible for a variety of applications, particularly in the areas of semiconducting materials,^6^, ^7^ organic photovoltaics,^8^ solid electrolytes,^9^ lithium‐ion batteries,^10^ magnetic materials,^11^ surface chemistry, and catalysis.^12^ The situation with pigments, however, is different: many commercially important materials are used without any detailed understanding of the structural features responsible for their optical properties. Determination of the crystal structure of a pigment would be the first step in understanding the behavior of the material; once the structure is known, band structure calculations can be performed to shed light on the optical properties of the material. Despite the recent advancements in the methods of crystal structure determination from powder X‐ray diffraction (PXRD) and crystal structure prediction (CSP),^13^ the structures of many industrially important pigments remain unknown or are determined decades after the beginning of industrial production.^14^, ^15^ The lack of computational studies makes the development of new pigment materials largely a trial and error process, a trend that must be changed to fulfil society's needs for new advanced optical materials.

Current developments in periodic DFT band structure calculations have enabled the modeling of optical absorption and reflection spectra, as well as establishing the electronic density of states (DOS) of materials.^16^ The ability to perform such calculations not only provides the opportunity to understand the optical properties of existing pigments (and other materials), but also to predict optical properties of new materials before being prepared.

We describe herein how recent developments in both experimental and computational methods can be utilized to characterize the optical solid‐state properties of the model pigment fluorescein. The study serves as an example of how computational studies supplement experiments in providing the most detailed understanding of the complex optical solid‐state properties of pigments, as well as providing a general strategy for the computational development of new organic pigments and other materials.

The optical properties of organic pigments depend not only on the molecular structure of the compound but also on the associated crystal packing: different polymorphs of the same compound often show considerable variation in color as a result of differences in molecular conformation or intermolecular interactions.^17^, ^18^ An archetypal example of such a system is the compound ROY, which produces a wide variety of polymorphs with colors ranging from red to orange and yellow.^18^, ^19^ Even greater diversity may be achieved by producing multicomponent cocrystals in which coformer molecules electronically interact with the organic chromophore,^20^ often causing large changes in the band gap and, therefore, color.^21^ Furthermore, the use of cocrystals in optical materials is not limited to the modification of color: recent work in our group has shown that cocrystallization is also a successful method for modifying the luminescent properties of organic compounds.^22^, ^23^

During studies of the solid‐state behavior of organic chromophores, our attention was drawn to the pigment fluorescein. This compound is most widely used in the water‐soluble disodium‐salt form. Therefore, the solid‐state behavior of the neutral compound has not received equivalent attention and the available information is often ambiguous and incomplete.^24^, ^25^ Nonetheless, the behavior of crystalline fluorescein represents a curious case of an interplay of molecular tautomerism^26^ and the effects of crystal packing.

**Figure 1:**
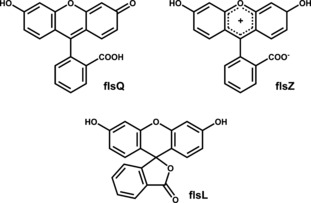
Tautomeric forms of fluorescein: quinoid (**flsQ**), zwitterionic (**flsZ**), and lactoid (**flsL**).

**Figure 2:**
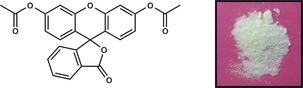
Structure and appearance of the crystalline lactoid form of diacetylfluorescein.

**Figure 3:**
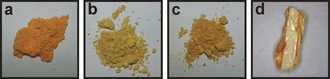
Photographs of selected samples of fluorescein a) **flsZ**, b) **flsL**:dioxane hemisolvate powder, c) **flsL**:dioxane hemipentasolvate powder, and d) single crystal of **flsL**:dioxane hemipentasolvate. It can be seen that the color of the single crystal is paler then that of the powder lactoid samples.

The fluorescein (**fls**) molecule exists in three tautomeric forms (Figure 1): the quinoid form (**flsQ**), the zwitterionic form (**flsZ**), and the lactoid form (**flsL**). In the solid state, **flsQ** is reported to form a red powder,^24^ for which the crystal structure has been determined using PXRD.^27^ The **flsZ** tautomer, on the other hand, produces a yellow solid, for which the crystal structure has not been reported. The third tautomer, **flsL**, has not been crystallized in pure form, although the crystals of this hypothetical solid are expected to be colorless by analogy to a related lactoid compound diacetylfluorescein (Figure 2).^28^ Despite the inability to obtain crystals of pure **flsL**, several structures containing the lactoid tautomer in the form of acetone,^29^ methanol,^30^ and 1,4‐dioxane^25^ solvates have been produced. Descriptions of the colors of these solvates in the literature, however, are rather ambiguous as different authors characterize them as colorless,^24^ amber‐yellow,^29^ or even orange.^30^ It was also suggested^30^ that the yellow coloration of the **flsL** solvate crystals may be caused by partial loss of solvent molecules from the crystal surface. The resulting “free” **flsL** molecules then convert into the yellow **flsZ** form, thus generating a colored layer on the surface of the crystals. Our own attempts to produce the solvates of fluorescein have shown that the color of these materials depends on the particle size: powdered materials display significantly brighter colors than larger crystals (Figure 3), suggesting that the color is indeed formed within a surface layer. Herein we present a combined experimental and computational investigation that confirms the existence of the zwitterion surface layer and offers possible reasons for its formation.

Recently, our group has reported three new cocrystals of **flsL**.^31^ These cocrystals were prepared using mechanochemical methods and displayed colors ranging from yellow to green. It was considered instructive to establish whether the mechanism of color generation in the cocrystals of **flsL** is the same as in the solvates (namely, through the formation of the **flsZ** surface layer), or whether the presence of another molecule (i.e. cocrystal former) in the crystal lattice plays an active role in the optical properties of the resulting multicomponent form.

In this study, we use a variety of experimental and theoretical techniques to obtain a complete understanding of the optical behavior of fluorescein in the solid state. The previously unreported crystal structures of pure **flsZ** along with dioxane solvates of **flsL** are determined using a combination of X‐ray diffraction, electron diffraction,^32^, ^33^, ^34^, ^35^ and CSP. The identity of the three tautomeric forms of fluorescein is further established through ^13^C solid‐state NMR spectroscopy and calculations of NMR chemical shielding. Finally, the existence of the zwitterion surface layer on the **flsL**‐containing crystals is confirmed by UV/Vis spectroscopy, and insight into the mechanism of its formation is given. Additionally, the role of the coformers in the optical properties of fluorescein cocrystals is established with the aid of band structure calculations. Most importantly, however, this study will demonstrate how the synergy of (standard and state‐of‐the‐art) experimental and computational techniques can be used to obtain the best understanding of the properties of pigment materials. We believe that the protocol demonstrated herein not only provides a better understanding of color generation in pigments, but also demonstrates that it is possible to engineer new materials with desirable properties.

## Results and Discussion

To perform the calculations of optical properties of materials it is first necessary to determine the corresponding crystal structures. The structures of diacetylfluorescein,^28^ **flsQ**,^27^ **flsL** acetone solvate,^29^ and cocrystals with acridine, phenanthridine, and pyrazine^31^ have been previously reported. The preparation of unsolvated **flsZ** and **flsL**:dioxane hemisolvate have been reported without structure determination,^24^ while the **flsL**:dioxane hemipentasolvate has not previously been reported.

### Crystal structure determination of new fls crystal forms

#### flsL:dioxane hemipentasolvate

**Figure 4:**
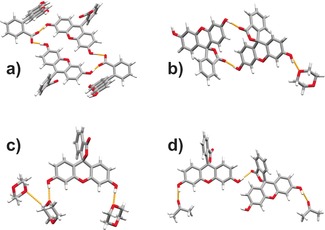
Crystal structures of a) pure **flsZ**, b) **flsL**:dioxane hemisolvate, c) **flsL**:dioxane hemipentasolvate and d) **flsL**:acetone monosolvate Form II.

The **flsL**:dioxane hemipentasolvate was crystallized from solution and the structure was determined using single‐crystal X‐ray diffraction. This material crystallizes in the monoclinic space group *P*2_1_/*n* having one molecule of **flsL** and 2.5 molecules of 1,4‐dioxane in the asymmetric unit. The potential hydrogen bonding groups of dioxane molecules in this crystal structure are rather poorly utilized: two of the dioxane molecules each use one of their oxygen atoms to form O−H⋅⋅⋅O hydrogen bonds with hydroxy groups of **flsL**. The third dioxane molecule, which is located on an inversion center, only interacts through weak C−H⋅⋅⋅O interactions with the other dioxane molecules (Figure 4 c). It is, therefore, not surprising that the fluorescein dioxane hemipentasolvate converts to the dioxane hemisolvate within several days, even at room temperature.

#### flsL:dioxane hemisolvate

The **flsL**:dioxane hemisolvate was obtained by heating the hemipentasolvate at 80 °C for 15 min. The desolvation process is accompanied by significant structural rearrangements that cause the crystals to shatter. As a consequence, structure determination had to be performed using powder X‐ray diffraction (for details, see Section 4 in the Supporting Information).

The **flsL**:dioxane hemisolvate crystallizes in the triclinic unit cell, space group *P* $\bar{1}$ . The asymmetric unit contains one **flsL** molecule and half of a dioxane molecule located on an inversion center. Both oxygen atoms of the dioxane molecule are hydrogen‐bonded to **flsL** through O−H⋅⋅⋅O interactions. Furthermore, fluorescein molecules form centrosymmetric dimers through O−H⋅⋅⋅O(carbonyl) interactions, which were not present in the hemipentasolvate structure (Figure 4 b). Overall, the hemisolvate structure offers a more even balance between the number of hydrogen‐bond donors and acceptors, which is reflected in a very high temperature for complete desolvation (150 °C).

#### flsL:acetone monosolvate form II

The **flsL**:acetone monosolvate was obtained by evaporating an acetone solution of fluorescein. While initial crystallization experiments resulted in the formation of the previously reported solvate structure (Form I),^29^ later attempts to reproduce the material mostly resulted in the formation of a new polymorph of the solvate (Form II).

The crystal structure of Form II was determined from X‐ray powder data. The material crystallizes in the monoclinic *P*2_1_/*c* space group with one **flsL** and one acetone molecule in the asymmetric unit. The principal intermolecular interactions are the O−H⋅⋅⋅O(acetone) and O−H⋅⋅⋅O(**flsL**, carbonyl) hydrogen bonds (Figure 4 d). Careful inspection of the structure revealed that the new solvate polymorph is isostructural with the **flsL**:pyrazine cocrystal, whereby the acetone molecules are replacing pyrazine in the cocrystal structure (Supporting Information, Figure S19).

Making a slurry of Form II resulted in the conversion to Form I, suggesting that Form I is the thermodynamically stable polymorph. This was further supported by the solid‐state DFT (PBE+G06) energy calculations, which showed that the lattice energy of Form II is 11.2 kJ mol^−1^ higher than that of Form I.

#### Crystal structure of flsZ

The zwitterionic form of fluorescein (**flsZ**) was crystallized by rapidly quenching an aqueous alkaline solution of fluorescein disodium salt with acetic acid. As a result of such rapid crystallization the product was obtained as a very fine powder. The small particle size of the material resulted in broad peaks in the X‐ray powder pattern, which made direct structure determination from powder data difficult. To elucidate the crystal structure we have studied the crystal energy landscape of possible **flsZ** structures using the crystal structure prediction (CSP) methods described previously.^36^, ^37^, ^38^

The CSP calculations^39^ generate a set of trial crystal structures that a given molecule can form; these are lattice energy minimized and ranked by their lattice energy.^40^ Analysis of the predicted structures provides information about the most prevalent intermolecular interactions and supramolecular synthons^41^ responsible for the formation of the crystal. The experimental structure may then be determined by validating the predicted structure against experimental data, such as X‐ray powder patterns,^37^, ^39^ TEM electron diffraction data,^33^, ^34^ or solid‐state NMR^44^, ^45^ spectra.

X‐ray powder patterns were calculated using the software package *CCDC Mercury* 46 for each of the low energy (within 15 kJ mol^−1^ of the global minimum) predicted crystal structures and the assignment of the experimental structure was performed by comparing the calculated and experimental patterns (for X‐ray powder pattern comparisons, see the Supporting Information, Figures S31–S34). This analysis revealed the structure ranked third, 6.6 kJ mol^−1^ above the global minimum in lattice energy, as the most likely candidate for the observed crystal structure. This structural assignment was subsequently validated by successful Rietveld refinement^47^ of the predicted structure against the experimental pattern. While the PXRD analysis demonstrates that predicted structure number 3 is the major crystalline component of yellow **fls**, the diffraction pattern also indicates that an amorphous component may be present in the bulk material (Supporting Information, Figure S8).

**Figure 5:**
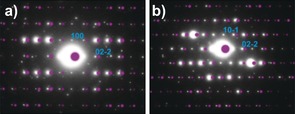
Comparison of the experimental TEM electron diffraction patterns of **flsZ** with patterns simulated from the PXRD‐derived crystal structure of **flsZ**. The patterns were indexed to the <011> (a) and <111> (b) zone axes of this structure. Simulated patterns are shown in purple and offset slightly relative to the experimental electron diffraction patterns for clarity. The simulated diffraction patterns were modeled assuming the kinematic model of electron scattering. The effects of dynamic scattering in the experimental patterns lead to appearance of reflections that are absent under the kinematic model.^32^

The correctness of the crystal structure determination was further supported by performing TEM analysis on the **flsZ** powder particles and determining which of the predicted structures were consistent with the resulting electron diffraction patterns. Structure number 3 was found to be the best match to the TEM data from the low energy predicted crystal structures. Moreover, the experimental electron diffraction patterns were a satisfying match to patterns simulated from the CSP‐PXRD‐derived crystal structure of **flsZ** (Figure 5). The combined assignment based on both PXRD and TEM electron diffraction data provides a high level of confidence in the CSP structure determination.

The principal intermolecular interactions present in the **flsZ** crystal structure (and which are also present in the other low‐energy predicted structures) are charge‐assisted O−H⋅⋅⋅O hydrogen bonds between the hydroxy protons and the carboxylate oxygen atoms of the neighboring **flsZ** molecules (Figure 4 a).

**Figure 6:**
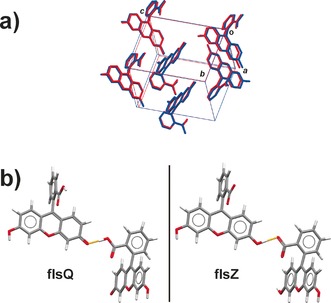
a) Overlay of the experimental crystal structure of **flsQ** (blue) and the lowest energy predicted structure of **flsZ** (red). Hydrogen atoms were omitted for clarity. b) A shift in the hydrogen position causes the interconversion between **flsQ** (left) and **flsZ** (right).

Careful inspection of the predicted structures revealed that the lowest‐energy structure of **flsZ** showed a close similarity with the experimentally reported structure of **flsQ**. An overlay of the two structures (Figure 6 a) shows a very close alignment of heavy atom positions. The observed quinoid and predicted zwitterionic crystal structures are related by switching of the hydrogen position in the COOH⋅⋅⋅O (carbonyl) intermolecular hydrogen bond (Figure 6 b). In fact, an attempt to perform a DFT geometry optimization of the experimental **flsQ** crystal structure using the PBE functional^48^ led to a proton shift and transition of the molecule into the **flsZ** tautomeric form. The most likely reason for this incorrect modeling of the hydrogen bonding is the known feature of the semilocal DFT functionals to underestimate, or even completely suppress, the energy barriers for hydrogen bond proton transfers, even when dispersion correction is applied.^49^ The incorrect description of hydrogen bonding by the PBE functional may also cause certain errors in the energy ranking of the predicted structures. The accuracy of the calculations could have been improved by using a hybrid functional such as B3LYP^50^ or PBE0,^50^, ^51^ although the cost of performing such calculations with the plane‐wave basis set would be prohibitive.

### Crystal structure prediction for unsolvated flsL

A variety of crystallization methods were applied in an attempt to crystallize **flsL** without the presence of guest molecules in its crystal structure. All experiments, however, resulted in the formation of crystalline **flsQ** or **flsZ**, or in the crystallization of the solvated forms of **flsL**. Desolvation of **flsL**:acetone monosolvate Form II was shown to produce an amorphous phase that subsequently crystallizes into **flsQ** (Supporting Information, Figure S25). The apparent difficulty to crystallize pure **flsL** led us to investigate this phenomenon from a theoretical perspective by performing crystal structure prediction to assess the possible crystal packing of pure **flsL**. Comparison of the crystal energy landscapes formed by **flsL** and **flsZ** tautomers revealed that the lowest energy predicted structure of **flsL** is 21.1 kJ mol^−1^ higher in lattice energy then the experimental structure of **flsZ** (for complete crystal energy landscape of both tautomeric forms, see the Supporting Information, Figure S30). Such a large energy difference between the two forms indicates that crystalline **flsL** is thermodynamically unstable with respect to **flsZ** in the solid state. The calculated energy difference is well outside the energetic range of observed polymorphism, where lattice energy differences are typically well under 10 kJ mol^−1^,^52^ explaining the difficulty of crystallizing the lactoid tautomer.

### Solid‐state NMR measurements and chemical shift calculations

The method of solid‐state NMR has acquired considerable importance as a complementary structural technique to X‐ray diffraction. It has been used to study hydrogen bonding,^53^ ion mobility,^54^ and static and dynamic disorder in solids.^55^, ^56^ Although solid‐state NMR is invaluable as a purely experimental technique, results obtained by this method can be greatly reinforced through the use of NMR chemical shielding calculations. The latter has been greatly facilitated by the development of the gauge including projector augmented waves (GIPAW)^57^ method, currently implemented in several plane‐wave packages including CASTEP.^58^ GIPAW calculations can display an accuracy of 1‐2 ppm for modeling ^13^C NMR spectra.^44^, ^59^ Such a high accuracy of NMR calculations establishes it as a powerful technique for assignment of predicted structures to experimental data,^60^ and makes it particularly effective for distinguishing tautomeric forms.^61^

Since the hydrogen bonding in the crystal structures of **flsQ** and **flsZ** differs only in the position of the hydrogen atom, interconversion of the two tautomeric forms in the solid state may occur as a result of a proton shift across the hydrogen bond. Since crystal structures of both tautomers have been determined using PXRD, a method that is not sensitive to determination of hydrogen atom positions, solid‐state NMR measurements were performed in order to unambiguously assign the molecular tautomers to the different color forms of fluorescein.

Solid‐state ^13^C NMR spectra were recorded for the samples of pure red and yellow fluorescein (assumed to contain **flsQ** and **flsZ** forms respectively) as well as for four solvates of **flsL** (both polymorphs of acetone monosolvate, dioxane hemisolvate and dioxane hemipentasolvate). The solvate materials were readily assigned to contain the **flsL** tautomer based on the NMR signal at 85–88 ppm, which could only correspond to the quaternary carbon atom in the lactone ring. Such a high‐field signal cannot correspond to either **flsQ** or **flsZ** since these molecules contain only sp^2^‐hybridized conjugated carbon atoms with signals further downfield. The spectra of **flsQ** and **flsZ**, on the other hand, display close similarity that makes the assignment of these tautomers more difficult. In order to resolve this ambiguity, and aid the full assignment of NMR spectra, CASTEP GIPAW calculations were performed on the crystal structures of the corresponding materials. The calculated NMR tensors were related to the experimental chemical shifts using the linear regression procedure described in the Supporting Information.

**Figure 7:**
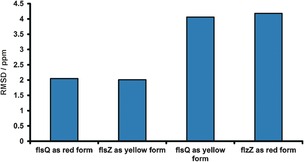
The root‐mean‐square difference (RMSD) between the calculated and experimental ^13^C chemical shifts for the red and yellow forms of fluorescein modeled both as **flsZ** and **flsQ**.

To perform the calculations the crystal structures of red and yellow fluorescein were geometry‐optimized, with hydrogen atom position constrained to represent either the **flsQ** or the **flsZ** tautomer, and the NMR parameters were computed for each. The calculated chemical shifts were then compared to the experimental spectra and it was unambiguously shown that the red and yellow forms contain **flsQ** and **flsZ** tautomers, respectively (Figure 7).

During the analysis of the ^13^C NMR spectra of the **flsL** solvates it was noticed that the chemical shift of the carbonyl carbon in the dioxane hemipentasolvate (168.47 ppm) is approximately 3 ppm lower than that in the dioxane hemisolvate and acetone monosolvate (172.54 and 172.62 ppm, respectively). Inspection of the corresponding crystal structures (Figure 4 b–d) suggests that the variations in chemical shifts are caused by the differences in hydrogen bonding: in the acetone monosolvate and dioxane hemisolvate the carbonyl oxygen is hydrogen‐bonded to the hydroxy group of another **flsL** molecule, while in the dioxane hemipentasolvate such a hydrogen bond is absent. This effect of hydrogen‐bonding on the NMR chemical shift is very well modeled by the GIPAW calculation: the carbonyl shifts calculated using the regression equation are 167.77, 171.97, and 171.45 ppm for the dioxane hemipentasolvate, dioxane hemisolvate, and acetone monosolvate Form I, respectively. This result illustrates the excellent accuracy of the GIPAW method in modeling the effect of structural features such as hydrogen bonds on the NMR parameters.

### Optical properties of fls tautomers in the solid state

Once the agreement between the tautomers and solid forms of fluorescein had been established, the origins of the color of these solid forms remained to be explained. Out of the three available tautomeric forms only two were isolated in the solid state in their pure form: the red fluorescein containing molecules of **flsQ** and yellow fluorescein, **flsZ**. The molecule of **flsL**, as the calculations have shown, cannot form a thermodynamically stable crystal structure without other guest molecules present. Nonetheless, the lactoid form produces a number of multi‐component solids including dioxane hemi‐ and hemipenta‐ solvates, acetone and methanol monosolvates as well as cocrystals with acridine, phenanthridine, and pyrazine.

The unifying structural feature of all the known crystal forms of **flsL** is that the guest molecules in these structures act as hydrogen bond acceptors interacting with the hydroxy groups of **fls**. These materials are colored yellow, with the exception of the pyrazine cocrystal, which is colored green. By contrast, a similar compound, diacetylfluorescein, crystallizes as a single‐component colorless solid in its lactoid form. Therefore, a possible explanation for the generation of color in the lactoid fluorescein samples may be the electronic interactions of **flsL** with the coformer molecules. Colored cocrystals consisting of components that are colorless in their pure form have previously been reported,^20^ although these normally contain coformers with extended conjugated systems. It is very unlikely that the presence of a dioxane or acetone molecule in the crystal structures would lead to a dramatic change in color.

Another possible explanation for the origin of color is the formation of a yellow **flsZ** layer on the surface of the lactoid crystals. The main argument in favor of this hypothesis is the apparent color dependence on the particle size: samples with smaller particle size have greater surface to bulk ratio and display brighter colors.

As a first step in the analysis of fluorescein optical properties, the solid‐state UV/Vis spectra of all available solid forms were recorded and the corresponding band‐gaps were determined. The value of the band gap determines the energy cut‐off below which the light photons will be reflected. Therefore, knowledge of the band‐gap is critical for understanding the color properties of the material. Theoretical density of states calculations were performed using the code OptaDOS^62^, ^63^ in parallel with the experimental measurements. The calculated band gaps are noticeably lower than the experimental values (Figure 9), which is a known behavior of semilocal DFT functionals such as PBE. Most importantly, however, the calculated values show good correlation with experiment meaning that theoretical band structure can provide important insights into the optical properties of fluorescein crystal forms. Overall, the band gaps of the materials increase in the order **flsQ**<**flsZ**<**flsL**<diacetylfluorescein (a full summary of the measured band gaps is given in the Supporting Information, Table S9 and Figures S41–S50).

The band gaps of **flsQ** and **flsZ** are consistent with their observed colors, but this is not the case for solids containing **flsL**, where the majority of materials have band gaps well above the visible light energy range (1.5–3.3 eV). Materials with such high band gaps are expected to be white, as is demonstrated by the white solid of diacetylfluorescein. The band gaps of both dioxane solvates of **flsL** are essentially the same as that for diacetylfluorescein. However, these solvates display a bright yellow color in powder form. The only material that has a sufficiently low band gap to reflect a certain proportion of visible light is the acridine cocrystal.

**Figure 8:**
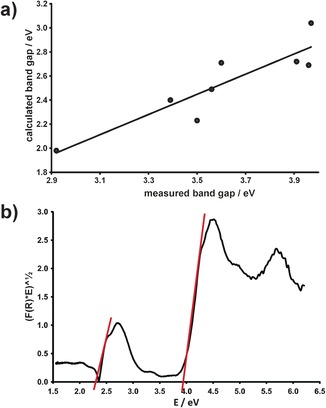
a) The correlation between computed and experimental band‐gaps of the **flsL** solid forms. b) Tauc plot^66^ constructed from the reflectance spectrum of the **flsL**:dioxane hemisolvate. The linear regions crossing the abscissa at 2.3 and 3.9 eV correspond to the band gaps of the zwitterion impurity and the bulk material, respectively.

**Figure 9:**
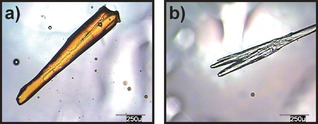
A crystal of **flsL**:dioxane hemipentasolvate a) before and b) after dissolution of the surface layer. The discoloration of the crystal upon removal of the surface layer is evident.

Detailed analysis of the spectra of lactoid **fls** solid forms revealed that they all contain a weak yet reproducible feature corresponding to the electronic transition of 2.30±0.06 eV (Figure 8 b), which is very similar to the band gap of pure **flsZ**. This observation suggests that an impurity of zwitterionic fluorescein is present in the lactoid samples. Visible‐light spectroscopy, however, cannot distinguish between a surface layer of the zwitterion impurity and randomly distributed impurity within the bulk structure in the form of crystal defects. This ambiguity has been resolved by dissolving the surface layer of the **flsL**:dioxane hemipentasolvate single crystal: the originally yellow crystal was placed in a mixture of dioxane with silicon oil. Observation of this crystal under a microscope has shown complete loss of color (Figure 9) thus proving that the zwitterion impurity is concentrated on the crystal surface. It is noteworthy that the yellow color is rapidly restored when the crystal is taken out of solution and exposed to air.

**Figure 10:**
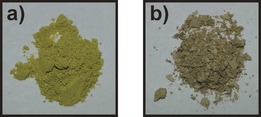
The samples of pyrazine cocrystal a) with and b) without an **flsZ** surface layer. Both materials have identical bulk crystal structures, as confirmed by PXRD.

Another solid for which it has been possible to remove the zwitterion surface layer is the pyrazine cocrystal of **flsL**. This cocrystal is prepared in a mechanochemical process that produces the yellow‐green powder. Adding an excess of pyrazine into the grinding jar, however, resulted in the formation of a greyish powder with reduced **flsZ** surface content (Figure 10). Both the green and the grey material have identical crystal structures, as confirmed by PXRD, with the green product having significantly greater amount of **flsZ** impurity at the surface (Supporting Information, Figures S46 and S47). The connection between the lack of a colored surface layer and the use of an excess of pyrazine in the synthesis of the cocrystal is so far not entirely understood. The color of the material is affected by factors such as frequency of grinding, duration of the experiment and amount of liquid added to the reaction mixture. Investigation of all these factors lies outside the scope of this study. A detailed experimental study is, however, underway and its results will be reported separately.

The combination of solid‐state UV/Vis measurements with analysis of **flsL**:dioxane hemipentasolvate and **flsL**:pyrazine crystal surface behavior has allowed us to conclude that the origin of the color of the lactoid fluorescein crystals is the yellow surface layer that consists of **flsZ** molecules. To fully understand the optical properties of the materials, however, the contribution of the bulk crystal structure has to be considered. Among the available crystal forms of **flsL** there is a considerable variation in the experimentally measured band gaps. More specifically, cocrystals of **flsL** with highly conjugated coformers (pyrazine, acridine, and phenanthridine) display significantly lower band gaps than the dioxane solvates. This observation clearly suggests that cocrystal coformers have a pronounced effect on the optical properties of the materials. The available experimental techniques, however, do not provide an explanation for this effect. The computational analysis of the band structure of the material, on the other hand, can readily provide such information.

The most direct way to establish the role of the coformer in the generation of the band gap is to calculate the partial density of states (PDOS) alongside the full DOS calculation. The PDOS analysis presents a means of partitioning the full DOS into contributions from different chemical species present in the crystal structure. In the present study, the band structure was partitioned into contributions from fluorescein and the coformer molecules. The **fls** PDOS was then compared to the full DOS in order to establish whether the coformer molecular orbitals are involved in the formation of the cocrystal frontier bands (HOMO or LUMO). The outcome of the calculation was in excellent agreement with the experimental observations: materials with the highest observed band gaps (acetone solvate and both dioxane solvates) showed no contribution to the frontier bands from the coformer molecules. Density of states analysis of the cocrystals involving pyrazine, phenanthridine, and acridine, on the other hand, revealed that the LUMO of these materials is fully localized on the coformer molecules: the highly conjugated coformer molecules have LUMO π‐orbitals that are lower in energy then the LUMO of **flsL**. Therefore, the presence of such coformers lowers the band gap of the material by 0.66, 0.56, and 0.33 eV for acridine, pyrazine, and phenanthridine, respectively (Supporting Information, Figures S57–59). It should be noted, however, that the band gaps of the cocrystals, although lower than those of the solvates, still lie outside of the visible light range. It is, therefore, unlikely that coformer molecules have a significant effect on the color properties of the cocrystals of **flsL**. The color is mainly determined by the presence or absence of the **flsZ** surface layer. The calculations nonetheless confirm that cocrystallization may be used as a method of altering the band gap of a material, thus altering its optical and electronic properties.

### Formation mechanism of the flsZ surface layer

The combination of experimental and computational studies has allowed us to establish the role of the zwitterion surface layer in the color properties of the lactoid **fls** cocrystals and solvates. To gain a complete understanding of the phenomenon, however, we need to establish the reasons for the formation of this surface layer. Experiments with gray samples of **flsL**:pyrazine cocrystal have shown that the yellow surface layer gradually appears when in contact with air. The observed effect is unlikely to be caused by an interaction with air oxygen, since the transition of **flsL** to **flsZ** is not an oxidation process. Most likely, the transformation is facilitated by water present in the air. Indeed, our experiments have shown that the crystals remain grey for days when stored under zero humidity conditions (in a desiccator filled with phosphorus pentoxide as a drying agent). Exposure of the same material to 98 % relative humidity, however, leads to the formation of the surface layer within 30 min.

Dissociation of cocrystals catalyzed by air humidity has previously been reported.^64^, ^65^ The process begins with condensation of the water vapor on the crystal surface, where it leads to partial dissolution of the material and the formation of saturated solution of **fls** in water. In aqueous solution the **flsL** molecules will form an equilibrium with the **flsZ** tautomer. Since **flsZ** has a lower lattice energy than **flsL**, it is likely to be the least soluble species present in solution and is expected to crystallize preferentially, generating a colored layer on the crystal surface.

## Conclusion

A combination of experimental and computational methods was used to rationalize the crystallographic behavior and color properties of **fls** tautomeric forms in the solid state. Three crystal structures have been determined: **flsZ** (yellow form) and two solvates of **flsL** with 1,4‐dioxane. The structure of **flsZ** has been determined using a combined CSP/PXRD approach and further supported by TEM analysis. The determination of this crystal structure gives yet another example where a combination of experimental techniques such as PXRD,^42^, ^43^ TEM,^33^, ^34^ and solid‐state NMR^60^ with CSP proves to be exceptionally useful.

The previously reported crystal structure of **flsQ** 27 and the new structure of **flsZ** have both been determined using PXRD, a technique insensitive to the positions of hydrogen atoms. To fully resolve any ambiguities in the assignment of these two tautomeric forms, solid‐state NMR spectroscopy combined with DFT GIPAW calculations were employed. The NMR studies fully supported the generally accepted assignment of **flsQ** and **flsZ** as the red and yellow forms, respectively.

Solid‐state UV/Vis spectroscopy was used to measure the band gaps of the materials and correlate them with the observed colors. The band gaps of all lactoid samples were consistently high, comparable to the band gap of a white solid of diacetylfluorescein, suggesting that the fluorescein samples should also be colorless. More detailed analysis of the spectra of the lactoid forms, however, revealed the presence of an impurity with a band gap of approximately 2.3 eV, consistent with the gap of pure **flsZ**. Further experiments with larger crystals of **flsL** dioxane hemipentasolvate showed that the zwitterion impurity is concentrated on the crystal surface. Evidence has also been found that the formation of this layer is catalyzed by water present in the air. Water condensing on the crystals dissolves the surface layer, forming a saturated solution in which the lactoid and zwitterionic forms of fluorescein are in equilibrium. The zwitterionic form, which has a lower lattice energy and is, therefore, less soluble, crystallizes out of the solution and generates the yellow coating on the surface of the crystals.

The theoretical band structure calculations allowed us to establish the role of crystal coformers in the modification of color properties of cocrystals. It was shown that the coformers with extended π‐conjugation have a LUMO lower than that of **flsL**. Incorporation of such a coformer into the crystal structure therefore leads to the lowering of the band gap of the material, the effect being most pronounced for the acridine cocrystal.

In this work we have applied a combination of experimental and computational methods to characterize the effects of tautomerism and crystal packing on the optical properties of fluorescein. We expect that, with the ever‐increasing computing power and the progress in the development of computational methods, computational studies of organic solids will soon be standard procedure in materials research and in the development of new materials with targeted properties.

## Supporting information

As a service to our authors and readers, this journal provides supporting information supplied by the authors. Such materials are peer reviewed and may be re‐organized for online delivery, but are not copy‐edited or typeset. Technical support issues arising from supporting information (other than missing files) should be addressed to the authors.

Supplementary

## References

[1] T. Mayer , U. Weiler , C. Kelting , D. Schlettwein , S. Makarov , D. Wöhrle , O. Abdallah , M. Kunst , W. Jaegermann , Sol. Energy Mater. Sol. Cells 2007, 91, 1873–1886.

[2] H. Mustroph , M. Stollenwerk , V. Bressau , Angew. Chem. Int. Ed. 2006, 45, 2016–2035; 10.1002/anie.200502820 16518782

[3] B. L. Kaul , Rev. Prog. Color. Relat. Top. 2008, 23, 19–35.

[4] World Dyes & Organic Pigments Market, can be found under http://www.reportlinker.com/p02900236-summary/World-Dyes-Organic-Pigments-Market.html, **2015**.

[5] M. C. Payne , T. A. Arias , J. D. Joannopoulos , Rev. Mod. Phys. 1992, 64, 1045–1097.

[6] H. Xiao , J. Tahir-Kheli , W. A. Goddard , J. Phys. Chem. Lett. 2011, 2, 212–217.

[7] A. N. Sokolov , S. Atahan-Evrenk , R. Mondal , H. B. Akkerman , R. S. Sánchez-Carrera , S. Granados-Focil , J. Schrier , S. C. B. Mannsfeld , A. P. Zoombelt , Z. Bao , A. Aspuru-Guzik , Nat. Commun. 2011, 2, 437. 21847111 10.1038/ncomms1451 PMC3366639

[8] J. Hachmann , R. Olivares-Amaya , A. Jinich , A. L. Appleton , M. A. Blood-Forsythe , L. R. Seress , C. Román-Salgado , K. Trepte , S. Atahan-Evrenk , S. Er , S. Shrestha , R. Mondal , A. Sokolov , Z. Bao , A. Aspuru-Guzik , Energy Environ. Sci. 2014, 7, 698-704.

[9] F. Blanc , D. S. Middlemiss , Z. Gan , C. P. Grey , J. Am. Chem. Soc. 2011, 133, 17662–17672. 21916439 10.1021/ja2053557

[10] K. A. See , M. Leskes , J. M. Griffin , S. Britto , P. D. Matthews , A. Emly , A. Van der Ven , D. S. Wright , A. J. Morris , C. P. Grey , R. Seshadri J. Am. Chem. Soc. 2014, 136, 16368–16377. 25384082 10.1021/ja508982p PMC4353022

[11] J.-I. Hong , J. Choi , S. S. Jang , J. Gu , Y. Chang , G. Wortman , R. L. Snyder , Z. L. Wang , Nano Lett. 2012, 12, 576–581. 22214217 10.1021/nl203033h

[12] A.-X. Yin , W.-C. Liu , J. Ke , W. Zhu , J. Gu , Y.-W. Zhang , C.-H. Yan , J. Am. Chem. Soc. 2012, 134, 20479–20489. 23181397 10.1021/ja3090934

[13] D. A. Bardwell , C. S. Adjiman , Y. A. Arnautova , E. Bartashevich , S. X. M. Boerrigter , D. E. Braun , A. J. Cruz-Cabeza , G. M. Day , R. G. Della Valle , G. R. Desiraju , B. P. van Eijck , J. C. Facelli , M. B. Ferraro , D. Grillo , M. Habgood , D. W. M. Hofmann , F. Hofmann , K. V. J. Jose , P. G. Karamertzanis , A. V. Kazantsev , J. Kendrick , L. N. Kuleshova , F. J. J. Leusen , A. V Maleev , A. J. Misquitta , S. Mohamed , R. J. Needs , M. A. Neumann , D. Nikylov , A. M. Orendt , R. Pal , C. C. Pantelides , C. J. Pickard , L. S. Price , S. L. Price , H. A. Scheraga , J. van de Streek , T. S. Thakur , S. Tiwari , E. Venuti , I. K. Zhitkov , Acta Crystallogr. Sect. A 2011, 67, 535–551. 10.1107/S0108768111042868 PMC3222142 22101543

[14] S. L. Bekö , S. M. Hammer , M. U. Schmidt , Angew. Chem. Int. Ed. 2012, 51, 4735–4738; 10.1002/anie.201109082 22461339

[15] J. L. Teteruk , J. Glinnemann , T. E. Gorelik , A. Linden , M. U. Schmidt , Acta Crystallogr. Sect. A 2014, 70, 296–305. 10.1107/S2052520613031636 24675599

[16] F. Zhang , F. Zhang , J. Qun , S. Pan , Z. Yang , D. Jia , Phys. Chem. Chem. Phys. 2015, 17, 10489–10496. 25803617 10.1039/c5cp00864f

[17] K. Hunger , Rev. Prog. Color. Relat. Top. 2008, 29, 71–84.

[18] L. Yu , G. A. Stephenson , C. A. Mitchell , C. A. Bunnell , S. V. Snorek , J. J. Bowyer , T. B. Borchardt , J. G. Stowell , S. R. Byrn , J. Am. Chem. Soc. 2000, 122, 585–591.

[19] L. Yu , Acc. Chem. Res. 2010, 43, 1257–1266. 20560545 10.1021/ar100040r

[20] J. R. G. Sander , D.-K. Bučar , R. F. Henry , J. Baltrusaitis , G. G. Z. Zhang , L. R. MacGillivray , J. Pharm. Sci. 2010, 99, 3676–3683. 20574998 10.1002/jps.22229

[21] M. L. Cheney , G. J. McManus , J. A. Perman , Z. Wang , M. J. Zaworotko , Cryst. Growth Des. 2007, 7, 616–617.

[22] D. Yan , A. Delori , G. O. Lloyd , T. Friščić , G. M. Day , W. Jones , J. Lu , M. Wei , D. G. Evans , X. Duan , Angew. Chem. Int. Ed. 2011, 50, 12483–12486; 10.1002/anie.201106391 22065645

[23a] D. Yan , A. Delori , G. O. Lloyd , B. Patel , T. Friščić , G. M. Day , D.-K. Bučar , W. Jones , J. Lu , M. Wei , D. G. Evans , X. Duan , CrystEngComm 2012, 14, 5121–5123;

[23b] A. Pallipurath , J. M. Skelton , A. Delori , C. Duffy , A. Erxleben , W. Jones , CrystEngComm 2015, 17, 7684–7692..

[24] R. Markuszewski , H. Diehl , Talanta 1980, 27, 937–946. 18962830 10.1016/0039-9140(80)80125-1

[25] U. Anthoni , C. Christophersen , P. H. Nielsen , A. Püschl , K. Schaumburg , Struct. Chem. 1995, 6, 161–165.

[26] A. J. Cruz-Cabeza , C. R. Groom , CrystEngComm 2011, 13, 93–98.

[27] M. Tremayne , B. M. Kariuki , K. D. M. Harris , Angew. Chem. Int. Ed. Engl. 1997, 36, 770–772;

[28] K. D. Knudsen , P. Pattison , A. N. Fitch , R. J. Cernik , Angew. Chem. Int. Ed. 1998, 37, 2340–2343; 10.1002/(SICI)1521-3773(19980918)37:17<2340::AID-ANIE2340>3.0.CO;2-Z 29710942

[29] R. S. Osborn , D. Rogers , Acta Crystallogr. Sect. A 1975, 31, 359–364.

[30] I. N. Polyakova , Z. A. Starikova , B. V. Parusnikov , I. A. Krasavin , G. M. Dobryakova , B. V. Zhdanov , J. Struct. Chem. 1984, 25, 752–757.

[31] D.-K. Bučar , S. Filip , M. Arhangelskis , G. O. Lloyd , W. Jones , CrystEngComm 2013, 15, 6289–6291.

[32] M. D. Eddleston , E. G. Bithell , W. Jones , J. Pharm. Sci. 2010, 99, 4072–4083. 20665849 10.1002/jps.22220

[33] M. D. Eddleston , K. E. Hejczyk , E. G. Bithell , G. M. Day , W. Jones , Chem. Eur. J. 2013, 19, 7883–7888. 23592497 10.1002/chem.201204369

[34] M. D. Eddleston , K. E. Hejczyk , E. G. Bithell , G. M. Day , W. Jones , Chem. Eur. J. 2013, 19, 7874–7882. 23592444 10.1002/chem.201204368

[35] D.-K. Bučar , J. A. Elliott , M. D. Eddleston , J. K. Cockcroft , W. Jones , Angew. Chem. Int. Ed. 2015, 54, 249–253; 10.1002/anie.201408894 25370777

[36] T. G. Cooper , K. E. Hejczyk , W. Jones , G. M. Day , J. Chem. Theory Comput. 2008, 4, 1795–1805. 26620182 10.1021/ct800195g

[37] G. M. Day , T. G. Cooper , CrystEngComm 2010, 12, 2443–2453.

[38] D.-K. Bučar , G. M. Day , I. Halasz , G. G. Z. Zhang , J. R. G. Sander , D. G. Reid , L. R. MacGillivray , M. J. Duer , W. Jones , Chem. Sci. 2013, 4, 4417–4425.

[39] S. L. Price , Chem. Soc. Rev. 2014, 43, 2098–2111. 24263977 10.1039/c3cs60279f

[40] G. M. Day , Crystallogr. Rev. 2011, 17, 3–52.

[41] G. R. Desiraju , Angew. Chem. Int. Ed. Engl. 1995, 34, 2311–2327;

[42] N. Panina , R. van de Ven , P. Verwer , H. Meekes , E. Vlieg , G. Deroover , Dye. Pigment. 2008, 79, 183–192.

[43] M.-A. Perrin , M. A. Neumann , H. Elmaleh , L. Zaske , Chem. Commun. 2009, 3181–3183. 10.1039/b822882e 19587906

[44] M. Baias , C. M. Widdifield , J.-N. Dumez , H. P. G. Thompson , T. G. Cooper , E. Salager , S. Bassil , R. S. Stein , A. Lesage , G. M. Day , L. Emsley , Phys. Chem. Chem. Phys. 2013, 15, 8069–8080. 23503809 10.1039/c3cp41095a

[45] M. Baias , J.-N. Dumez , P. H. Svensson , S. Schantz , G. M. Day , L. Emsley , J. Am. Chem. Soc. 2013, 135, 17501–17507. 24168679 10.1021/ja4088874

[46] C. F. Macrae , I. J. Bruno , J. A. Chisholm , P. R. Edgington , P. McCabe , E. Pidcock , L. Rodriguez-Monge , R. Taylor , J. van de Streek , P. A. Wood , J. Appl. Crystallogr. 2008, 41, 466–470.

[47] H. M. Rietveld , Acta Crystallogr. 1967, 22, 151–152.

[48] J. P. Perdew , K. Burke , M. Ernzerhof , Phys. Rev. Lett. 1996, 77, 3865–3868. 10062328 10.1103/PhysRevLett.77.3865

[49] S. Sadhukhan , D. Muñoz , C. Adamo , G. E. Scuseria , Chem. Phys. Lett. 1999, 306, 83–87.

[50] A. D. Becke , J. Chem. Phys. 1993, 98, 5648–5651.

[51] M. Ernzerhof , G. E. Scuseria , J. Chem. Phys. 1999, 110, 5029–5036.

[52] J. Nyman , G. M. Day , CrystEngComm 2015, 17, 5154–5165.

[53] J. R. Yates , T. N. Pham , C. J. Pickard , F. Mauri , A. M. Amado , A. M. Gil , S. P. Brown , J. Am. Chem. Soc. 2005, 127, 10216–10220. 16028932 10.1021/ja051019a

[54] G. R. Goward , M. F. H. Schuster , D. Sebastiani , I. Schnell , H. W. Spiess , J. Phys. Chem. B 2002, 106, 9322–9334.

[55] A. P. M. Kentgens , Geoderma 1997, 80, 271–306.

[56] S. Cadars , A. Lesage , C. J. Pickard , P. Sautet , L. Emsley , J. Phys. Chem. A 2009, 113, 902–911. 19133744 10.1021/jp810138y

[57] C. J. Pickard , F. Mauri , Phys. Rev. B 2001, 63, 245101.

[58] S. J. Clark , M. D. Segall , C. J. Pickard , P. J. Hasnip , M. I. J. Probert , K. Refson , M. C. Payne , Z. Kristallogr. - Cryst. Mater. 2005, 220, 567–570.

[59] J. C. Johnston , R. J. Iuliucci , J. C. Facelli , G. Fitzgerald , K. T. Mueller , J. Chem. Phys. 2009, 131, 144503. 19831448 10.1063/1.3225270 PMC2771050

[60] E. Salager , G. M. Day , R. S. Stein , C. J. Pickard , B. Elena , L. Emsley , J. Am. Chem. Soc. 2010, 132, 2564–2566. 20136091 10.1021/ja909449k

[61] X. Li , A. D. Bond , K. E. Johansson , J. van de Streek , Acta Crystallogr. Sect. A 2014, 70, 563. 10.1107/S2053229614015356 PMC4174016 25093360

[62] A. J. Morris , R. J. Nicholls , C. J. Pickard , J. R. Yates , Comput. Phys. Commun. 2014, 185, 1477–1485.

[63] R. J. Nicholls , A. J. Morris , C. J. Pickard , J. R. Yates , J. Phys. Conf. Ser. 2012, 371, 012062.

[64] M. Arhangelskis , G. O. Lloyd , W. Jones , CrystEngComm 2012, 14, 5203–5208.

[65] M. D. Eddleston , R. Thakuria , B. J. Aldous , W. Jones , J. Pharm. Sci. 2014, 103, 2859–2864. 24481664 10.1002/jps.23865

[66] J. Tauc , R. Grigorovici , A. Vancu , Phys. Status Solidi 1966, 15, 627–637.

