# Supplementary material for: Rationalization of the Color Properties of Fluorescein in the Solid State: A Combined Computational and Experimental Study
Source: Chemistry. 2016 Jun 15;22(29):10065–73. doi: 10.1002/chem.201601340 (PMC4982065; doi:10.1002/chem.201601340)
Supplement: Supplementary file 1 — Supplementary [file CHEM-22-10065-s001.pdf]

CHEMISTRY
A European Journal

Supporting Information

Rationalization of the Color Properties of Fluorescein in the Solid State: A Combined Computational and Experimental Study

Mihails Arhangelskis,[a] Mark D. Eddleston,[a] David G. Reid,[a] Graeme M. Day,[b] Dejan-Krešimir Bučar,[c] Andrew J. Morris,[d] and William Jones*[a]

chem_201601340_sm_miscellaneous_information.pdf

Table of contents

1. MATERIALS 3

2. EXPERIMENTAL TECHNIQUES 3

3. COMPUTATIONAL STUDIES 6

4. CRYSTALLOGRAPHIC DATA 8

5. THERMAL ANALYSES 18

6. CRYSTAL STRUCTURE PREDICTION (CSP) 24

7. SOLID-STATE ^{13}C NMR SPECTROSCOPY 28

8. OPTICAL SPECTROSCOPY AND CALCULATIONS 36

9. REFERENCES 47

1. Materials

Fluorescein (95%), diacetylfluorescein (99%), pyrazine (99%) and phenanthridine (98%) were purchased from Sigma Aldrich. Acridine (98%) was obtained from Alfa Aesar. All materials were used as received without further purification.

2. Experimental techniques

Preparation of yellow (zwitterionic) fluorescein

Diacetylfluorescein (1.00 g, 2.11 mmol) was added to a solution of sodium hydroxide (400 mg, 10 mmol) in ethanol. The resulting solution was refluxed for 20 min and then cooled to room temperature. Glacial acetic acid was added to neutralize the remaining base and crystallization was induced by addition of deionized water. The precipitate was collected by vacuum filtration in the form of a fine yellow powder. The identity of the material was verified by PXRD, 13C solid-state NMR and elemental analysis.

Preparation of flsL:acetone monosolvate

Fluorescein red form, flsQ, (0.50 g, 1.50 mmol) was added to acetone (150 mL0 and the mixture was stirred until the solid had completely dissolved and the solution changed color to yellow. The resulting solution was evaporated under reduced pressure to produce a yellow powder of flsL:acetone monosolvate form II.
A sample of form II (100 mg, 2.56 mmol) was slurried in acetone (0.6 ml) for 24 hours. The mixture was dried and the resulting solid was identified as form I of flsL:acetone monosolvate (CSD FLSCAC).[1] Both polymorphs of the solvate were characterized by PXRD, DSC, TGA and ^{13}C solid-state NMR.

Preparation of flsL:1,4-dioxane hemipentasolvate

Fluorescein red form, flsQ, (1.00 g, 3.01 mmol) was added to 1,4-dioxane (150 mL) and the mixture was refluxed until the solid had completely dissolved. During the dissolution, fluorescein changed tautomeric form, which resulted in the color changing from red to yellow. A small aliquot of this solution was slowly evaporated to produce crystals for single crystal X-ray diffraction. The bulk solution was evaporated under reduced pressure to produce a yellow powder of flsL:1,4-dioxane hemipentasolvate. This material was analyzed by PXRD, DSC, TGA and ^{13}C solid-state NMR.

Preparation of flsL:1,4-dioxane hemisolvate

A sample of flsL:1,4-dioxane hemipentasolvate (200 mg, 0.369 mmol) was heated at 80 °C for 15 min. During the heating process, partial desolvation occurred, resulting in formation of a solvate with a different stoichiometry. The crystal structure of this material was determined from PXRD data. Prolonged heating of the flsL:1,4-dioxane hemisolvate at temperatures above 150 °C resulted in complete desolvation and formation of flsQ.

Preparation of fluorescein cocrystals

Cocrystals of flsL with pyrazine, acridine and phenanthridine were prepared according to the published method:[2] flsQ (100 mg, 0.301 mmol) and a stoichiometric amount of the coformer were mixed in a grinding jar to which was added 50 μl of nitromethane and two 7 mm stainless steel grinding balls. The mixture was ground for 30 min at 30 Hz frequency in a Retch MM200 mill. The resulting solids were analyzed by PXRD. Cocrystals were obtained in the form of colored powders. In the case of the pyrazine cocrystal an additional experiment was performed where pyrazine was taken in five-fold excess (120.5 mg, 1.50 mmol), which resulted in the formation of a pale-grey solid.

Powder X-ray diffraction

The PXRD patterns were recorded on a Stoe StadiP diffractometer in transmission geometry using monochromated CuKα1 radiation generated at 40 kV and 30 mA. Samples were placed in 0.5 mm borosilicate glass capillaries. For phase identification the data collection was performed over a 2-60.0° 2θ range (0.5° step, 5 s/step). The patterns used for crystal structure determination were collected over a 2-60° 2θ range (continuous mode, 0.5° step, 20 s/step). The powder patterns were visualized using the *X'Pert HighScore Plus* v2.2 software.

The crystal structures of the flsL:dioxane hemisolvate and flsL:acetone monosolvate form II were determined from PXRD data. The powders pattern were indexed using the program *DICVOL 06*,[3] while the interface of the program *DASH 3.2*[4] was used for space group determination. The power pattern of flsL:acetone solvate was indexed with a monoclinic cell, the systematic absences unambiguously suggested $P2_1/c$ symmetry. The pattern of flsL:dioxane hemisolvate was indexed with a triclinic cell with possible space groups restricted to *P1* and *P−1*.. T.. Since these space groups cannot be distinguished based on systematic absences, structure solution was performed with the lowest symmetry, *P1*.

The remainder of structure determination was performed in the program *TOPAS Academic 4.1*.[5] A Pawley refinement[6] procedure was used to determine the optimal values for the zero point, background polynomial function, peak-shape function and lattice parameters. The background was modelled using a Chebyshev polynomial function with 20 terms, while the peak shapes were described with a pseudo-Voigt function. The structures were solved using a simulated annealing algorithm in which molecules were placed at arbitrary positions in the unit cell and allowed to move in random steps. After each step, the R_{wp} parameter was calculated, the steps leading to a decrease in R_{wp} were automatically accepted, while the steps that increase that value were only accepted with a certain gradually decreasing probability. This strategy should locate the global minimum structure, however, the number of steps required to achieve this depends on the number of degrees of freedom. It is, therefore, common practice to constrain parameters of the background and peak shape functions as well as the lattice parameters to the values found in the Pawley refinement procedure. The only remaining variable parameters are the positions and orientations of molecules in the unit cell. In the case of the flsL:dioxane hemisolvate structure the arbitrariness in the choice of origin for the *P1* space group allowed us to constrain the translational degrees of freedom for one of flsL molecules, thus reducing the total number of degrees of freedom to 15 (6 translations and 9 rotations). Once the structure had been found it was subjected to a PLATON[7] symmetry check which revealed the presence of an inversion center. The crystal structure was therefore transformed into the space group *P−1*.. .. . The asymmetric unit of the acetone solvate form II contained one molecule of flsL and one molecule of acetone. Finally, Rietveld refinement[8] was performed in which all of the previously constrained parameters were allowed to vary. For the flsL:doxane hemisolvate, preferred orientation correction with 4th order spherical harmonics was applied.[9]

In addition to the two structures mentioned above, powder diffraction was used to elucidate sthe crystal structure of flsZ. In this case, however, full structure solution was not performed. Instead, several putative structures were found using the CSP procedure (for details see Section 3). One of these structures was found to match the experimental pattern (see Figures S31-34). The Rietveld refinement of the matching structure was then performed.

Single Crystal X-ray Diffraction

Single X-ray diffraction data was collected using an *Agilent SuperNova (Dual Source)* single crystal X-ray diffractometer equipped with an *Atlas CCD Detector*. The data were collected at 150 K using CuK_α radiation (λ = 1.54184 Å). The data were collected and processed using the *CrysAlisPro* program.[10] Empirical absorption correction was performed using spherical harmonics implemented in the *SCALE3 ABSPACK* scaling algorithm. Structure solution and refinement were accomplished using *SHELXS-97* and *SHELXL-97*, respectively.[11] The structure was solved using direct methods. All non-hydrogen atoms were refined anisotropically, while hydrogen atoms associated with carbon and oxygen atoms were refined isotropically in geometrically constrained positions.

Transmission Electron Microscopy

Transmission electron microscopy analysis was performed at ambient temperature on a Philips CM30 instrument operating at 300 kV, and data were collected on photographic films which were subsequently scanned in order to generate digital images. Samples were supported on holey-carbon films on 300 mesh copper grids held within a double tilt sample holder. The diffraction patterns were used for crystal form identification and indexed by comparison with putative fluorescein structures generated by crystal structure prediction. The positions of reflections in experimental diffraction patterns were measured, converted to *d*-spacings and matched to calculated values for the putative structures (further details of the indexing process are given elsewhere).[12],[13] The experimental diffraction patterns were then compared with simulated diffraction patterns of the appropriate zone axes, generated using CrystalMaker SingleCrystal software, to ensure a match.

^{13}C solid-state NMR spectroscopy

NMR was performed on a Bruker Avance 400 spectrometer operating at 400.1 MHz (^1H) and 100.7 MHz (^{13}C), using a Bruker 4 mm double-resonance probe under magic angle spinning at 12.5 kHz. ^{13}C spectra were referenced to external solid glycine (methylene signal at 43.1 ppm relative to TMS at 0 ppm). Spectra were acquired with ramped cross polarization (2.5 ms at a cross polarization field of 70 kHz) from protons (^1H $\pi/2$ pulse of 2.5 μs, spectral width 40 kHz) and SPINAL64 ^1H broadband decoupling (decoupling field 100 kHz) and a 6 s recycle delay. The spectra were processed using the software ACD NMR Processor Academic edition.[14]

UV/Visible light spectroscopy.

Solid state UV/Vis spectra were collected on an Agilent Cary 50 Bio spectrometer equipped with a Diffuse Reflectance Accessory (DRA). The spectra were collected in the 200-800 nm range with a 1 nm step size. The Kubelka-Munk function, *F(R)*, was calculated from the sample reflectance:

$$F(R) = \frac{(1-R)^2}{2R}$$

and the Tauc plot of $(F(R) \times h\nu)^{1/2}$ vs $h\nu$ was constructed. The band gap of the material was then determined by extrapolating the linear region of the plot to the abscissa.

Thermal methods

DSC thermograms were recorded on a Mettler-Toledo 822e instrument. A sample of the material (3-5 mg) was placed in a 40μl aluminium crucible with a hole in the lid. The samples were heated from 25 to 400 °C at a rate of 10 °C/min.
TGA curves were recorded on a Mettler-ToledoTGA STARe 1 instrument. Approx. 10 mg of material was placed in a 100μl aluminium crucible and heated from 30 to 400 °C at a rate of 10 °C/min.

Cocrystal behavior under high and low humidity conditions.

Samples of grey flsL:pyrazine cocrystal were placed in a 0% RH chamber (desiccator filled with phosphorus pentoxide) and a 98% RH chamber (desiccator filled with saturated aqueous solution of potassium sulphate). The sample stored under high humidity began to show yellow coloration within 30 minutes from the start of the experiment and became brightly colored within 2 hours. The sample stored under a dry atmosphere displayed only slight coloration after 24 hours (Figure S1). At the end of both experiments the PXRD patterns of cocrystal samples were collected to verify that the bulk crystal structure had not changed.

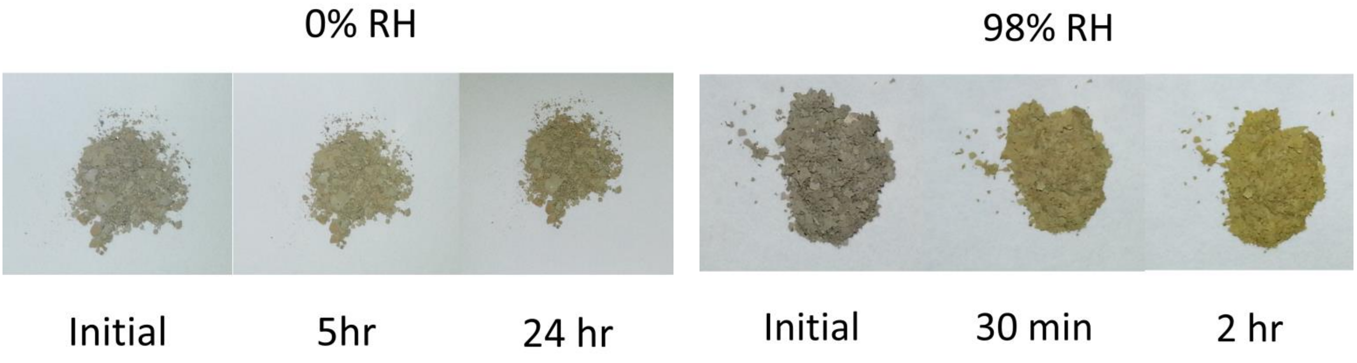

Figure S1. The evolution of color of flsL:pyrazine cocrystal stored under dry atmosphere (left) and high humidity (right) conditions.

3. Computational studies

Crystal Structure Prediction of zwitterionic and lactoid fluorescein (flsZ and flsL)

Molecular geometry optimization of flsZ and flsL in three conformations differing by the orientation of OH groups (Figure 4) was performed at the B3LYP/6-311G(d,p) level of theory[15] using the program Gaussian03.[16] Initial structure searches were performed in the 20 most common space groups (ΠP1, P-1, $P2_1$, $P2_1/c$, $P2_12_12$, $P2_12_12_1$, $Pna2_1$, $Pca2_1$, Pbca, Pbcn, C2/c, Cc, C2, Pc, P2/c, Fdd2, Pccn, $I4_1/a$, $P4_1$ and R-3, , all Z'=1) using the program CrystalPredictor.[17] Intermolecular interactions were modelled using a pairwise repulsion-dispersion W99 potential[18],[19],[20] and atomic charges fitted to reproduce the molecular electrostatic potential at the Van der Waals surface.[21] The crystal structures thus obtained were optimized using an improved electrostatic model based on Distributed Multipole Analysis (DMA)[22] in the program DMACRYS 2.0.4,[23] including multipoles up to hexadecapole on all atoms. The lowest energy predicted structures of flsL (up to 15 kJ mol^{-1} above the global minimum) were further optimized by treating the flexibility of the OH groups in the program CrystalOptimizer.[24] The effect of molecular polarization was modelled by calculating the multipoles within the Polarized Continuum Model (PCM)[25],[26],[27] with dielectric permittivity set to ε=3.0.[28] The final set of lowest energy structures (up to 15 kJ mol^{-1} above the global minimum) was energy-minimized using plane-wave DFT calculations in the program CASTEP 6.1.[29]

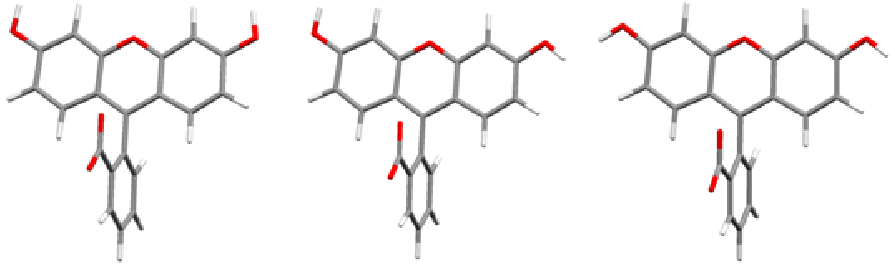

Figure S2. Three conformations of flsZ used for CSP structure generation. The conformations of flsL were generated in a similar way.

Plane-wave DFT calculations

All calculations were performed in the program CASTEP 6.1.[29] Unless stated otherwise, the calculations were performed with the PBE[30] functional with norm-conserving pseudopotentials.[31] The plane-wave cut-off was set to 700 eV and Monkhorst-Pack[32] k-point spacing was set to 0.03 Å^{-1}. Convergence testing was performed with respect to the total energy and atomic forces.

Geometry optimization

All structures were geometry-optimized prior to the property calculations. During the optimization both atomic positions and unit cell parameters were allowed to vary. Dispersion forces, which are known to be very important for the accurate determination of the unit cell volumes of organic crystals, were modelled using the G06 semi-empirical dispersion correction scheme. [33],[34] The convergence criteria were set as follows: energy change 10^{-5} eV/atom, maximum force 10^{-2} eV Å^{-1}, maximum atom displacement 10^{-3} Å, residual stress 0.05 GPa. All other parameters were left at their default values.

Optical properties

Optical reflectance and absorption spectra as well as the density of states (DOS) were computed for all materials using the program OptaDOS.[35],[36] In addition partial density of states (PDOS) showing the contribution of fluorescein molecules was computed for the cocrystals and solvates of flsL. All the calculations were performed for the polycrystalline geometry, DOS was sampled at 0.001 eV intervals and the adaptive broadening[37] scheme was used with the smearing parameter set to 1A. Convergence of the calculated band gap with respect to Brillouin zone sampling was tested and the k-point spacing of 0.015 Å^{-1} was found to be sufficient.

NMR calculations

NMR shielding parameters were calculated using the GIPAW[38] method and pseudopotentials generated on the fly.[39] For the NMR calculations a higher plane-wave cut-off of 1000 eV was used and the scale of the fine g-vector grid was set to 3.
In order to make comparison between calculation and experiment, the calculated shieldings need to be converted into chemical shifts. The isotropic part of the shielding tensor is related to the experimentally measured chemical shift via the following equation:

$$\delta_{iso} = -a\sigma_{iso} + \sigma_{ref}$$

where parameter *a* should theoretically be equal to unity and the value of σ_{ref} corresponds to the chemical shielding of the NMR standard (TMS for ^{13}C). In practice the values of *a* and σ_{ref} are obtained by fitting the calculated shieldings to the experimentally measured chemical shifts. The resulting regression equation can then be used to calculate the chemical shifts for systems similar to those used in the calibration set. In the present study the spectra of the three solvates of flsL were used for calibration, Naturally, the regression analysis relies on the assignment of experimental chemical shifts to their corresponding atoms. The assignment process, however, is non-trivial, since many aromatic carbon atoms have very similar chmical shifts making it difficult to distinguish between them. The approach we have taken was the following: whenever a group of atoms had similar chemical shifts, the calculated shifts were assigned in such a way that gives the best numerical fit, i. e. the biggest chemical shift is matched with the smallest calculated shielding, etc. In the cases where individual shifts could not be extracted due to peak overlap, the corresponding chemical shieldngs were averaged and the average value was matched with the experimental peak position.The following regression equation was obtained:

$$\delta_{iso} = -0.9526\sigma_{iso} + 163.66$$

The resulting equation is in good agreement with the previously reported values.[40] This equation was used to model the spectra of red and yellow fluorescein.

4. Crystallographic data

Crystallographic parameters of the newly-determined crystal structures are summarized in Table S1. Figures S3-S8 show the Pawley and Rietveld fits for the three crystal structures determined from powder data. Close similarity between the corresponding Pawley and Rietveld fits gives an indication for the correctness of these structures. The Rietveld fits for the previously reported crystal forms of fluorescein are given in Figures S9-S18. Here, Rietveld refinement was used to confirm that the analysed material matches the reported crystal structure. In particular, figures S14-S17 show the Rietveld fits of the flsL:pyrazine cocrystal prepared with different excess amounts of pyrazine and stored under different humidity conditions. Since all these fits were obtained using the same crystal structure (CSD RIGTOS01), it is evident that the colour changes in this material occur solely in the surface layer, without affecting the bulk crystal structure. Finally, Figure S19 shows the similarity in crystal packing of flsL:pyrazine cocrystal and flsL:acetone solvate form II.

Table S1. Crystallographic parameters for the structures determined from single crystal and powder X-ray diffraction.

Structure	flsL:dioxane$_{2.5}$	flsL:dioxane$_{0.5}$	flsL:acetone form II	flsZ
CCDC number	1050814	1050815	1404506	1050816
Chemical formula	$C_{20}H_{12}O_5$ $(C_4H_8O2)_{2.5}$	$C_{20}H_{12}O_5$ $(C_4H_8O2)_{0.5}$	$C_{20}H_{12}O_5$ (C_3H_6O)	$C_{20}H_{12}O_5$
Formula weight	552.56	376.36	390.39	332.31
Crystal system	monoclinic	triclinic	monoclinic	orthorhombic
Space group	$P2_1/n$	P-1	$P2_1/c$	Pbca
a / Å	7.99350(10)	7.8233(2)	7.7629(3)	8.3032(6)
b / Å	20.3407(3)	10.6057(4)	18.9044(6)	12.4072(5)
c / Å	16.7100(2)	11.0170(3)	12.9402(5)	29.716(3)
α / °	90	97.475(2)	90	90
ß / °	101.364(1)	91.940(2)	92.472(2)	90
γ / °	90	107.162(3)	90	90
V / Å^3	2663.67	863.5	1897.24	3061.3
Z	4	2	4	8
T / K	150	293	293	293
X-ray radiation type	Cu Kα	Cu Kα_1	Cu Kα_1	Cu Kα_1
R_1 (I>2σ), wR^2(I>2σ)	3.05%, 3.45%	-	-	-
R_{wp}, R_{Bragg}	-	4.0%, 1.0%	3.9%, 1.3%	3.1%, 0.7%
Goodness of fit	1.023	1.396	1.957	1.489

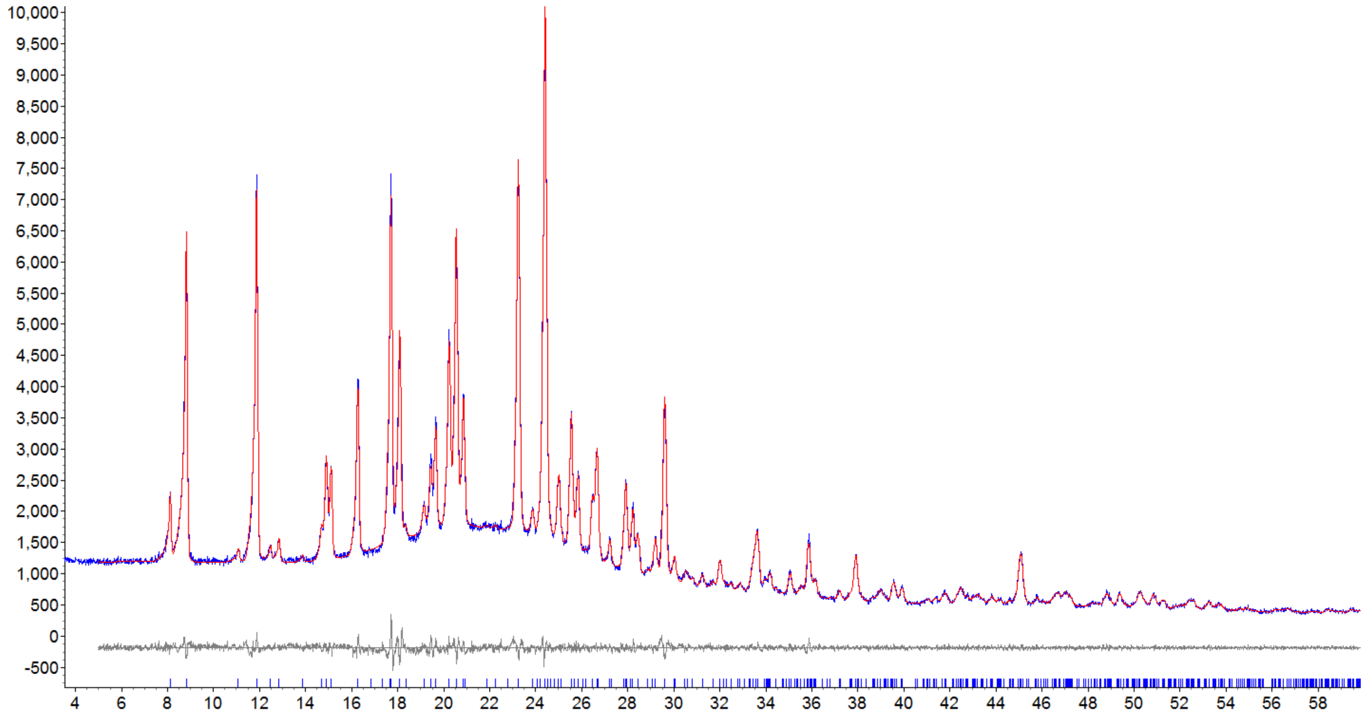

Figure S3. Plot of the Pawley refinement for flsL:dioxane hemisolvate (blue: experimental profile, red : calculated profile, grey: difference).

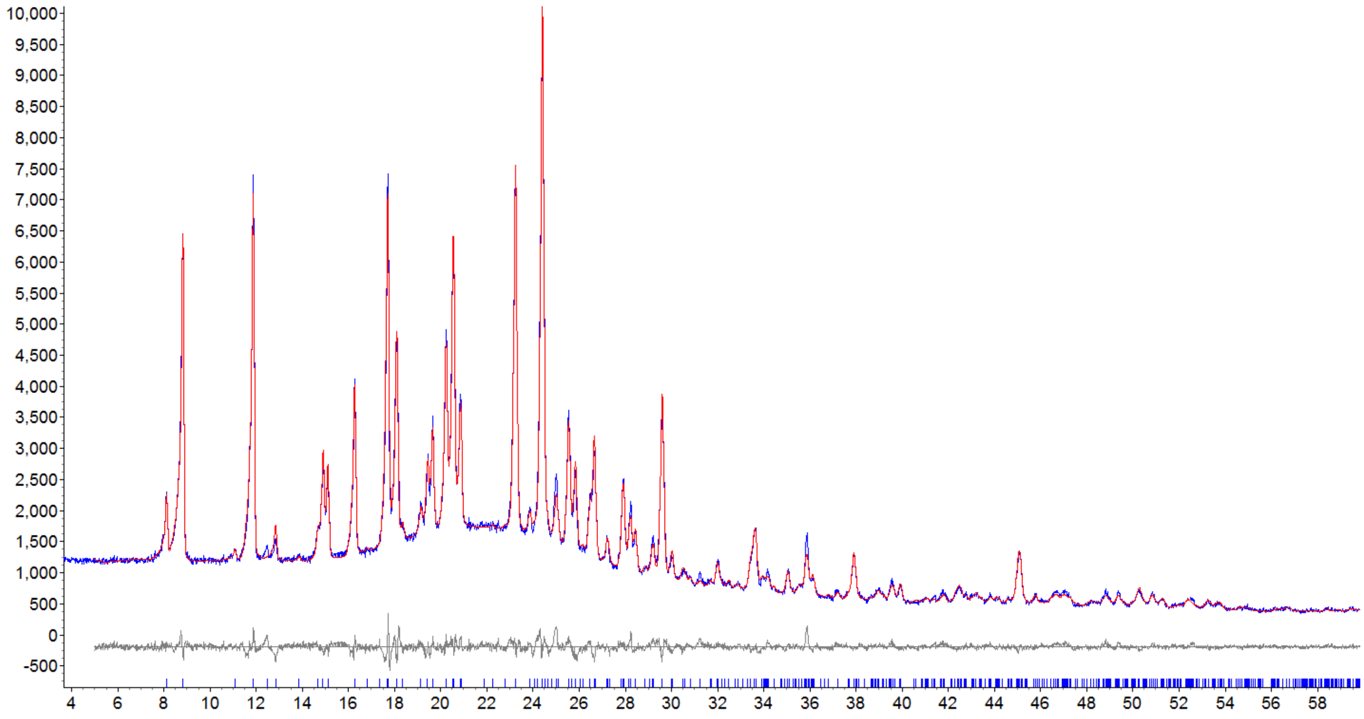

Figure S4. Plot of the Rietveld refinement for flsL:dioxane hemisolvate (blue: experimental profile, red : calculated profile, grey: difference). Close similarity between the Rietveld and Pawley fit (Figure S3) suggests that the structure has been refined sufficiently well.

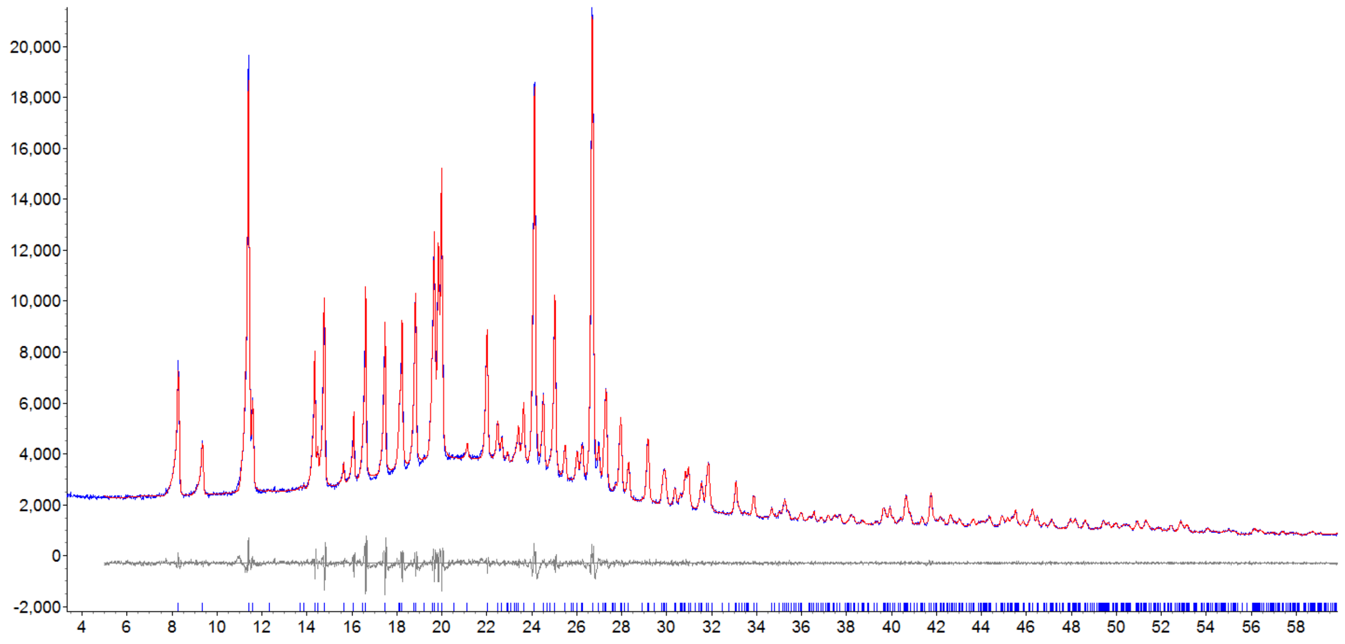

Figure S5. Plot of the Pawley refinement for flsL:acetone monosolvate form II (blue: experimental profile, red : calculated profile, grey: difference).

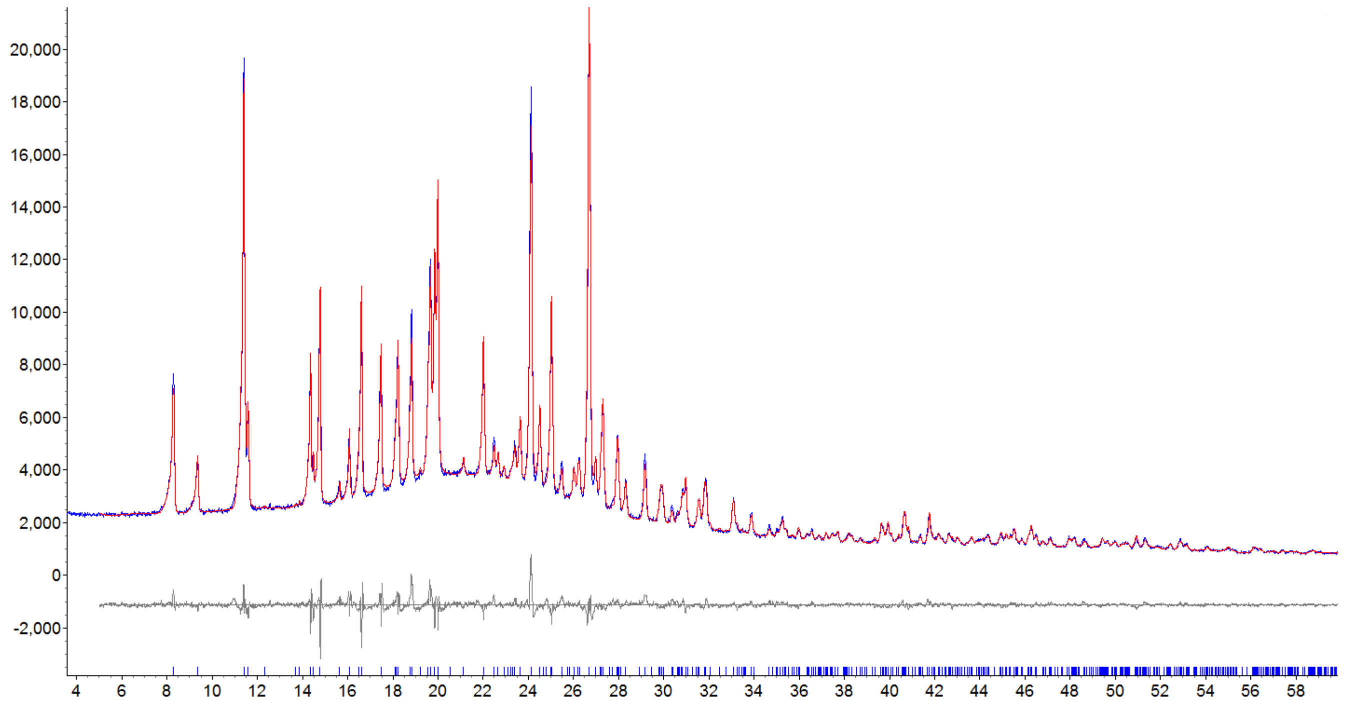

Figure S6. Plot of the Rietveld refinement for flsL:acetone monosolvate form II (blue: experimental profile, red : calculated profile, grey: difference). Close similarity between the Rietveld and Pawley fit (Figure S5) suggests that the structure has been refined sufficiently well.

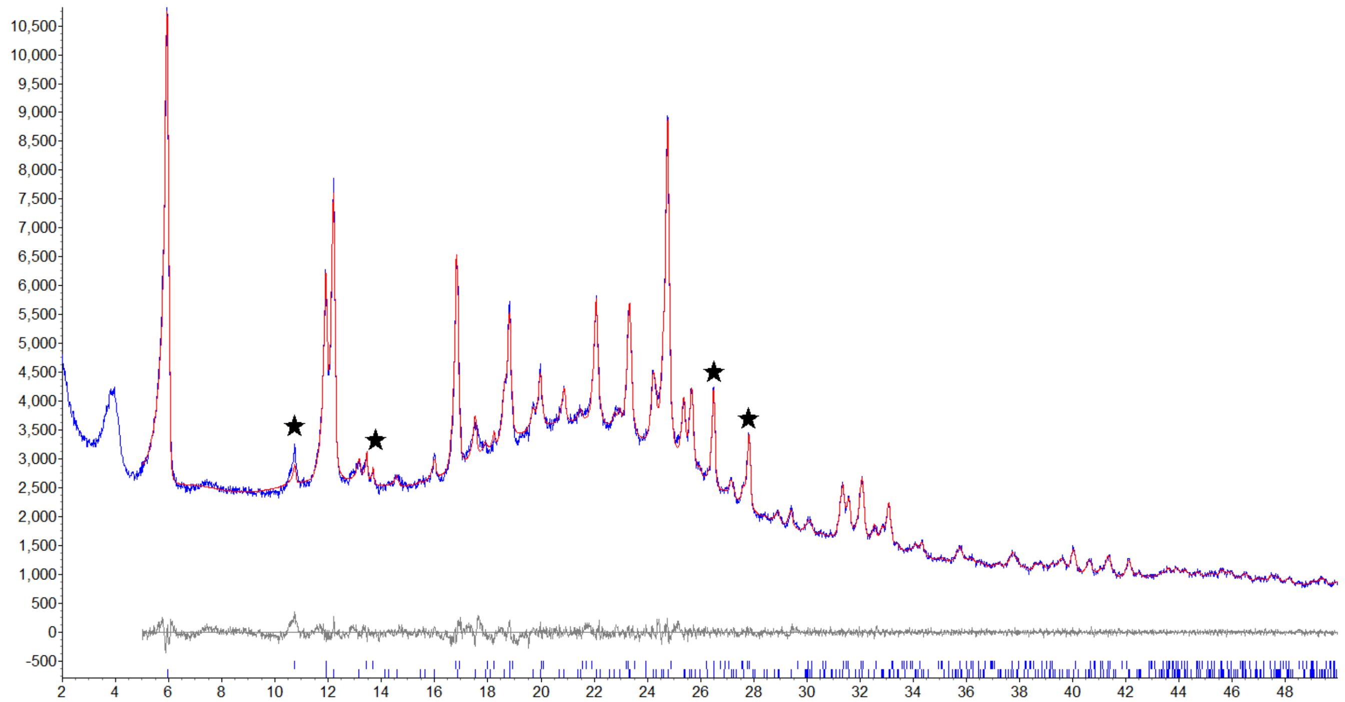

Figure S7. Plot of the Pawley refinement for flsZ (blue: experimental profile, red : calculated profile, grey: difference). The material contains an impurity of flsQ (peaks are marked with a star symbol). In order to model the impurity peaks, the reported crystal structure of flsQ (CSD NUKCOL) was used in the refinement. For more details see Figure S8.

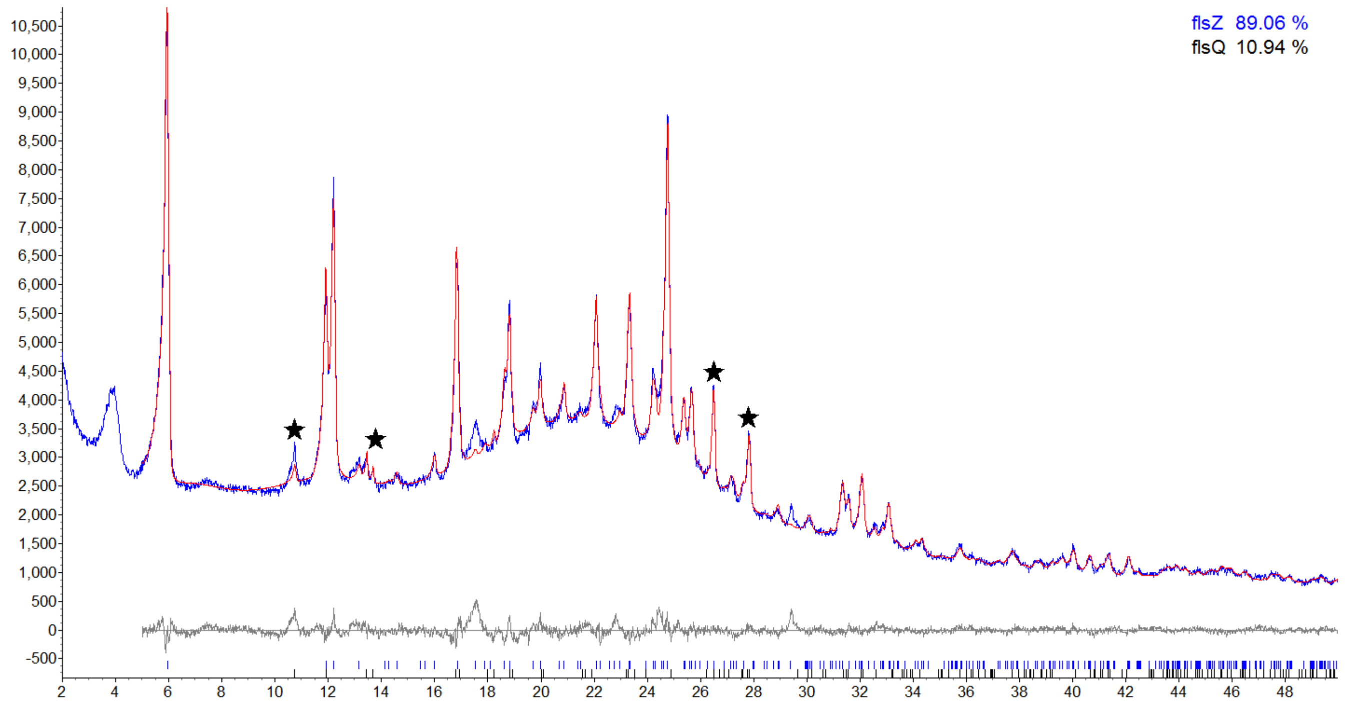

Figure S8. Rietveld refinement for flsZ (blue: experimental profile, red: calculated profile, grey: difference). Refinement has shown the presence of 11% flsQ impurity in the material (peaks are marked with a star symbol). Close similarity between the Rietveld and Pawley fit (Figure S7) suggests that the structure has been refined sufficiently well for the given data quality. Please note: the peaks at 3.9° and 7.5° 2θ could not be fitted with neither flsZ nor flsQ crystal structures. The 3.9° peak is noticeably broader than any other peaks in the powder pattern, suggesting presence of a low-crystalline impurity.

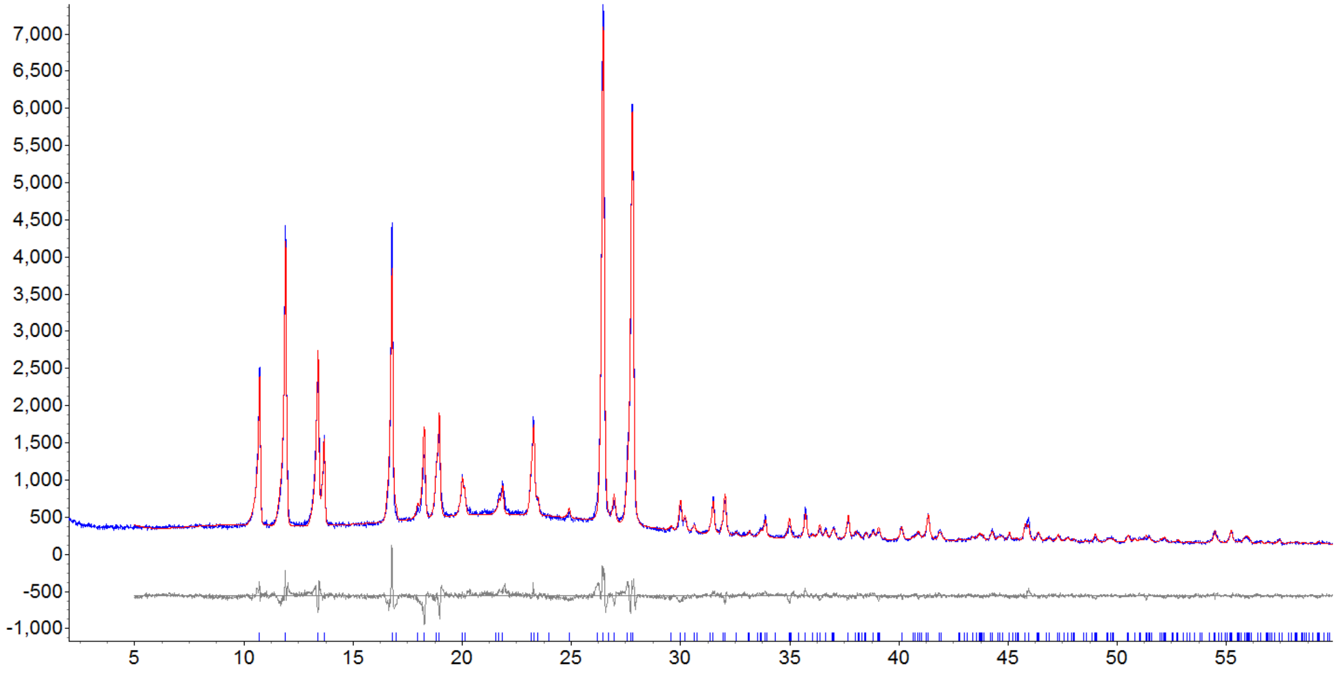

Figure S9. Rietveld refinement of the crystal structure of flsQ (CSD NUKCOL) against the powder pattern of red fluorescein.

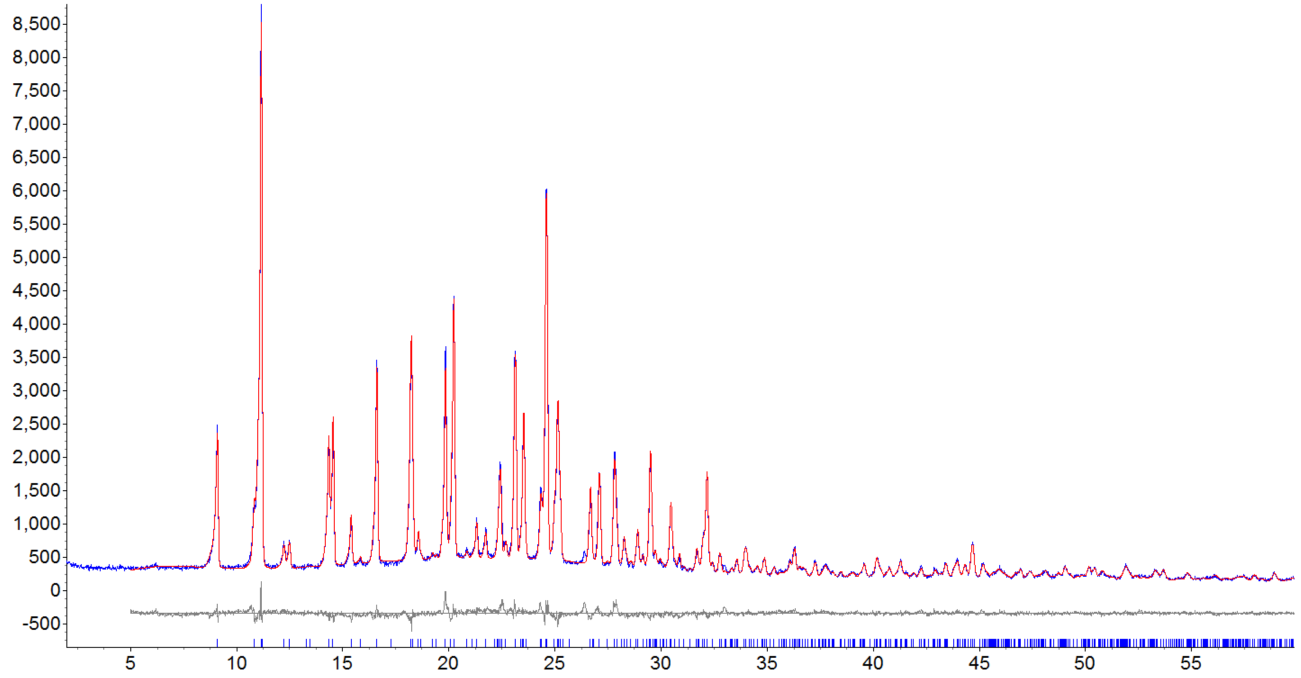

Figure S10. Rietveld refinement of the crystal structure of flsL:acetone monosolvate form I(CSD FLSCAC).

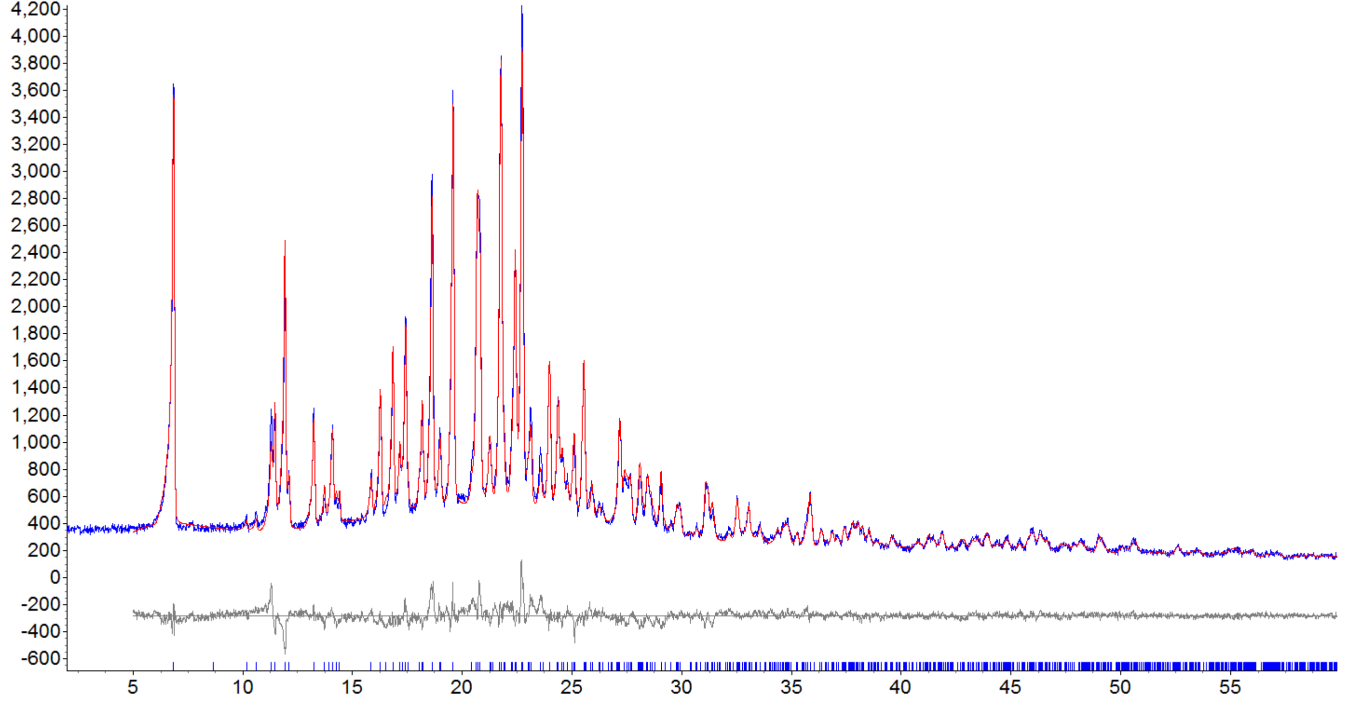

Figure S11. Rietveld refinement of the crystal structure of flsL:dioxane hemipentasolvate.

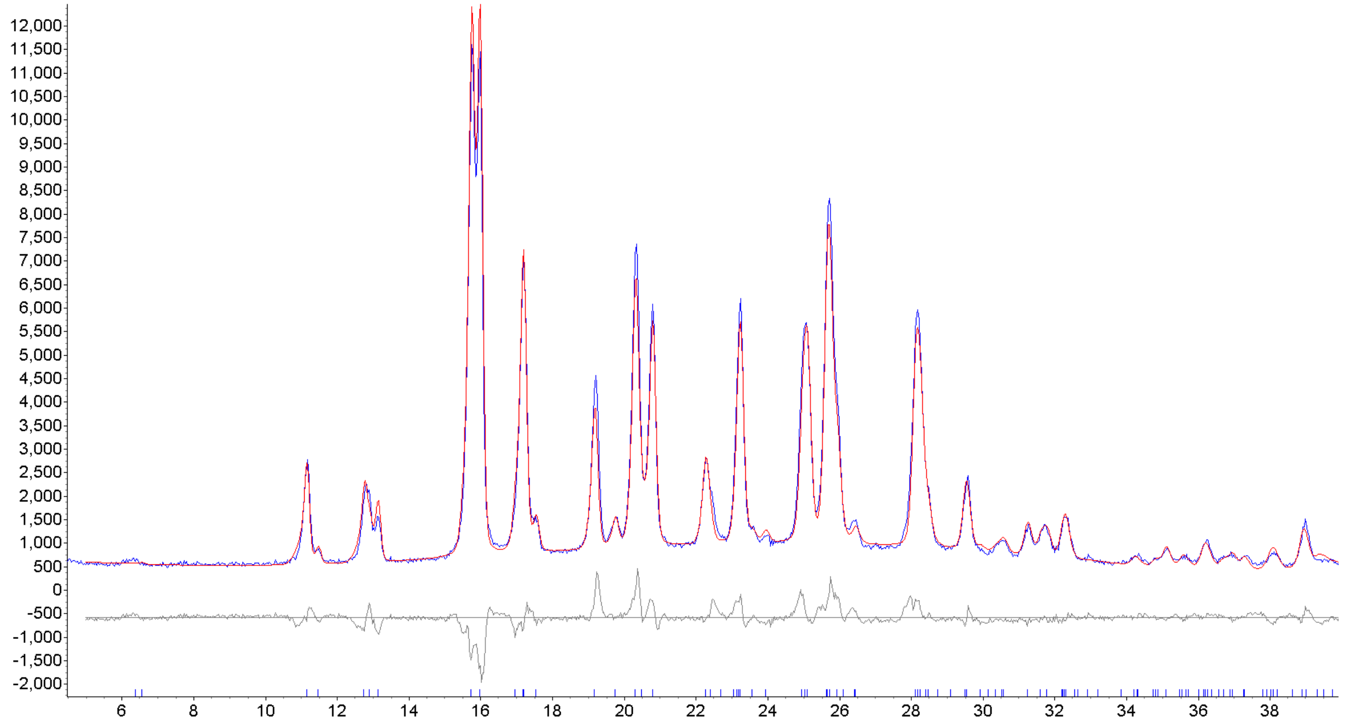

Figure S12. Rietveld refinement of the crystal structure of the flsL:phenanthridine cocrystal (CSD RIGTIM01).

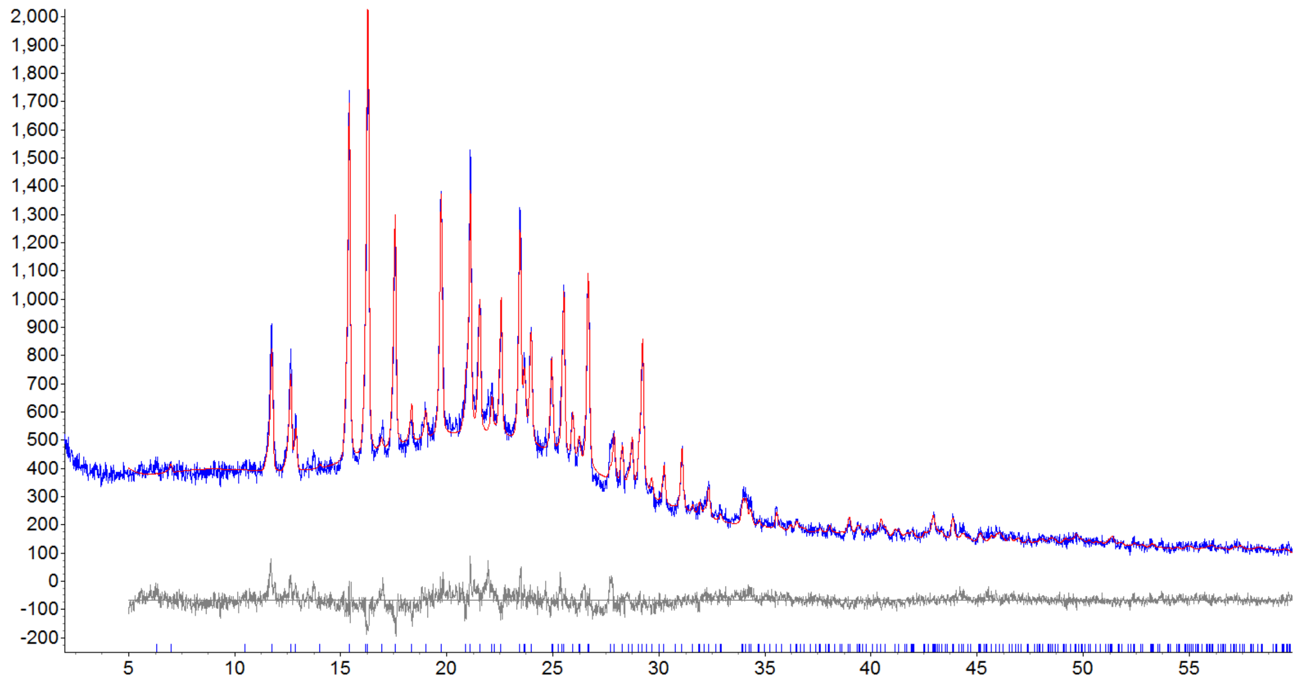

Figure S13. Rietveld refinement of the crystal structure of the flsL:acridine cocrystal (CSD RIGTOS01).

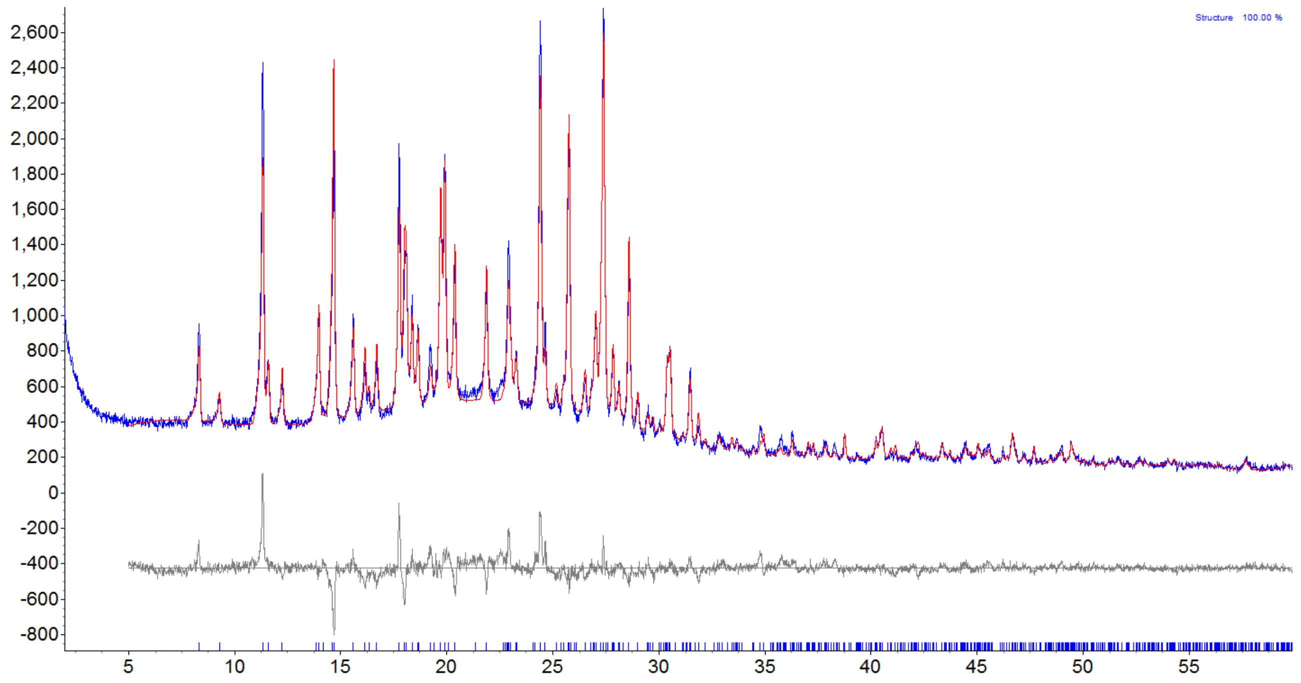

Figure S14. Rietveld refinement of the crystal structure of the flsL:pyrazine cocrystal (CSD RIGTUY) coated with a yellow flsZ surface layer.

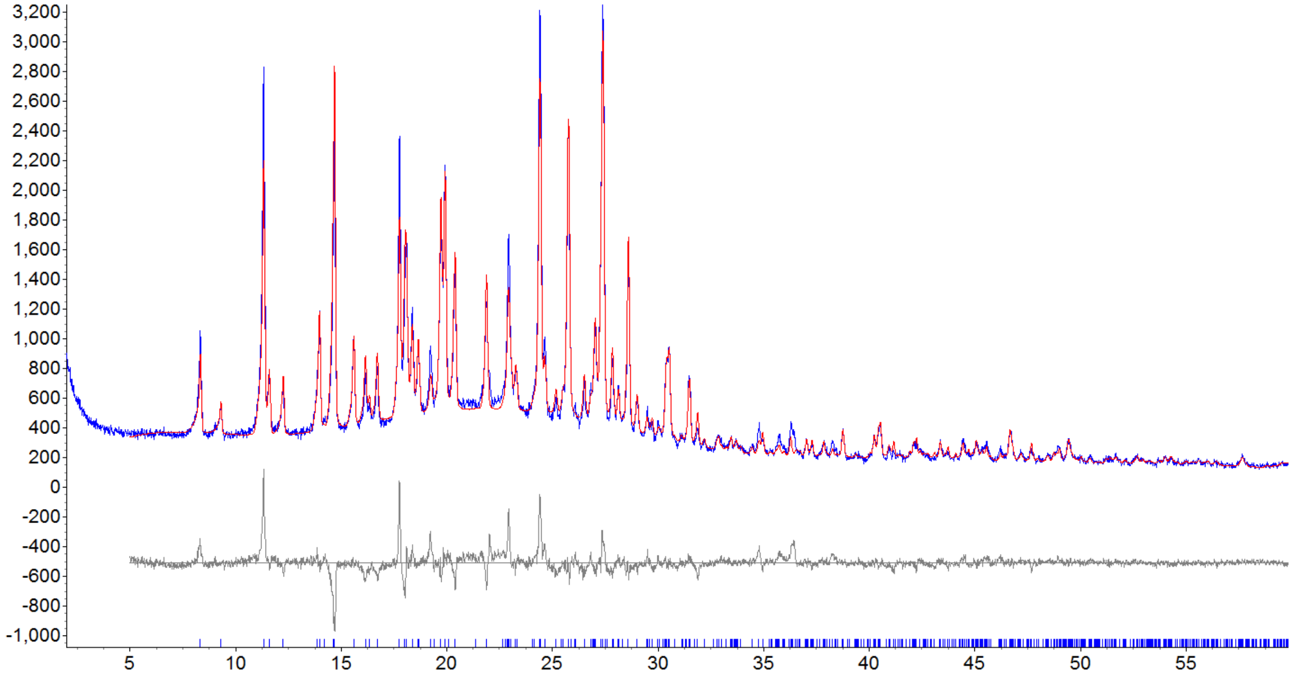

Figure S15. Rietveld refinement of the crystal structure of flsL:pyrazine (CSD RIGTUY) in the grey form (no flsZ surface coating). Comparison with figure S14 shows that the coated and uncoated materials have identical bulk crystal structures.

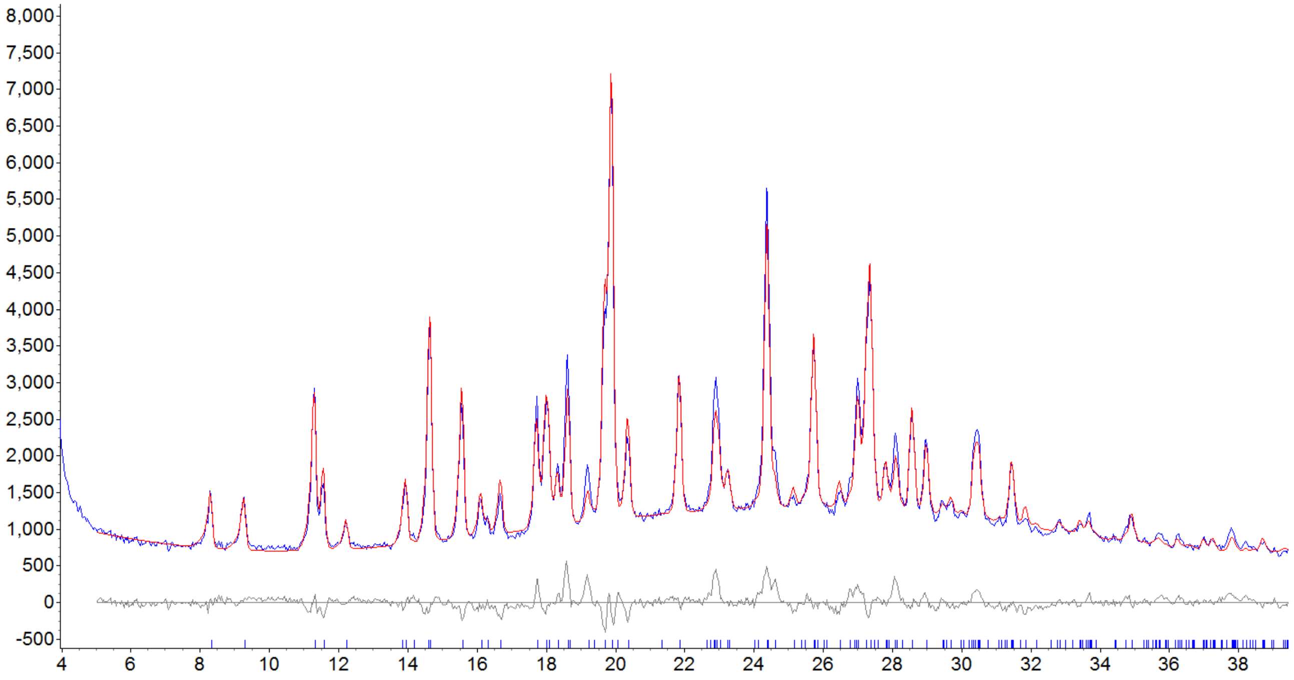

Figure S16. Rietveld refinement of the grey flsL:pyrazine cocrystal aged at 98% RH for two hours. During the ageing process the material has undergone a color change due to formation of a zwitterion surface layer. The powder pattern, however, indicates that the bulk crystal structure has not changed in the process.

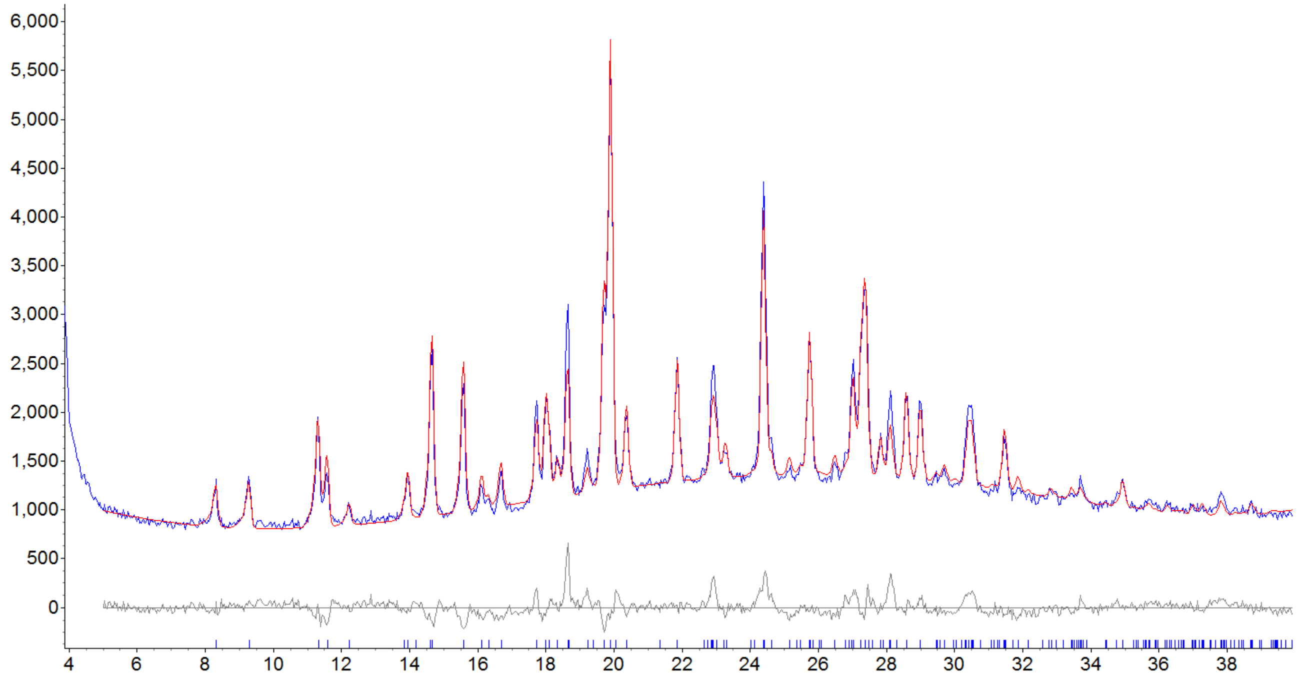

Figure S17. Rietveld refinement of the grey flsL:pyrazine cocrystal aged at 0% RH for 72 hours. During the ageing process the color of the material and the bulk crystal structure have not changed.

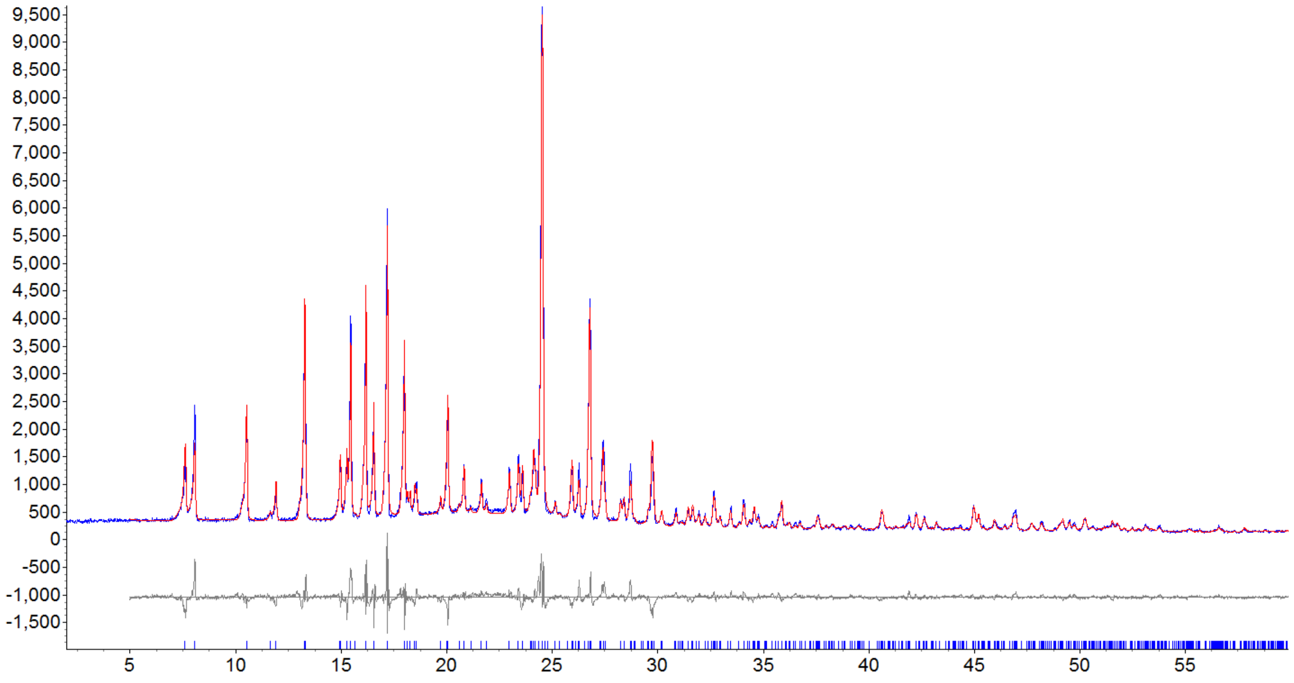

Figure S18. Rietveld refinement of diacetylfluorescein (CSD SIQJIL).

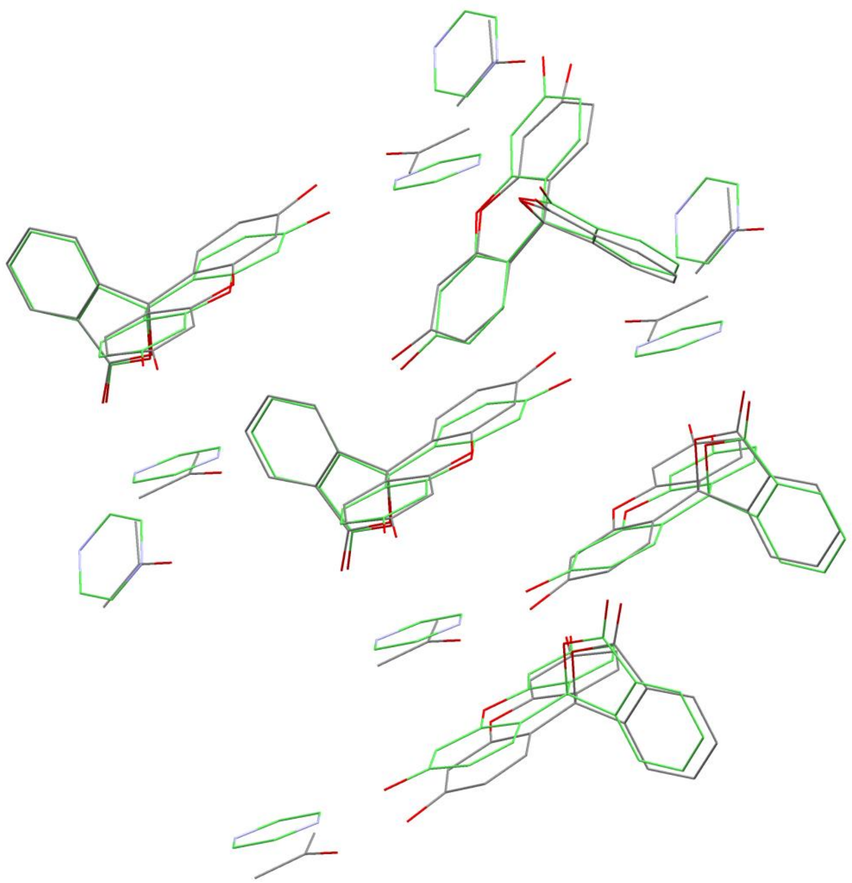

Figure S19. Overlay of the flsL:acetone (form II) and flsL:pyrazine crystal structures.

5. Thermal analyses

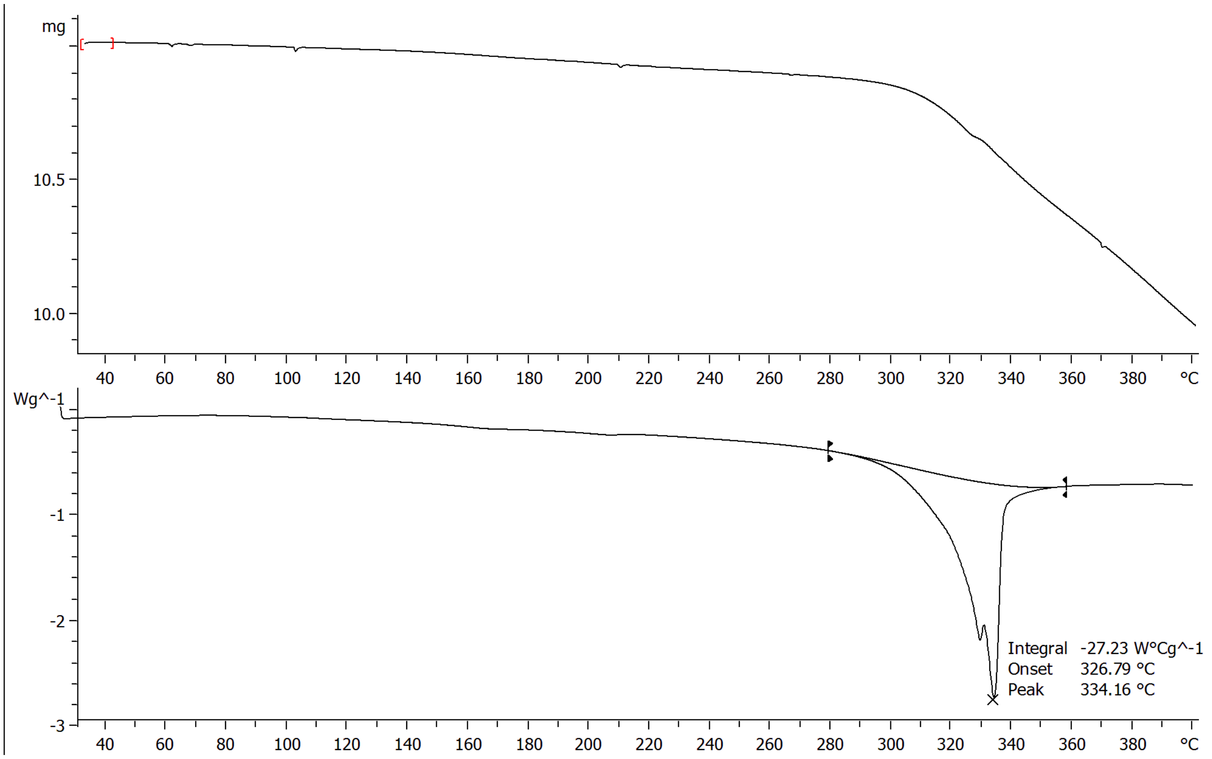

Figure S20. TGA (top) and DSC (bottom) curves of red fluorescein (flsQ tautomer). The material melts and simultaneously degrades above 326.8 °C (onset

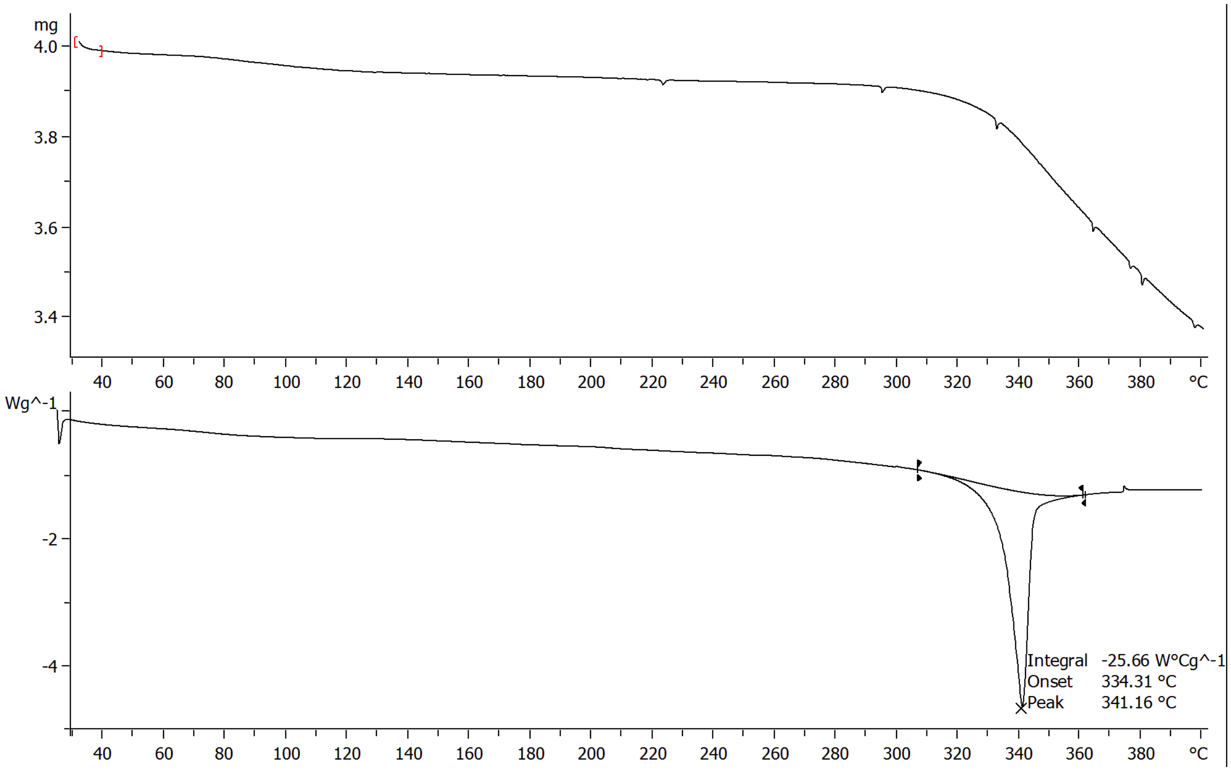

temperature).

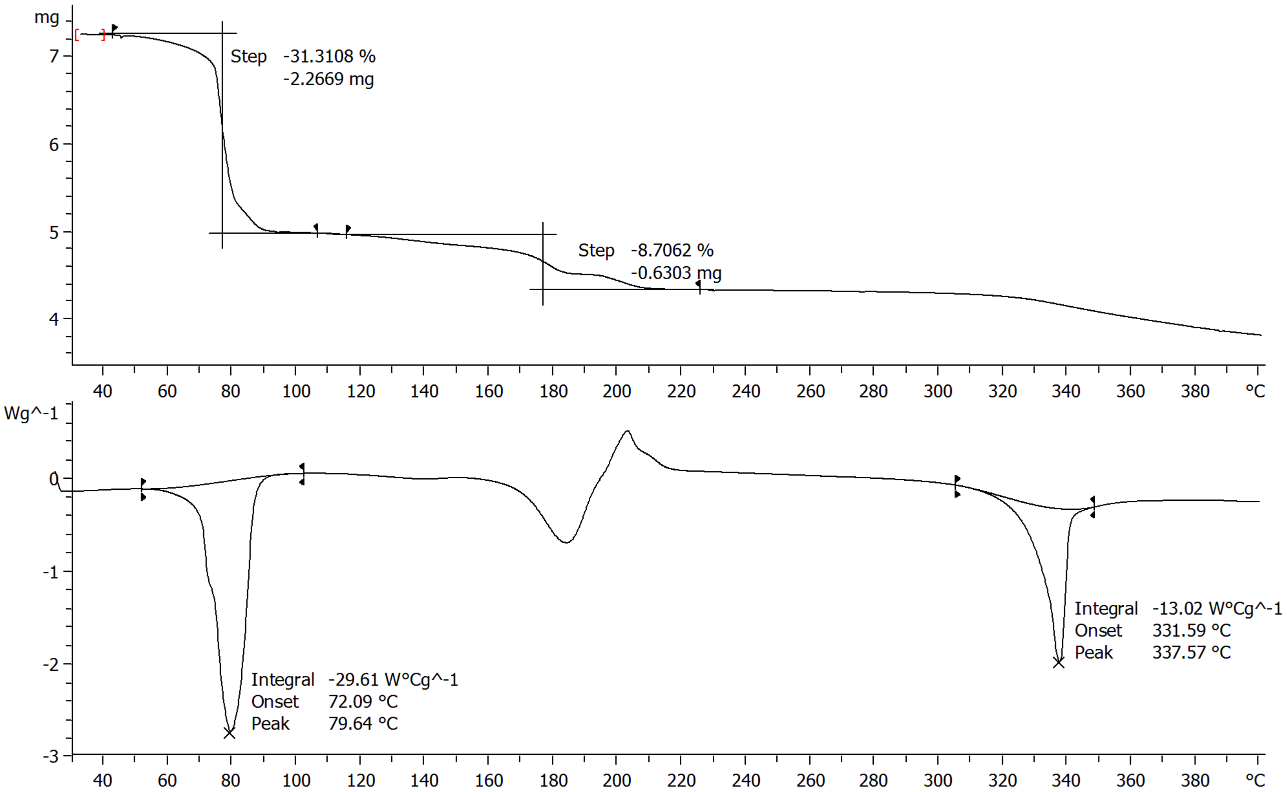

Figure S21. TGA (top) and DSC (bottom) curves of yellow fluorescein (flsZ tautomer). During heating the material converts into red form (flsQ), however the enthalpy of the process is so small that the transition is not observed in the DSC experiment. The material melts and simultaneously degrades above 334.3 °C (onset temperature).

Figure S22. TGA and DSC curves of flsL:dioxane hemipentasolvate. The weight loss of 31.3% (31.9% theoretical) corresponds to the formation of flsL:dioxane hemisolvate. At 180°C the remaining dioxane is lost (8.7% experimental, 8.0% theoretical). On the DSC curve this event is shown as an endothermic peak (desolvation to lead unsolvated flsL) followed by an exothermic peak at 210°C (most likely flsL→flsQ transformation). The endothermic event at 331.6°C (onset) is the melting and degradation of flsQ.

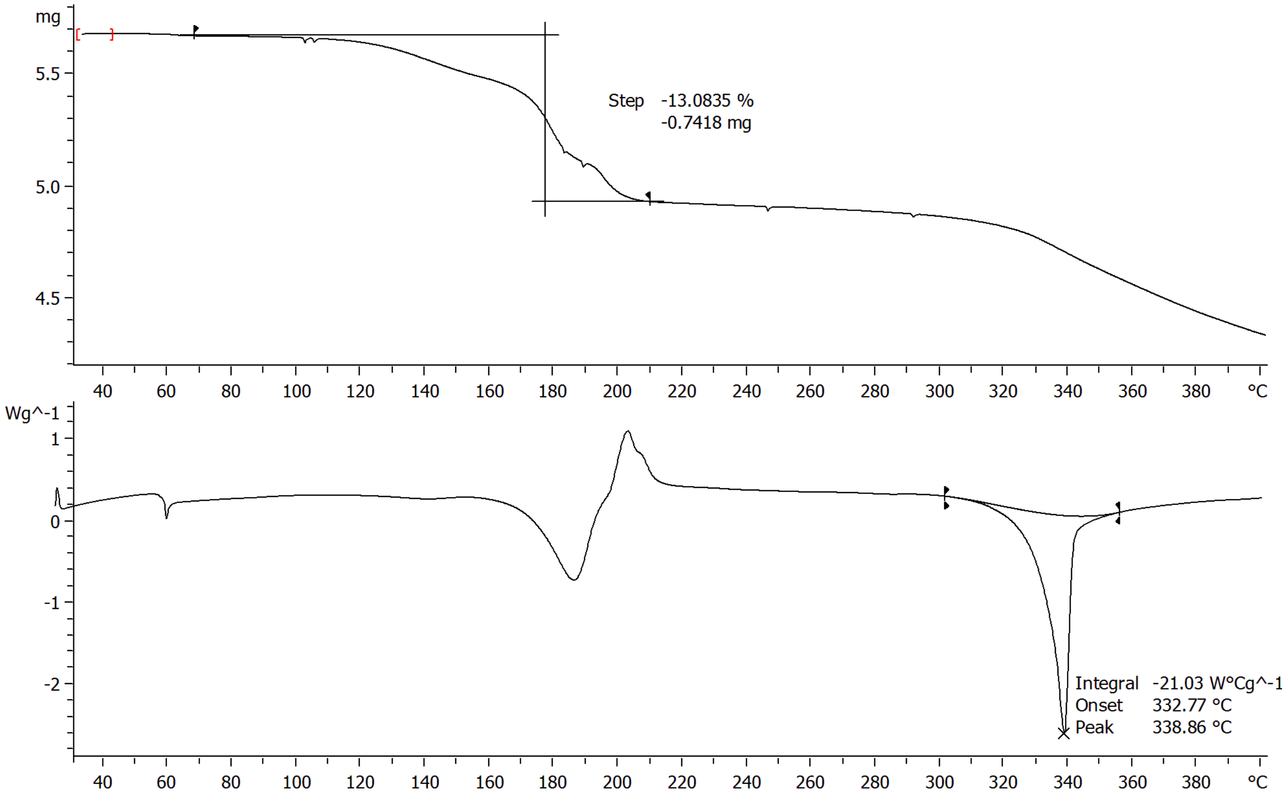

Figure S23. TGA and DSC curves of flsL:dioxane hemisolvate. TGA weight loss of 13.1% (theoretical 11.7%) corresponds to the complete desolvation of the material. DSC events match those in the curve of flsL:dioxane hemipentasolvate.

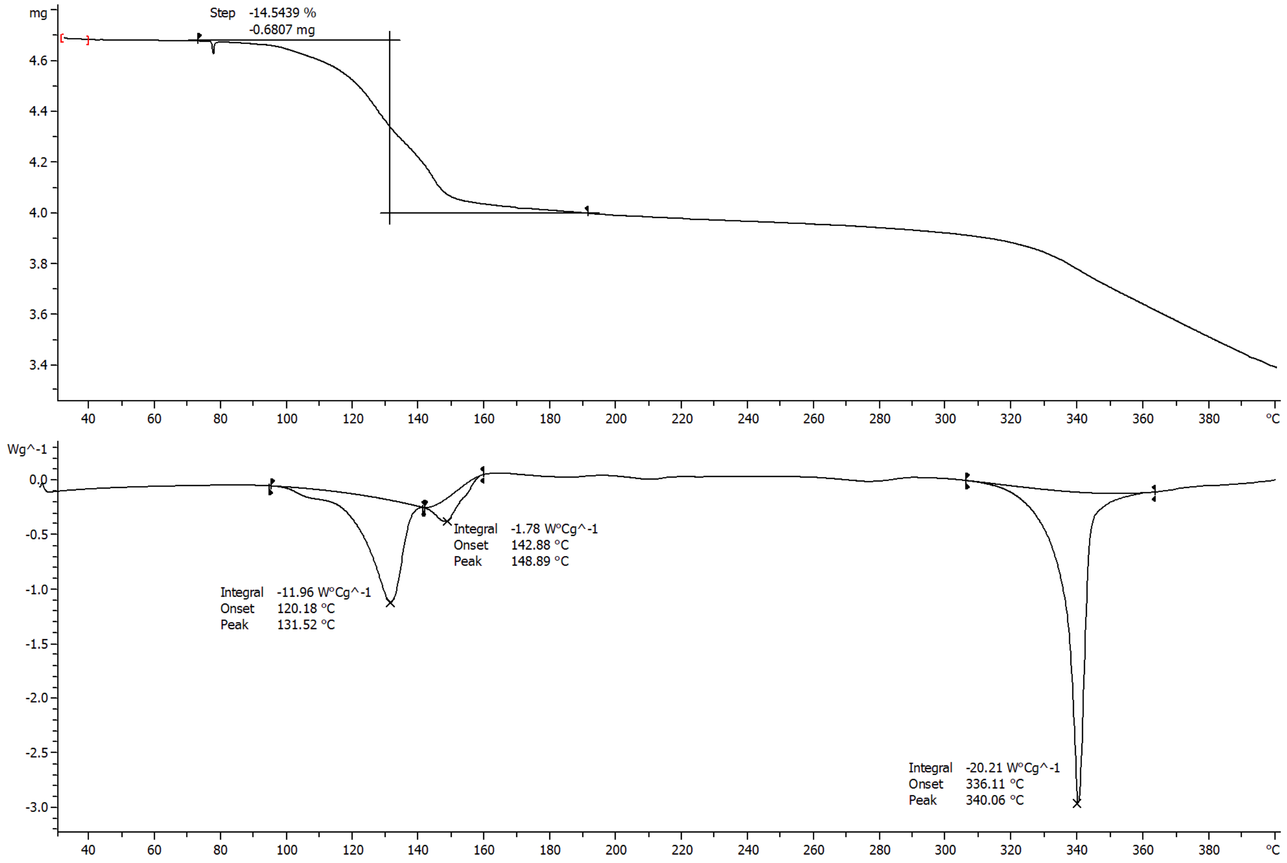

Figure S24. TGA and DSC curves of flsL:acetone monosolvate form I. TGA weight loss of 14.5% (theoretical 14.9%) corresponds to the

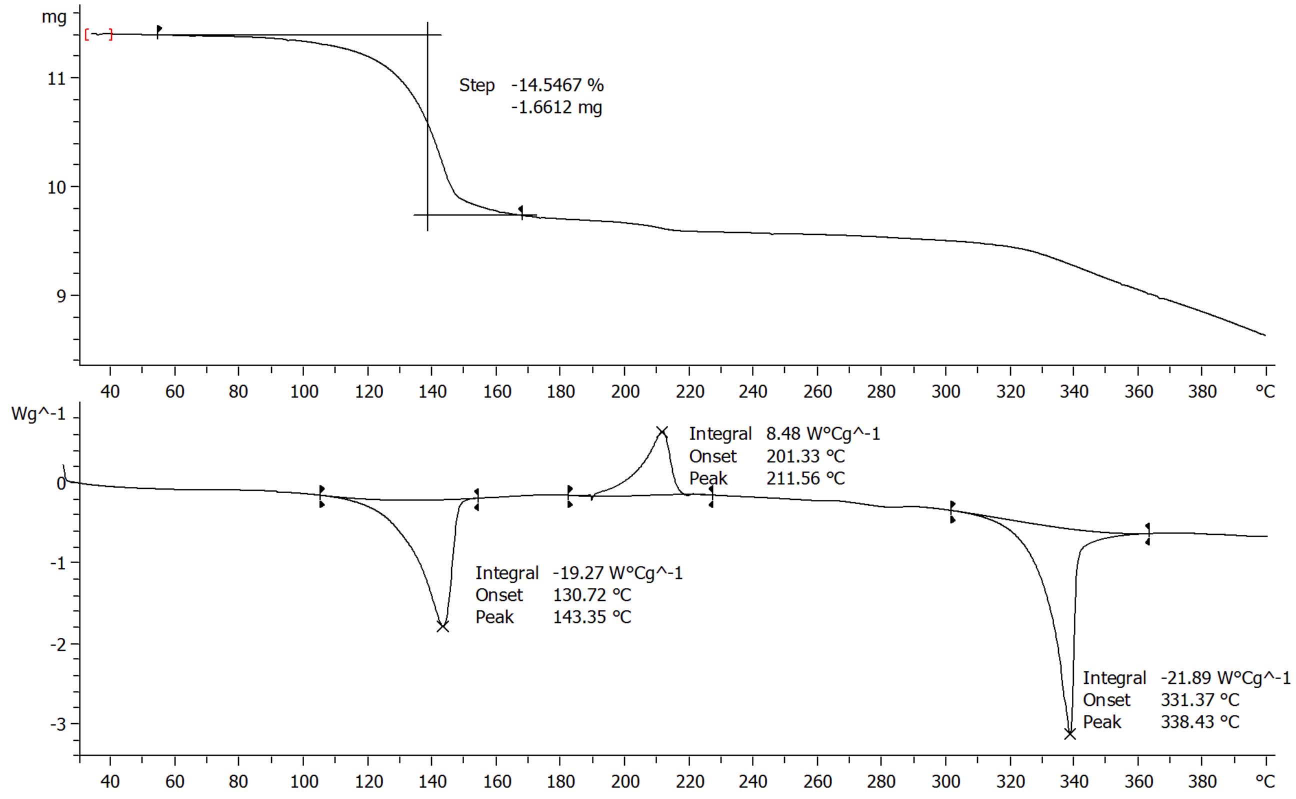

complete desolvation of the material. From the DSC curve it is evident that desolvation occurs in two steps.

Figure S25. TGA and DSC curves of flsL:acetone monosolvate form II. Desolvation occurs at 140°C (experimental weight loss 14.5%, theoretical - 14.9%). DSC shows and endothermic desolvation event at 143 °C followed by exothermic formation of flsQ above 211 °C and melting and decomposition of flsQ starting at 331.4 °C.

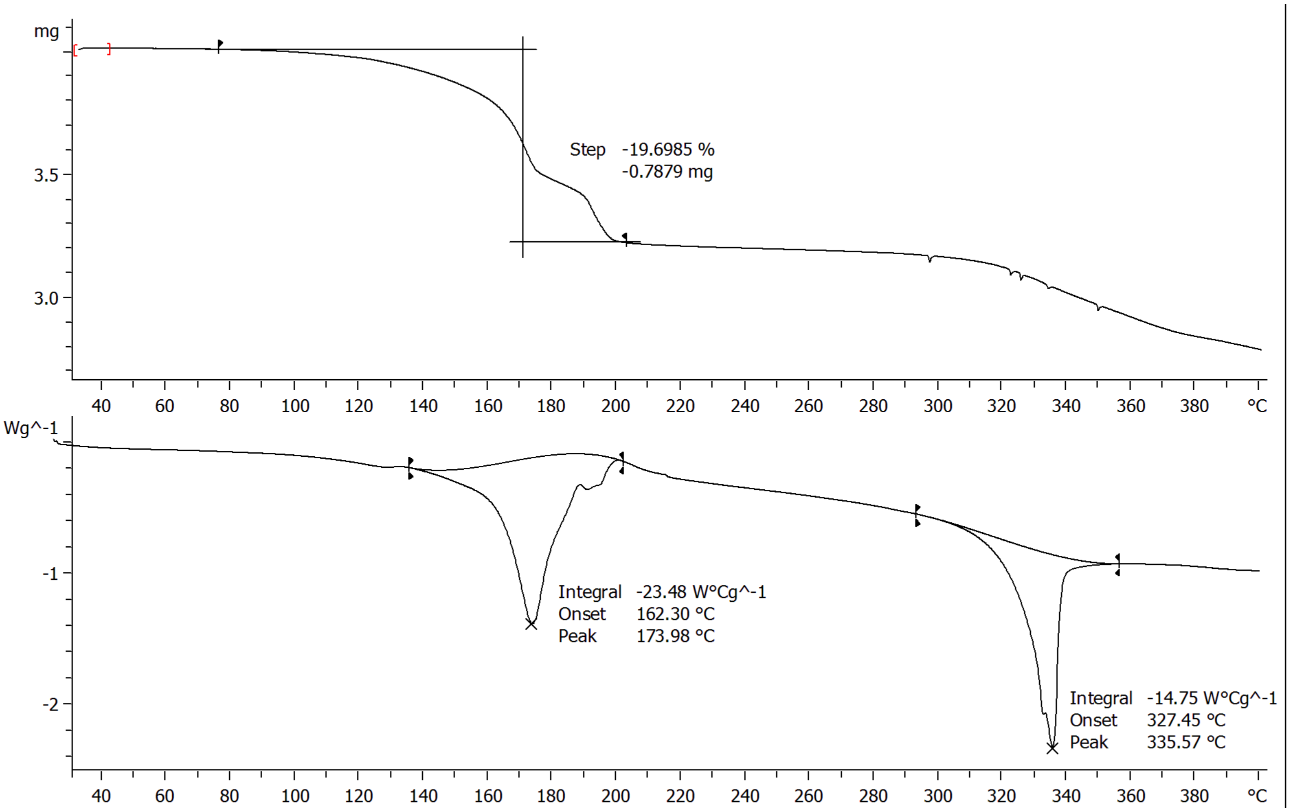

Figure S26. TGA and DSC curves of flsL:pyrazine cocrystal. The cocrystal dissociates with evaporation of pyrazine at 170 °C (experimental weight loss 19.7%, theoretical – 19.4%). DSC trace shows no exothermic events after desolvation which suggests that the cocrystal dissociates straight into pyrazine and flsQ without

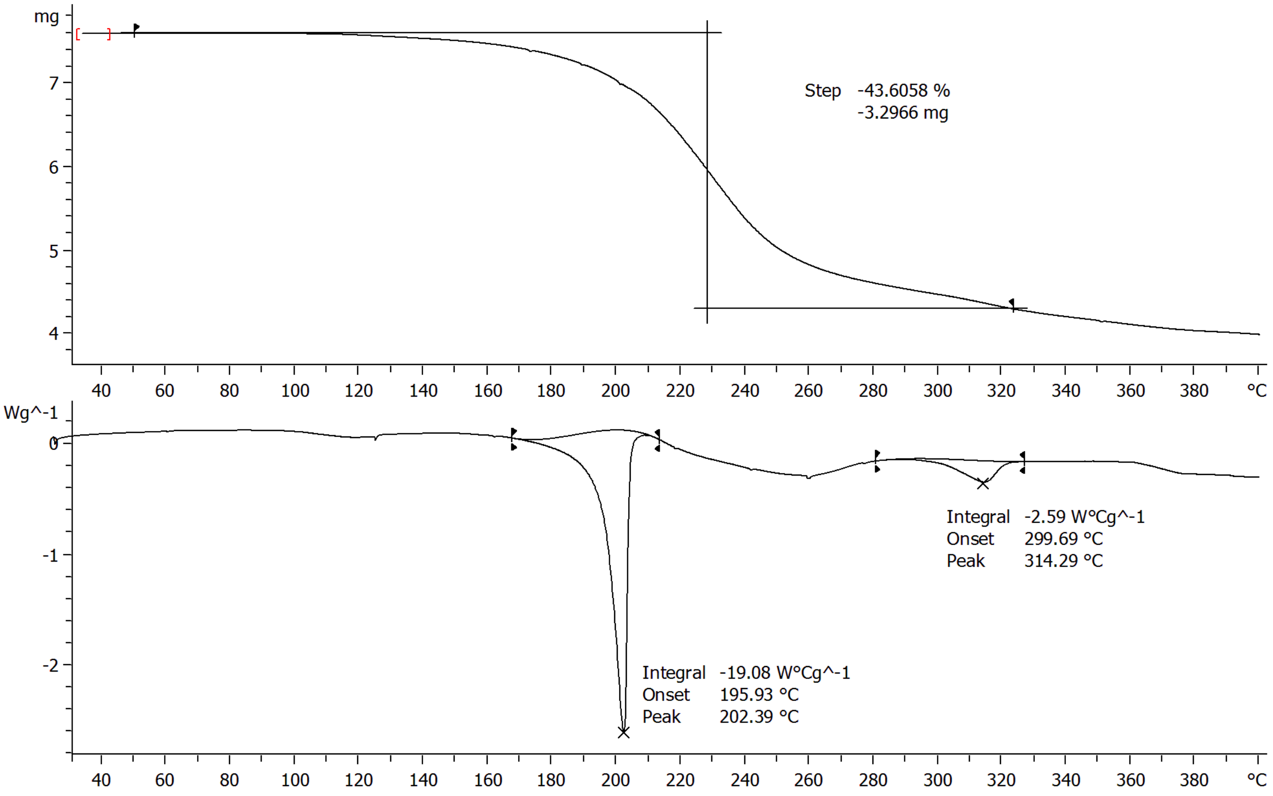

any transient formation of flsL. The flsQ form melts and decomposes above 327.4 °C (onset temperature).

Figure S27. TGA and DSC corves of flsL:acridine$_2$ cocrystal. Dissociation of the cocrystal occurs at 202 °C (weight loss 43.6%, theoretical – 51.9%). The weight loss step in the TGA curve occurs over a wide temperature range – possible sign of chemical degradation occurring simultaneously with cocrystal dissociation.

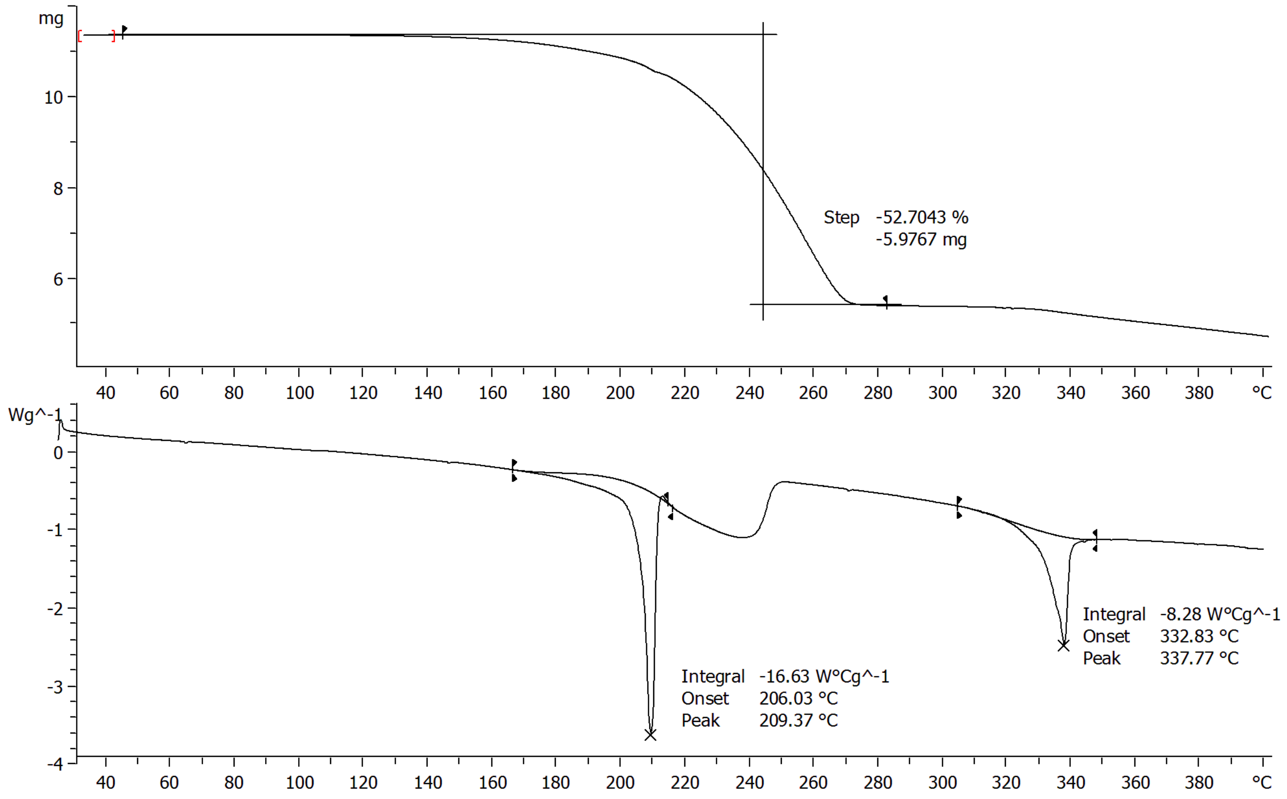

Figure S28. TGA and DSC curves of flsL:phenanthridine$_2$ cocrystal. Dissociation occurs at 206 °C (onset temperature, weight loss 52.7%, theoretical – 51.9%) followed by melting and decomposition of flsQ at 332.8 °C (onset temperature).

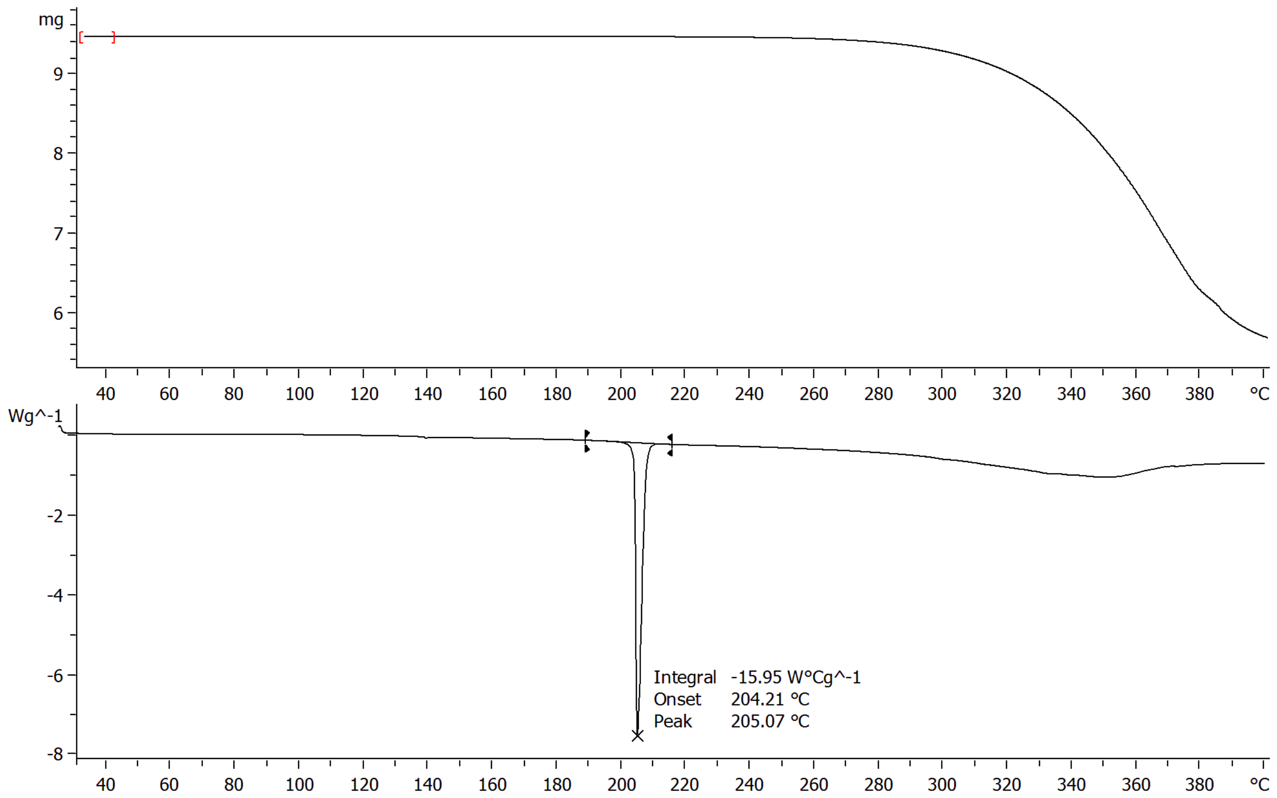

Figure S29. DSC and TGA curves of diacetylfluorescein. The onset of melting is at 204.2 °C.

6. Crystal Structure Prediction (CSP)

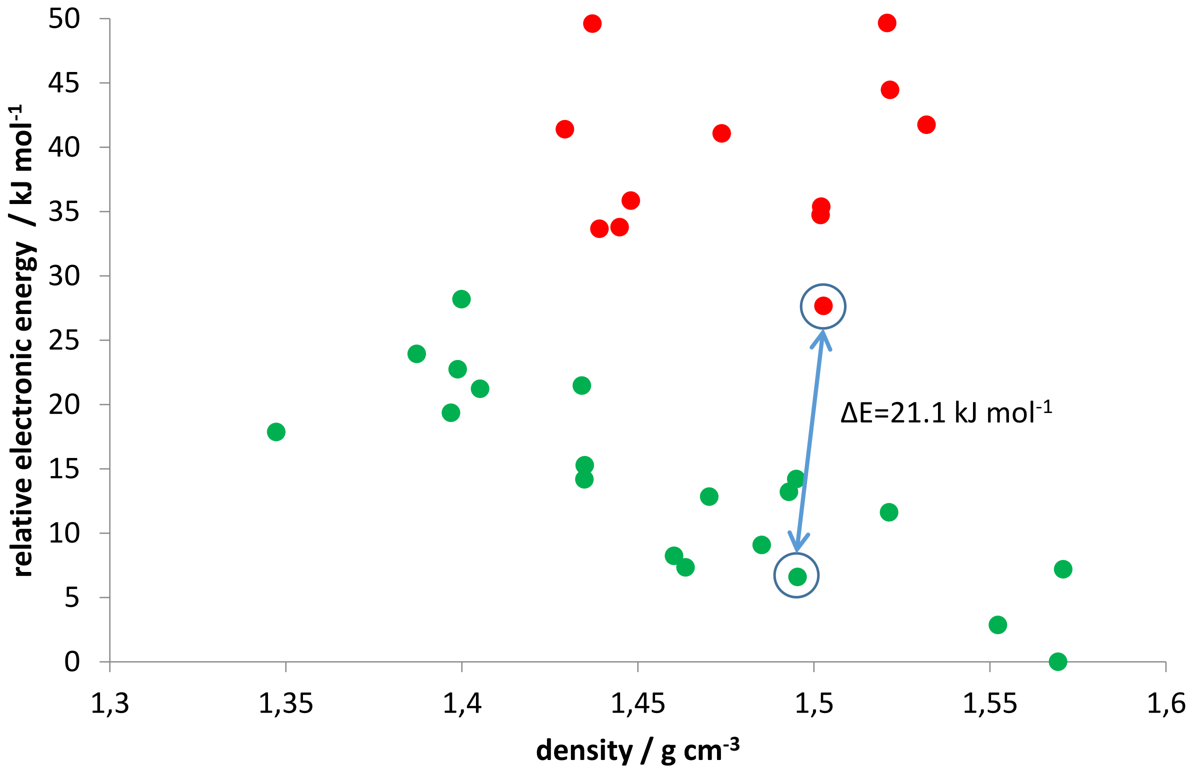

Figure S30. Combined crystal energy landscape of flsZ (green) and flsL (red). The lowest energy predicted structure of flsL is 21.1 kJ mol^{-1} higher than the experimental structure of flsZ.

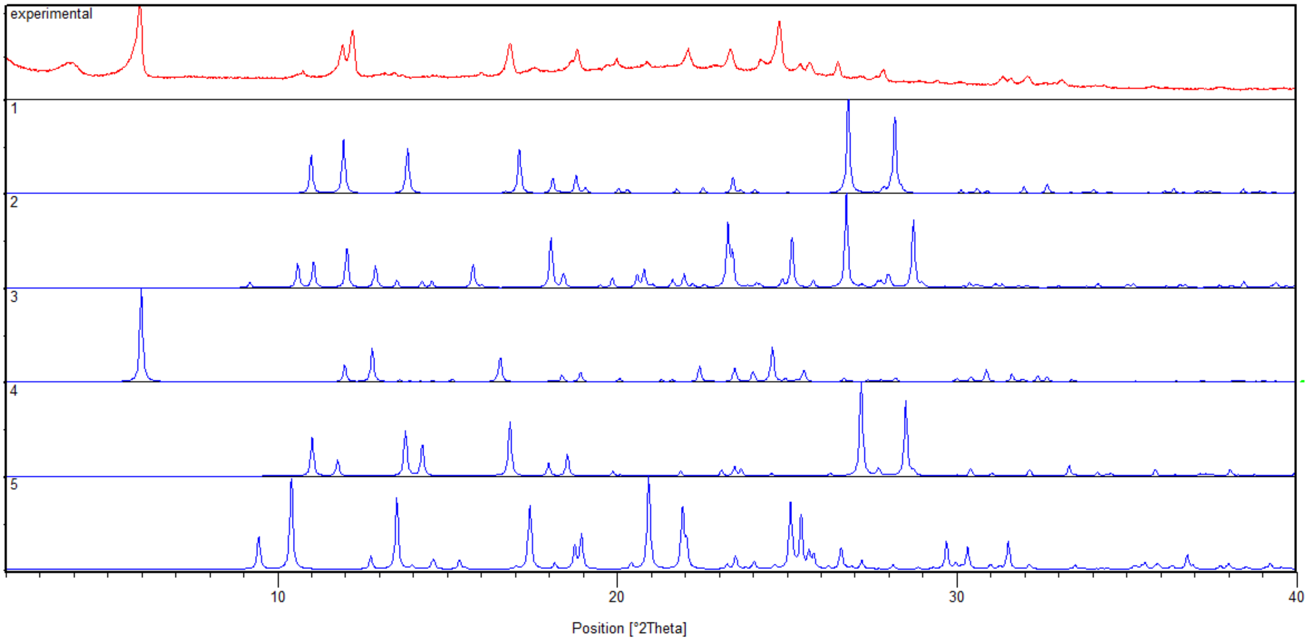

Figure S31. Comparison of the experimental flsZ PXRD pattern (top, red) with the calculated patterns of predicted structures #1-5. It is evident that structure #3 provides the best match with experiment. The correctness of this structural assignment is validated by the successful Rietveld refinement (Figure S4) and by the fit to the electron diffraction data.

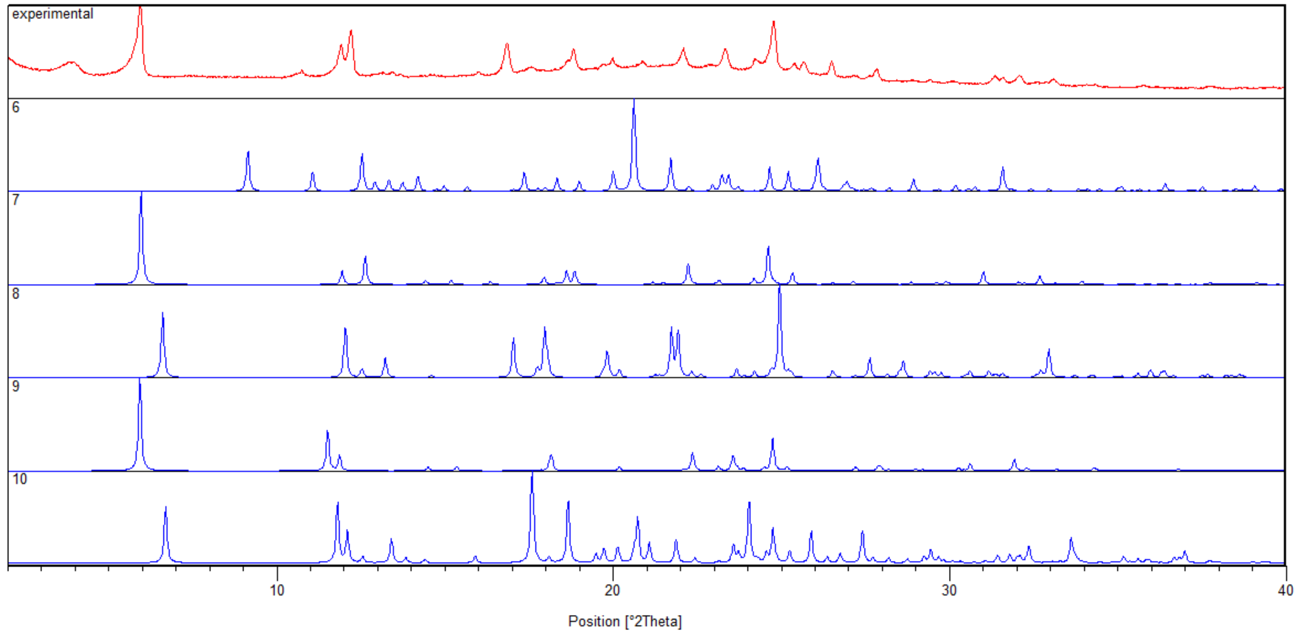

Figure S32. Comparison of the experimental flsZ PXRD pattern (top, red) with the calculated patterns of predicted structures #6-10. Structures #7 and #9 display certain degree of similarity with the experimental pattern, however Rietveld refinement of these structures did not produce a satisfactory fit. The structures #7 and #9 were also found to be inconsistent with the electron diffraction data.

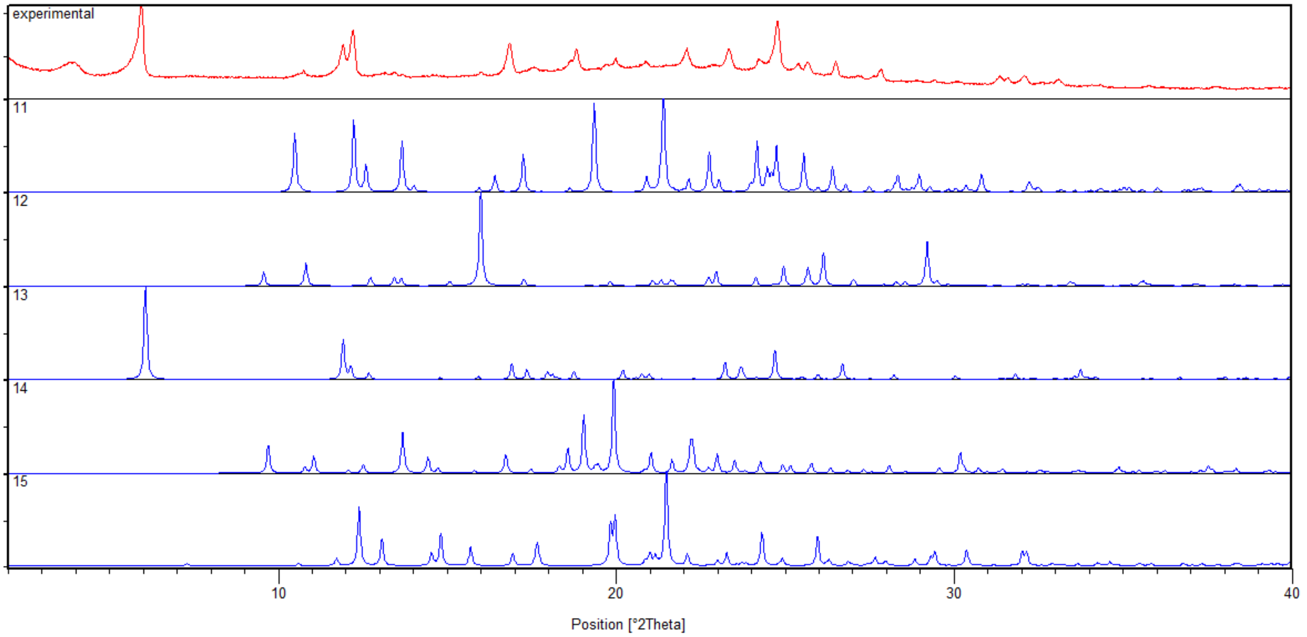

Figure S33. Comparison of the experimental flsZ PXRD pattern (top, red) with the calculated patterns of predicted structures #11-15. Structure #13 shows certain degree of similarity with the experimental pattern, however Rietveld refinement and comparison with the experimental TEM data allow to discard the structure as a match.

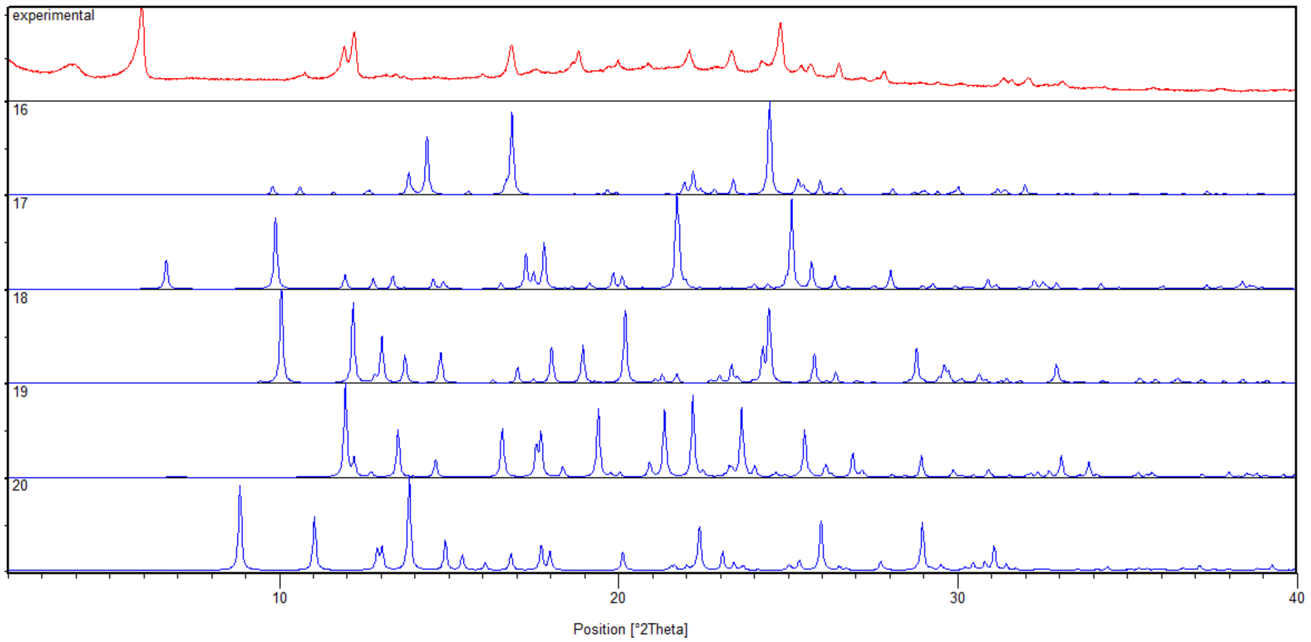

Figure S34. Comparison of the experimental flsZ PXRD pattern (top, red) with the calculated patterns of predicted structures #16-20. None of the structures show similarity with the experimental powder pattern.

7. Solid-state ^{13}C NMR spectroscopy

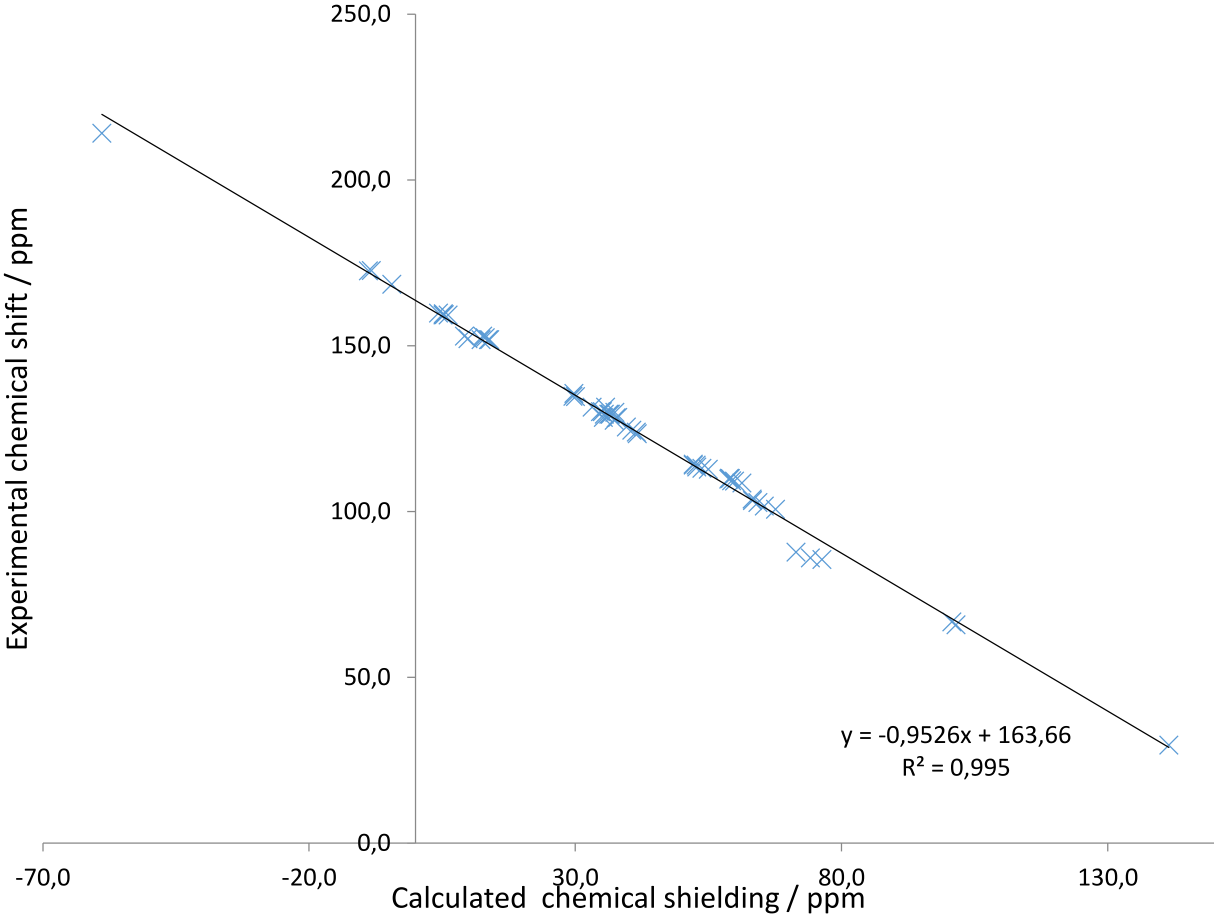

Figure S35. Derivation of the regression equation for converting the calculated chemical shieldings to the chemical shifts. The structures of flsL:acetone monosolvate form I, flsL:dioxane hemisolvate and flsL:dioxane hemipentasolvates were used in the analysis. The derived equation was used to predict the NMR spectra of flsZ and flsQ.

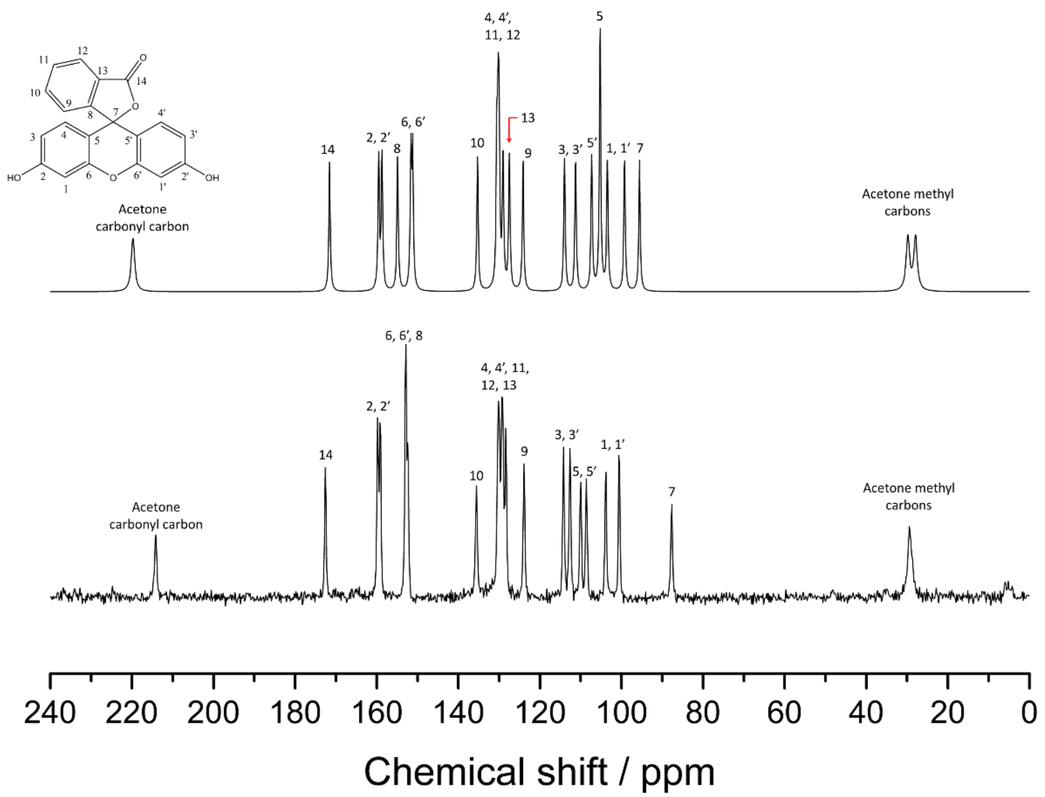

Figure S36. Comparison of the calculated (top) and experimental (bottom) ^{13}C solid-state NMR spectra of flsL:acetone monosolvate form I. The peaks in the calculated spectrum were constructed using Lorentzian curves positioned at the computed chemical shifts. Peak intensities were assumed to be proportional to the number of contributing atoms.

Table S2. Calculated and experimentally measured chemical shifts of flsL:acetone monosolvate form I.

atom number	calculated shielding / ppm	experimental shift / ppm	calculated shift / ppm
Acetone methyl carbons	141.53	29.50	28.84
7	71.48	87.70	95.57
1, 1'	67.63	100.65	99.24
	63.21	103.75	103.45
5, 5'	61.33	108.60	105.24
	59.17	109.96	107.29
3, 3'	55.02	112.68	111.25
	52.15	114.23	113.98
9	41.48	123.93	124.15
12	35.30	128.39	130.03
13	37.96	128.39	127.50
4' 4'	36.33	129.36	129.05
	35.07	130.13	130.25
11	34.73	130.13	130.58
10	29.76	135.57	135.31
8	9.19	152.83	154.91
6, 6'	13.08	152.44	151.20
	12.62	152.83	151.64
2, 2'	5.18	159.23	158.73
	4.32	159.81	159.54
14	-8.32	172.62	171.59
Acetone carbonyl carbon	-58.91	214.10	219.78

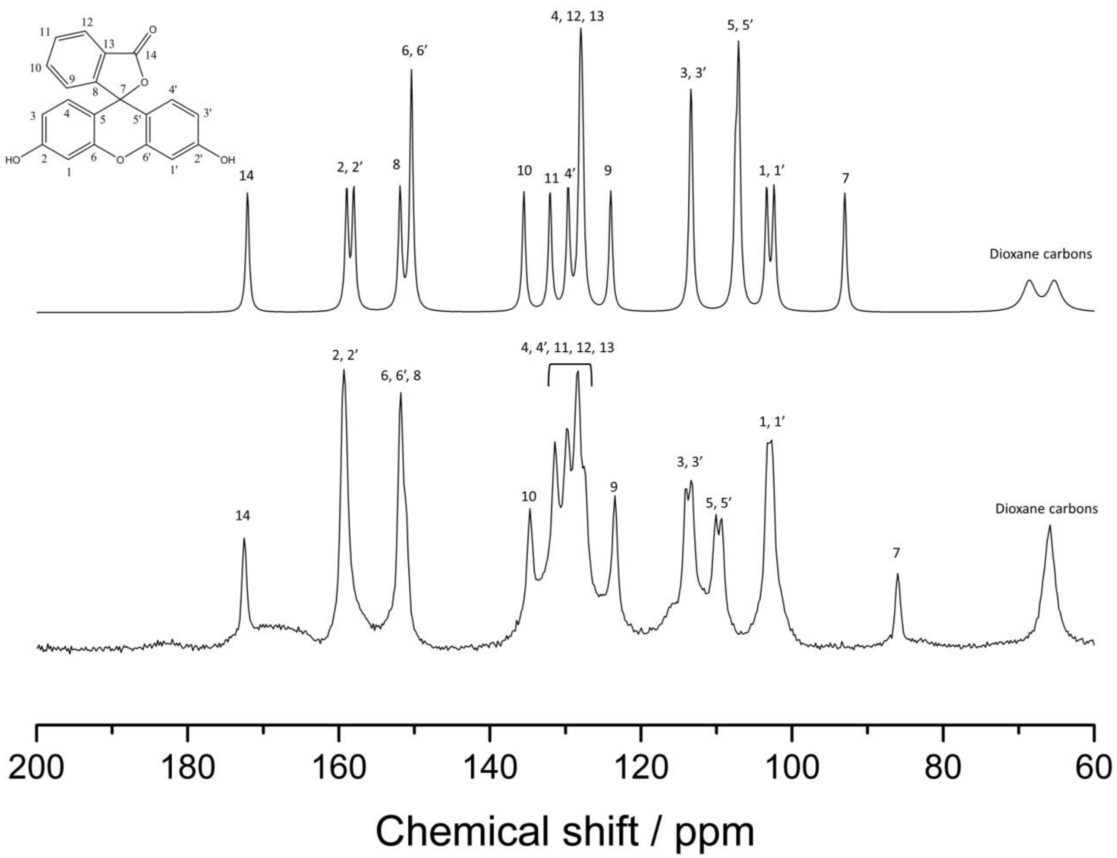

Figure S37. Comparison of the calculated (top) and experimental (bottom) ^{13}C solid-state NMR spectra of flsL:dioxane hemisolvate. The peaks in the calculated spectrum were constructed using Lorentzian curves positioned at the computed chemical shifts. Peak intensities were assumed to be proportional to the number of contributing atoms.

Table S3. Calculated and experimentally measured chemical shifts of flsL:dioxane hemisolvate.

atom number	calculated shielding / ppm	experimental shift / ppm	calculated shift / ppm
Dioxane carbons	101.54	65.84	66.93
7	74.16	86.02	93.02
1, 1'	64.33	102.70	102.38
	63.30	103.28	103.36
5, 5'	59.41	109.30	107.07
	58.97	110.07	107.49
3, 3'	52.85	113.37	113.32
	52.71	113.95	113.45
9	41.63	123.46	124.00
12	37.33	127.53	128.10
13	37.70	128.31	127.75
4, 4'	37.46	129.86	127.98
	35.69	131.41	129.66
11	33.18	131.41	132.05
10	29.54	134.71	135.52
8	12.33	151.78	151.91
6, 6'	13.91	151.78	150.41
2, 2'	5.41	159.34	158.51
14	-8.87	172.54	172.11

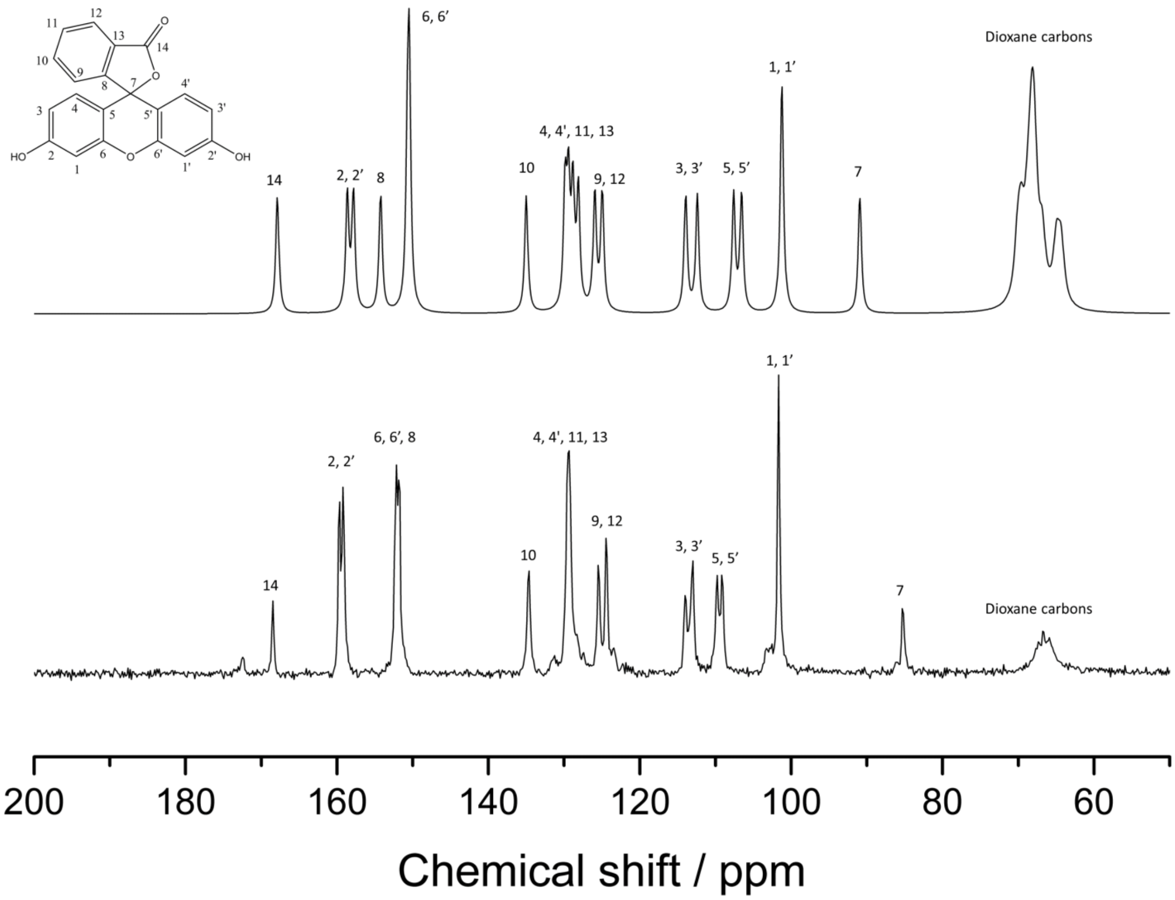

Figure S38. Comparison of the calculated (top) and experimental (bottom) ^{13}C solid-state NMR spectra of flsL:dioxane hemipentasolvate. The peaks in the calculated spectrum were constructed using Lorentzian curves positioned at the computed chemical shifts. Peak intensities were assumed to be proportional to the number of contributing atoms.

Table S4. Calculated and experimentally measured chemical shifts of flsL:dioxane hemipentasolvate.

atom number	calculated shielding / ppm	experimental shift / ppm	calculated shift / ppm
Dioxane carbons	100.77	66.69	67.67
7	76.35	85.53	90.93
1, 1'	65.54	101.66	101.23
5, 5'	59.94	109.17	106.56
	58.84	109.76	107.61
3, 3'	53.80	112.99	112.41
	52.19	114.02	113.94
9	40.62	124.47	124.97
12	39.58	125.50	125.96
4, 4'	36.62	129.33	128.78
11	35.47	129.33	129.87
13	36.55	129.33	128.84
10	30.08	134.63	135.01
6, 6'	13.72	151.84	150.60
8	9.88	152.14	154.25
2, 2'	6.10	159.20	157.85
	5.25	159.64	158.66
14	-4.45	168.47	167.90

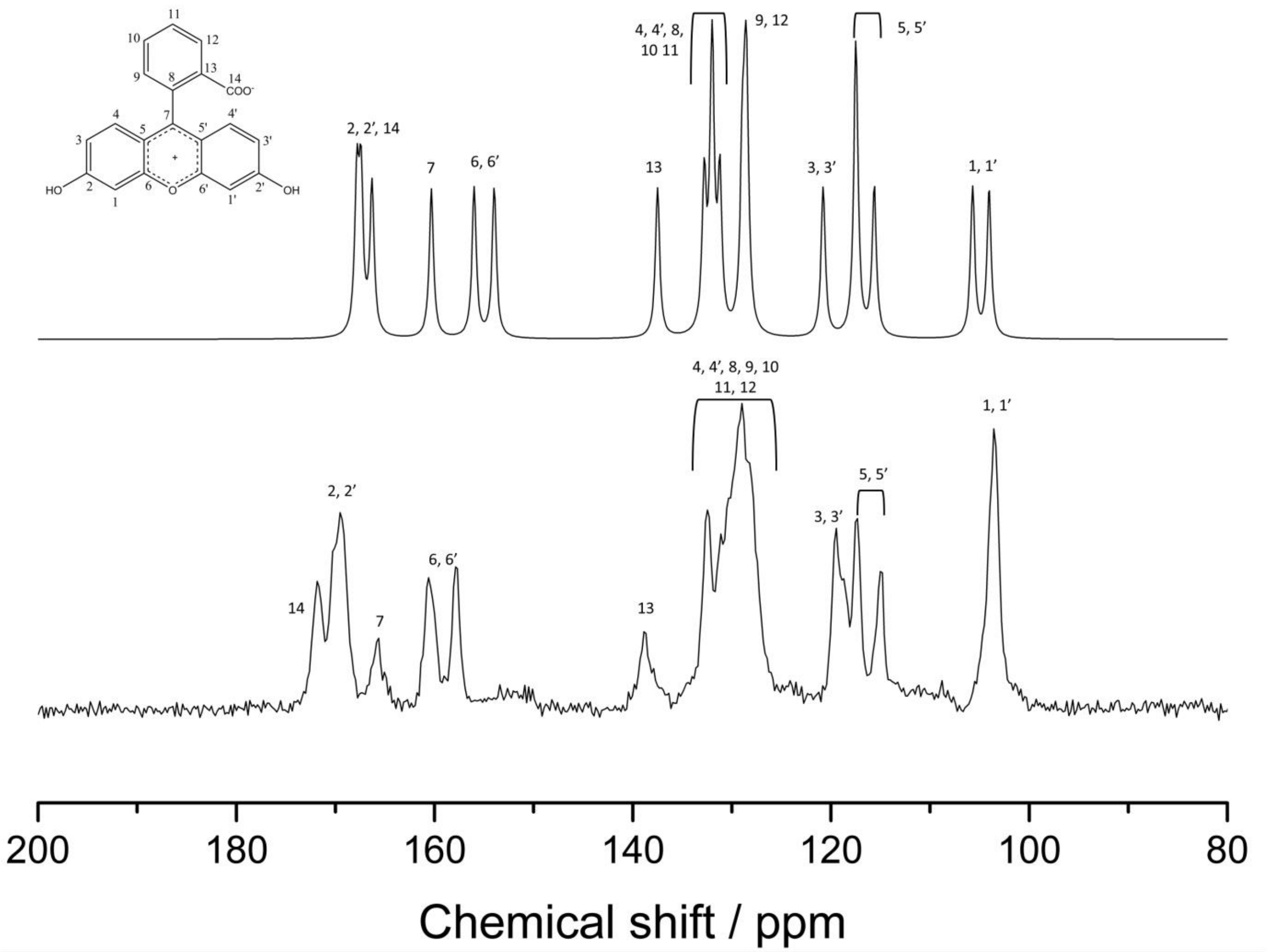

Figure S39. Comparison of the calculated (top) and experimental (bottom) 13C solid-state NMR spectra of flsZ. The peaks in the calculated spectrum were constructed using Lorentzian curves positioned at the computed chemical shifts. Peak intensities were assumed to be proportional to the number of contributing atoms.

Table S5. Calculated and experimentally measured chemical shifts of flsZ.

atom number	calculated shielding / ppm	experimental shift / ppm	calculated shift / ppm
1, 1'	61.71	103.56	104.88
5, 5'	49.46	115.00	116.55
3, 3'	46.72	119.47	119.15
4, 4'	34.86	128.97	130.46
12	33.18	128.97	132.05
11	34.05	128.97	131.22
10	36.75	128.97	128.65
9	36.93	128.97	128.48
8	32.40	132.46	132.80
13	27.45	138.86	137.51
6, 6'	9.09	157.87	155.01
7	3.49	160.59	160.34
2, 2'	-3.39	165.30	166.88
14	-4.41	169.51	167.86

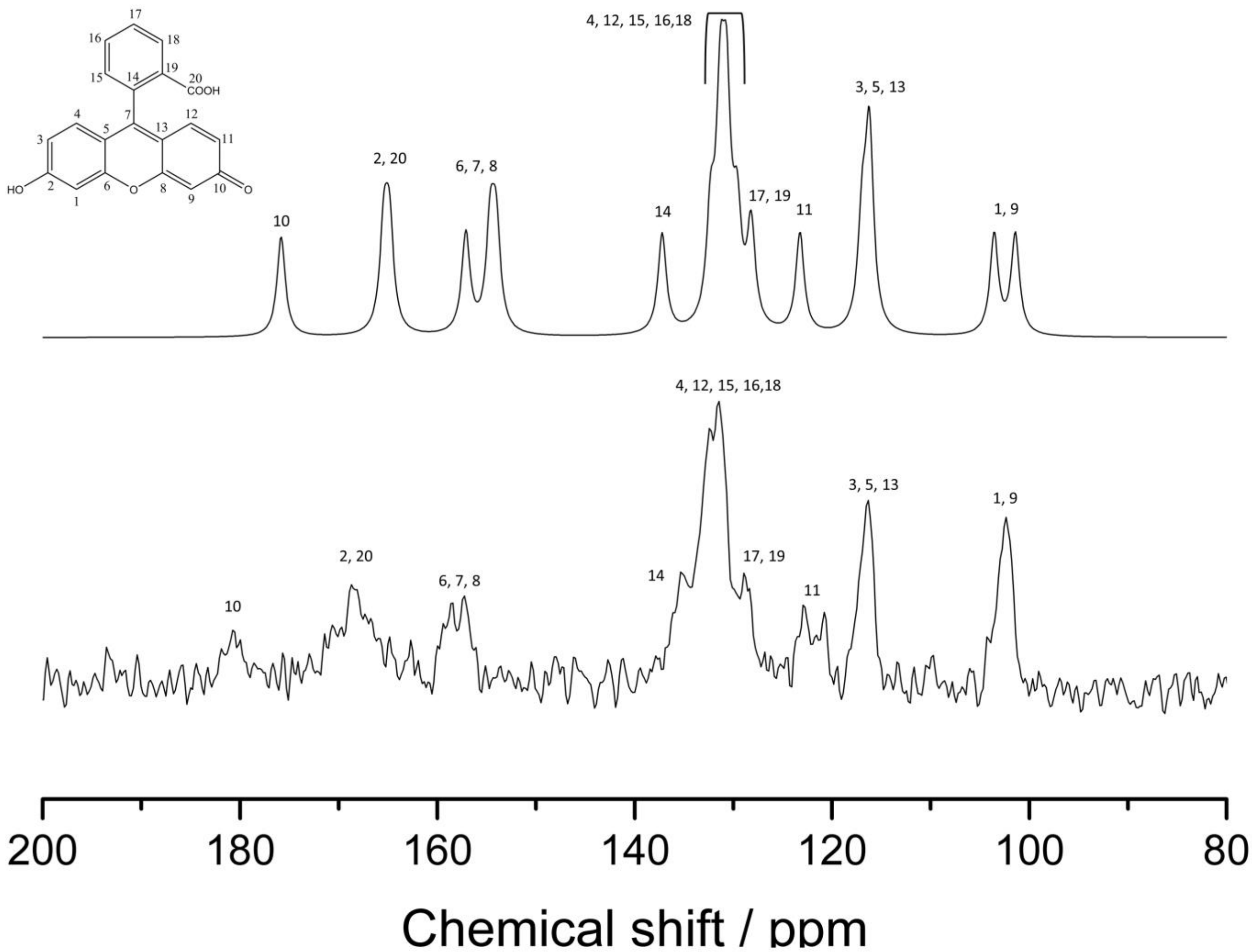

Figure S40. Comparison of the calculated (top) and experimental (bottom) 13C solid-state NMR spectra of flsQ. The peaks in the calculated spectrum were constructed using Lorentzian curves positioned at the computed chemical shifts. Peak intensities were assumed to be proportional to the number of contributing atoms. We attribute the poorer signal-to-noise ratio in the spectrum of flsQ to the fact that cross polarization (CP) dynamics differ from sample to sample and time constraints preclude parameter optimization for each one; the CP parameters may not have been optimal for this sample but this does not affect the overall conclusions.

Table S6. Calculated and experimentally measured chemical shifts of flsZ.

atom number	calculated shielding / ppm	experimental shift / ppm	calculated shift / ppm
1, 9	64.22	102.36	102.49
3, 5, 13	49.55	116.33	116.46
11	42.42	122.92	123.25
17, 19	36.48	128.93	128.91
12, 18	34.61	131.46	130.69
4, 15, 16	33.61	132.43	131.65
14	27.75	135.34	137.23
6, 8	9.775	157.26	154.35
7	6.86	158.42	157.13
2, 20	-1.56	168.7	165.15
10	-12.8	180.73	175.85

Table S7. Comparison of the calculated and experimental ^{13}C chemical shifts for the red form of fluorescein. The shifts were calculated for the proton position corresponding to both flsQ and flsZ. It is evident that flsQ shifts are in better overall agreement with the experimental data.

Atom	Chemical shift / ppm			Chemical shift difference / ppm	
	Calculated as quinoid	Calculated as zwitterionic	Experimentally measured	Calculated as quinoid	Calculated as zwitterionic
1	101.42	101.95	102.36	-0.94	-0.41
9	103.56	102.25	102.36	1.20	-0.11
3	116.15	119.06	116.33	-0.18	2.73
13	116.27	116.63	116.33	-0.06	0.30
5	116.94	117.33	116.33	0.61	1.00
11	123.24	117.66	122.92	0.32	-5.26
17	128.20	128.43	128.93	-0.73	-0.50
19	129.61	135.15	128.93	0.68	6.22
12	130.64	132.41	131.46	-0.82	0.95
18	130.72	130.24	131.46	-0.74	-1.22
4	131.28	133.57	132.43	-1.15	1.14
15	131.34	130.32	132.43	-1.09	-2.11
16	132.29	130.30	132.43	-0.14	-2.13
14	137.21	135.44	135.34	1.87	0.10
6	154.04	154.25	157.26	-3.22	-3.01
8	154.62	153.02	157.26	-2.64	-4.24
7	157.11	165.18	158.42	-1.31	6.76
2	164.85	165.03	168.70	-3.85	-3.67
20	165.40	167.63	168.70	-3.30	-1.07
10	175.83	167.70	180.73	-4.90	-13.03
			RMSD	2.00	4.15

Table S8. Comparison of the calculated and experimental ^{13}C chemical shifts for the yellow form of fluorescein. The shifts were calculated for the proton position corresponding to both flsQ and flsZ. It is evident that flsZ shifts are in better overall agreement with the experimental data.

Atom	Chemical shift / ppm			Chemical shift difference / ppm	
	Calculated as quinoid	Calculated as zwitterionic	Experimentally measured	Calculated as quinoid	Calculated as zwitterionic
1	105.34	105.70	103.56	1.78	2.14
9	106.39	104.05	103.56	2.83	0.49
3	119.66	120.79	119.47	0.19	1.32
13	113.87	115.63	115.00	-1.13	0.63
5	115.89	117.45	115.00	0.89	2.45
11	122.90	117.51	117.33	5.57	0.18
17	130.02	131.21	128.97	1.05	2.24
19	132.81	137.50	138.86	-6.05	-1.36
12	126.53	128.95	128.97	-2.44	-0.02
18	131.38	132.04	128.97	2.41	3.07
4	131.24	131.95	132.46	-1.22	-0.51
15	128.74	128.47	128.97	-0.23	-0.50
16	130.25	128.64	128.97	1.28	-0.33
14	135.86	132.79	132.46	3.40	0.33
6	155.56	156.00	157.87	-2.31	-1.87
8	154.86	153.97	157.87	-3.01	-3.90
7	151.46	160.32	160.59	-9.13	-0.27
2	164.75	166.31	169.51	-4.76	-3.20
20	167.40	167.84	171.84	-4.44	-4.00
10	174.50	167.42	165.63	8.87	1.79
			RMSD	4.05	1.98

8. Optical spectroscopy and calculations.

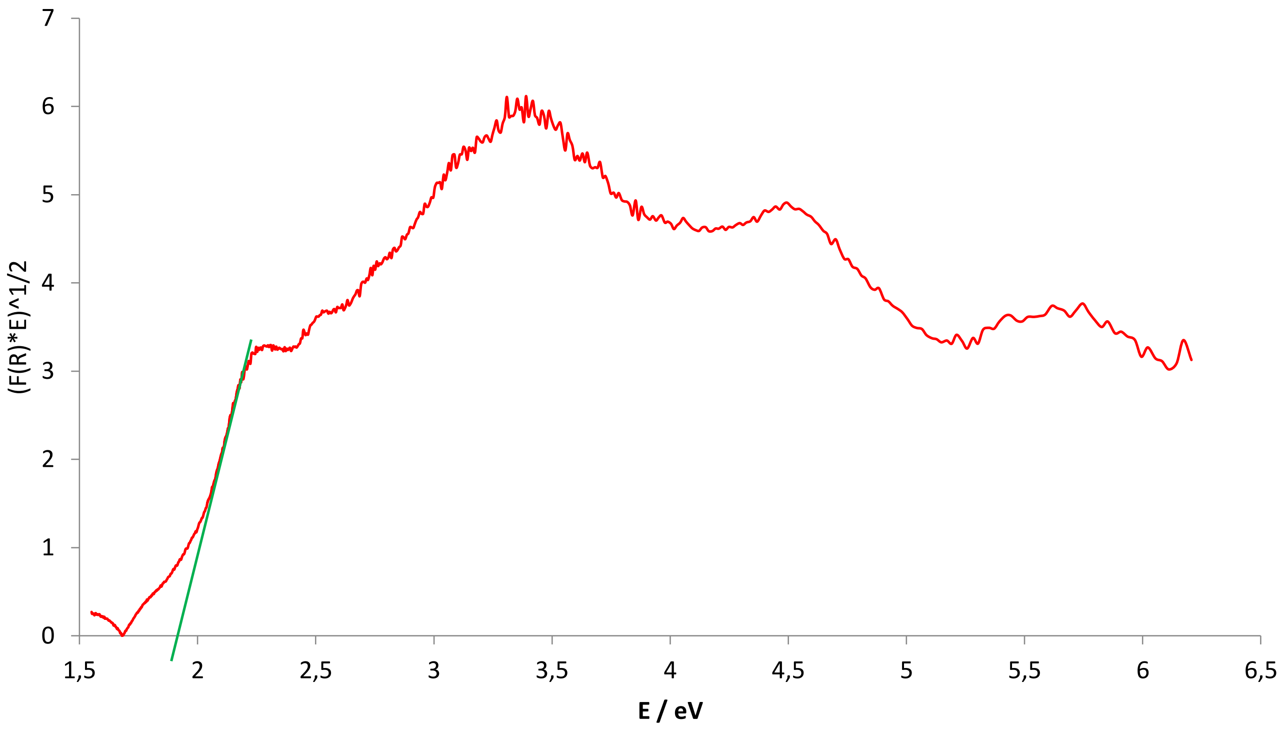

Figure S41. Tauc plot of fluorescein red form (flsQ). The band gap is calculated by extrapolating the linear region of the plot to the *x*-axis (shown in green).

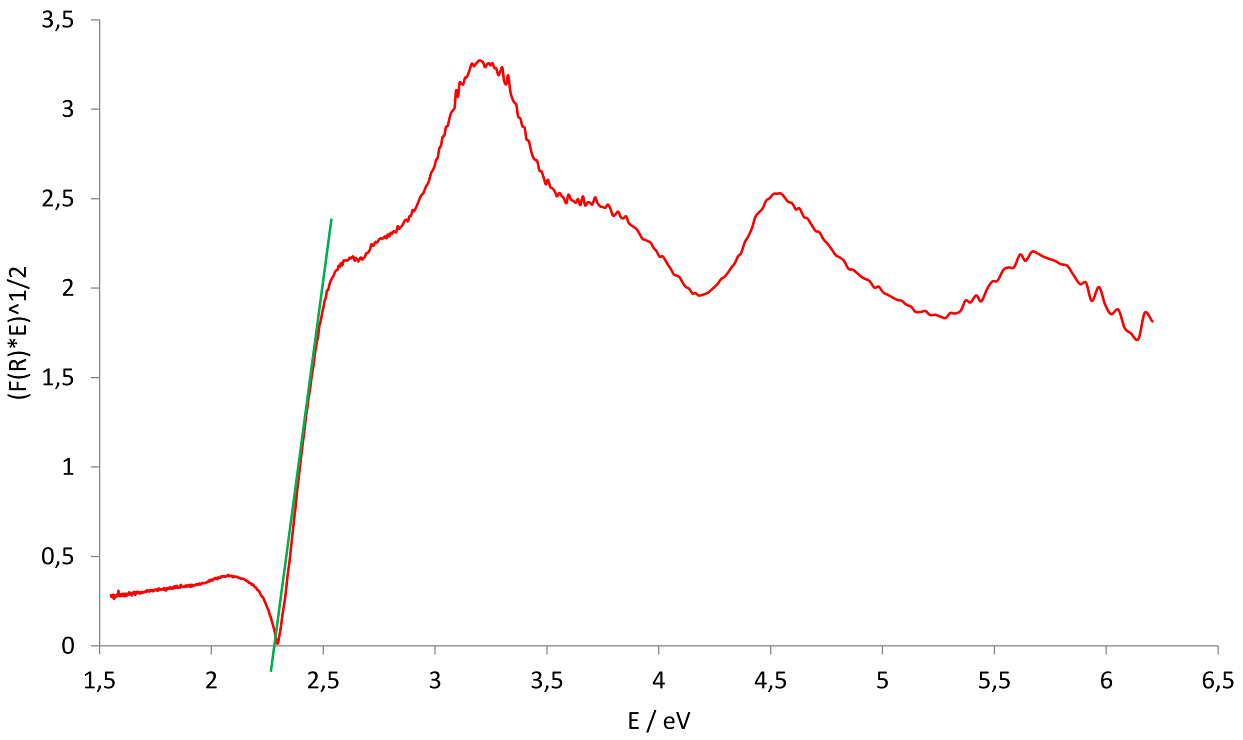

Figure S42. Tauc plot of fluorescein yellow form (flsZ). The band gap is calculated by extrapolating the linear region of the plot to the x-axis (shown in green).

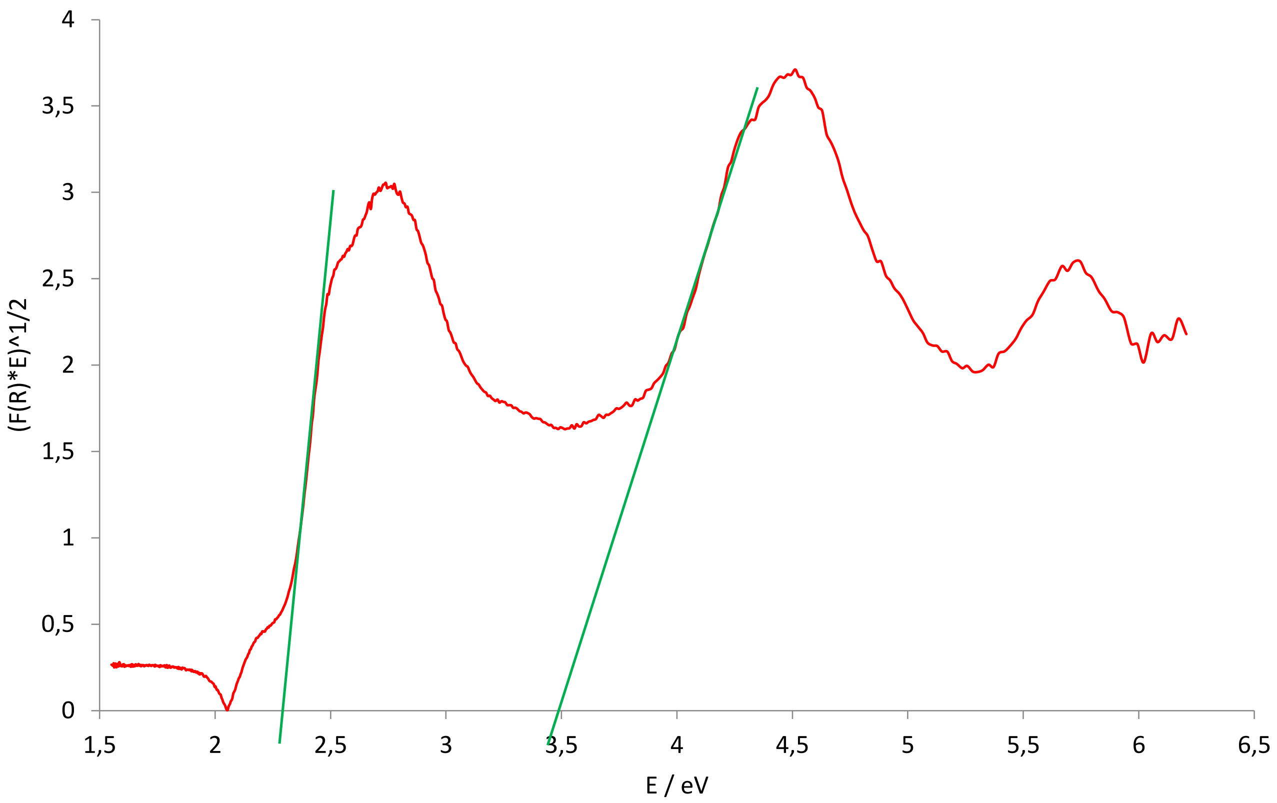

Figure S43. Tauc plot of flsL:acetone monosolvate. The line crossing the x-axis at ~2.3 eV corresponds to the bad gap of the flsZ surface layer. The band gap of the bulk acetone solvate is ~3.5 eV.

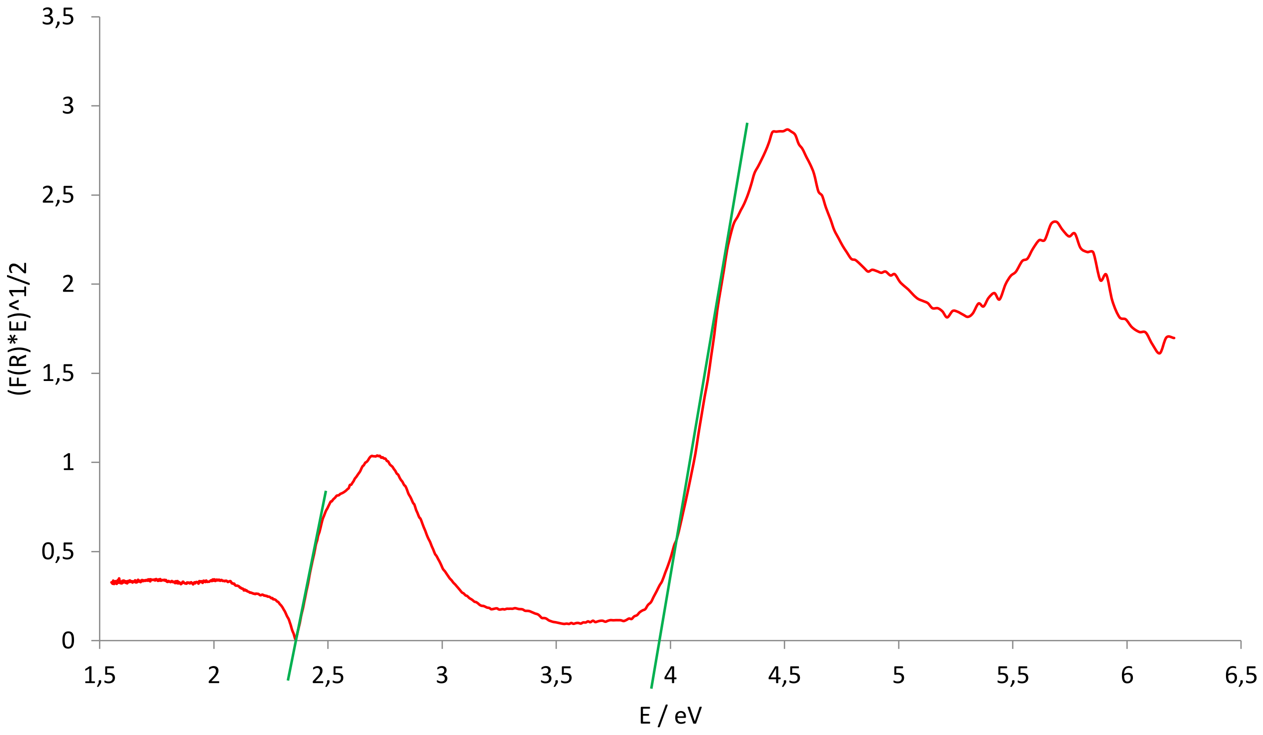

Figure S44. Tauc plot of flsL:dioxane hemisolvate. The line crossing the x-axis at ~2.3 eV corresponds to the bad gap of the flsZ surface layer. The band gap of the bulk dioxane solvate is ~3.9 eV.

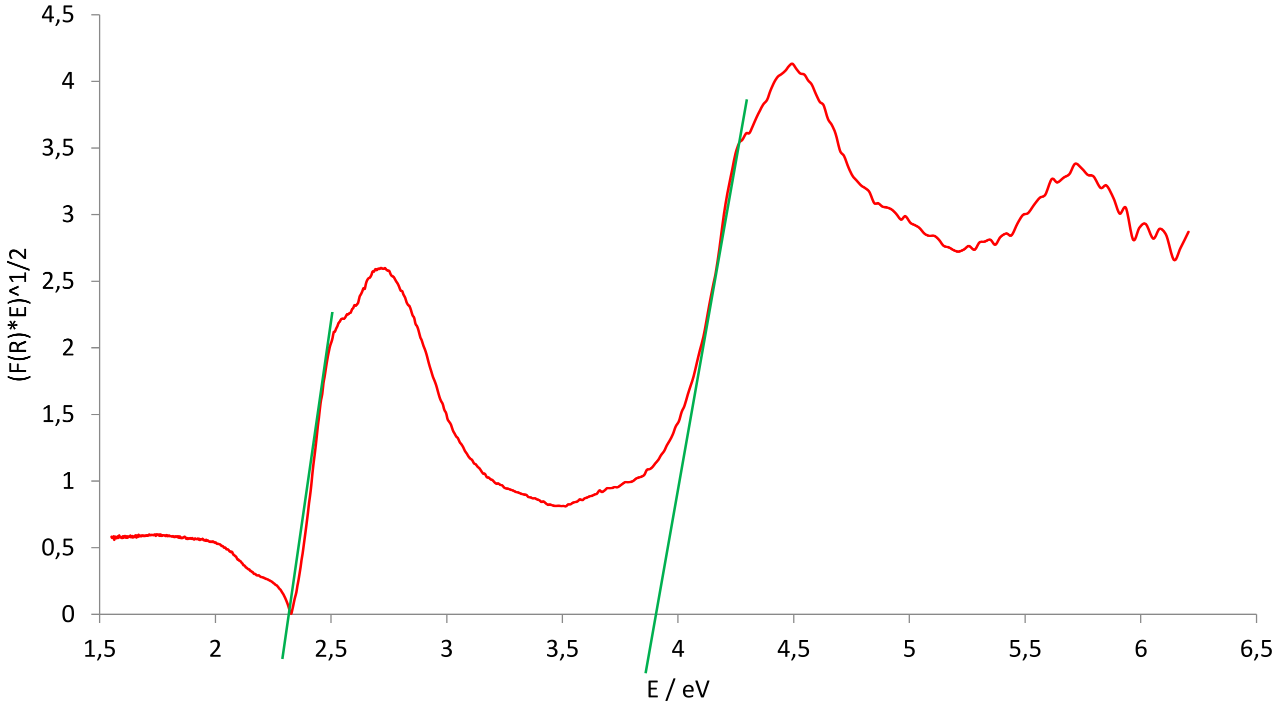

Figure S45. Tauc plot of flsL:dioxane hemipentasolvate. The line crossing the x-axis at ~2.3 eV corresponds to the bad gap of the flsZ surface layer. The band gap of the bulk dioxane solvate is ~3.9 eV.

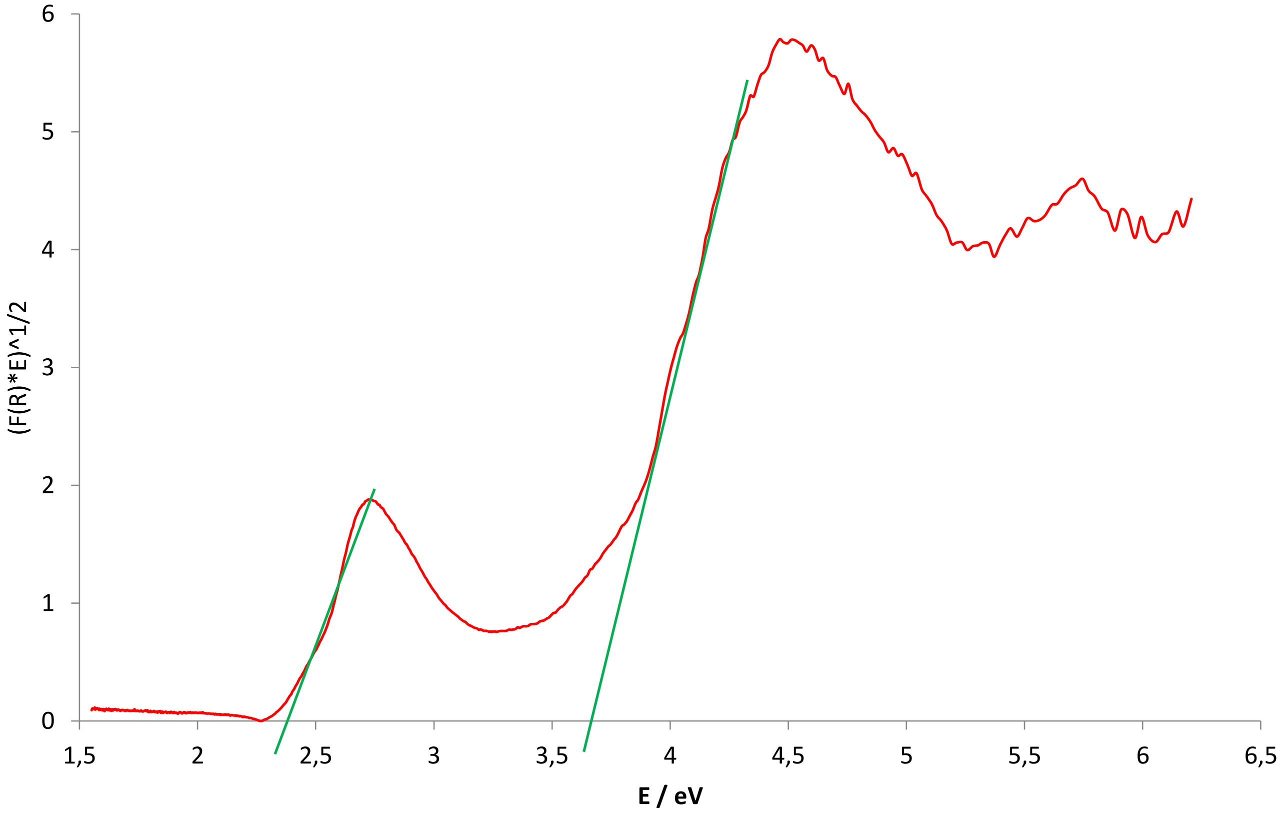

Figure S46. Tauc plot of the flsL:pyrazine cocrystal in the colored (yellow-green) form. The line crossing the x-axis at ~2.3 eV corresponds to the bad gap of the flsZ surface layer. The band gap of the bulk pyrazine cocrystal is ~3.6 eV.

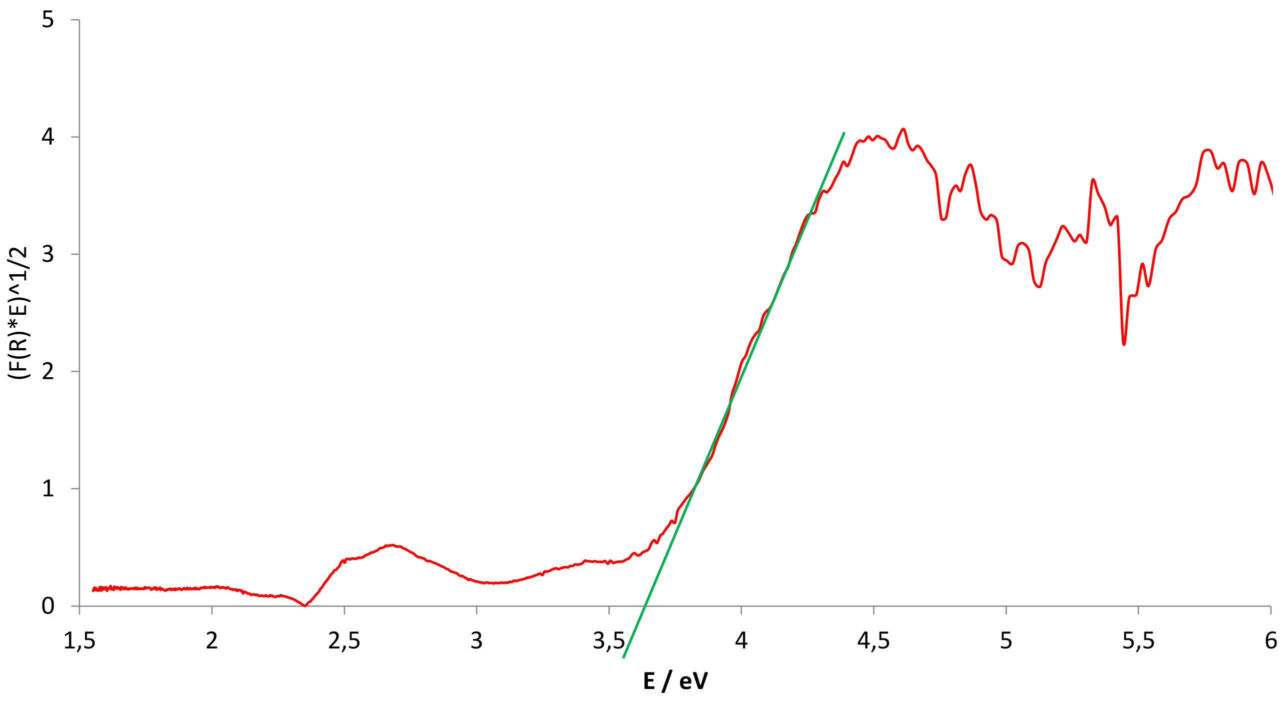

Figure S47. Tauc plot of the flsL:pyrazine cocrystal in the grey form. The feature corresponding to the flsZ surface layer has greatly diminished compared to the plot on the Figure S35. The band gap of the bulk pyrazine cocrystal is ~3.6 eV.

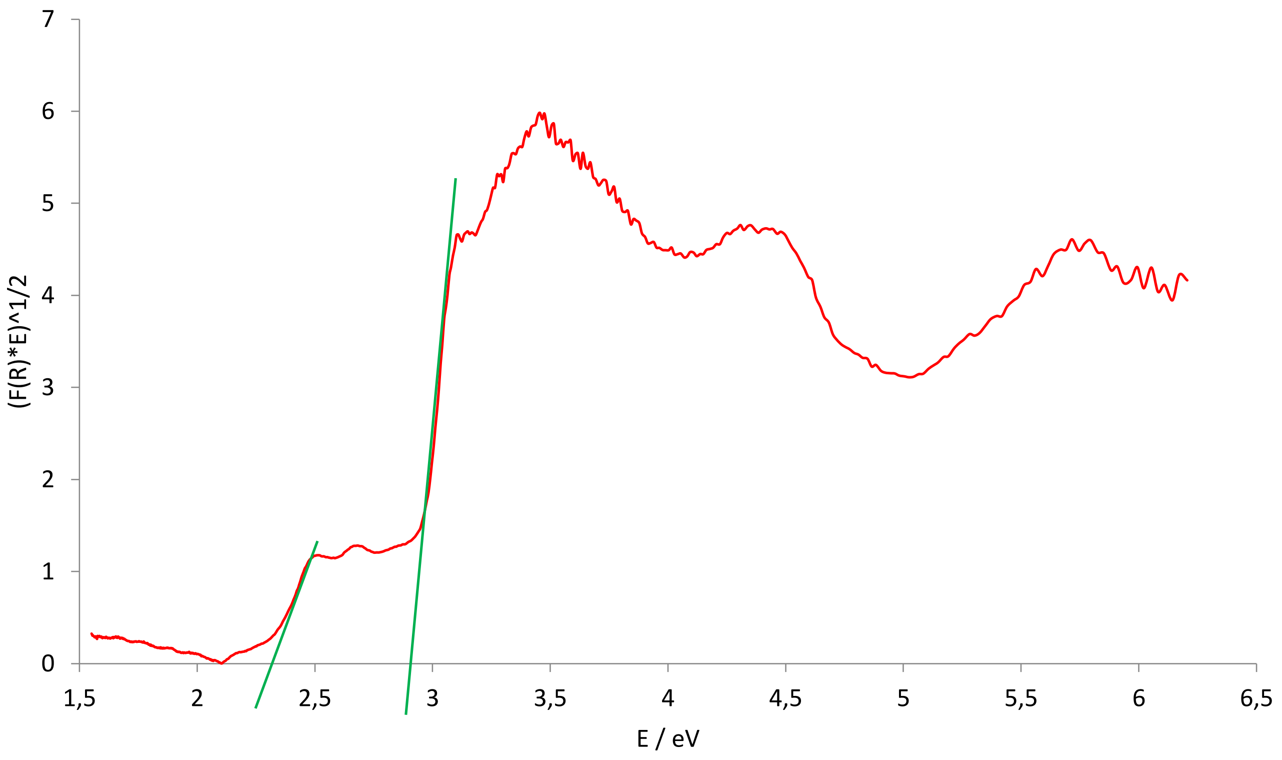

Figure S48. Tauc plot of the flsL:acridine cocrystal. The line crossing the x-axis at ~2.3 eV corresponds to the bad gap of the flsZ surface layer. The band gap of the bulk cocrystal is ~2.9 eV.

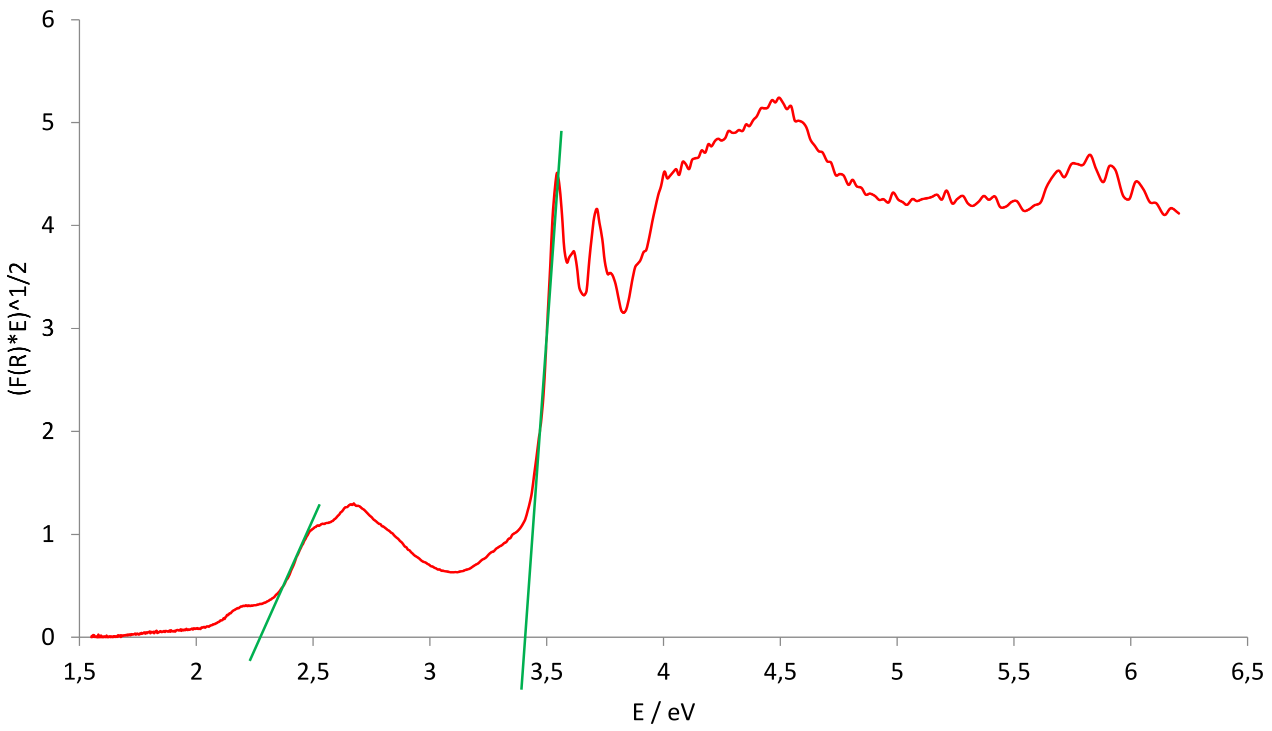

Figure S49. Tauc plot of the flsL:phenanthridine cocrystal. The line crossing the x-axis at ~2.3 eV corresponds to the bad gap of the flsZ surface layer. The band gap of the bulk cocrystal is ~3.4 eV.

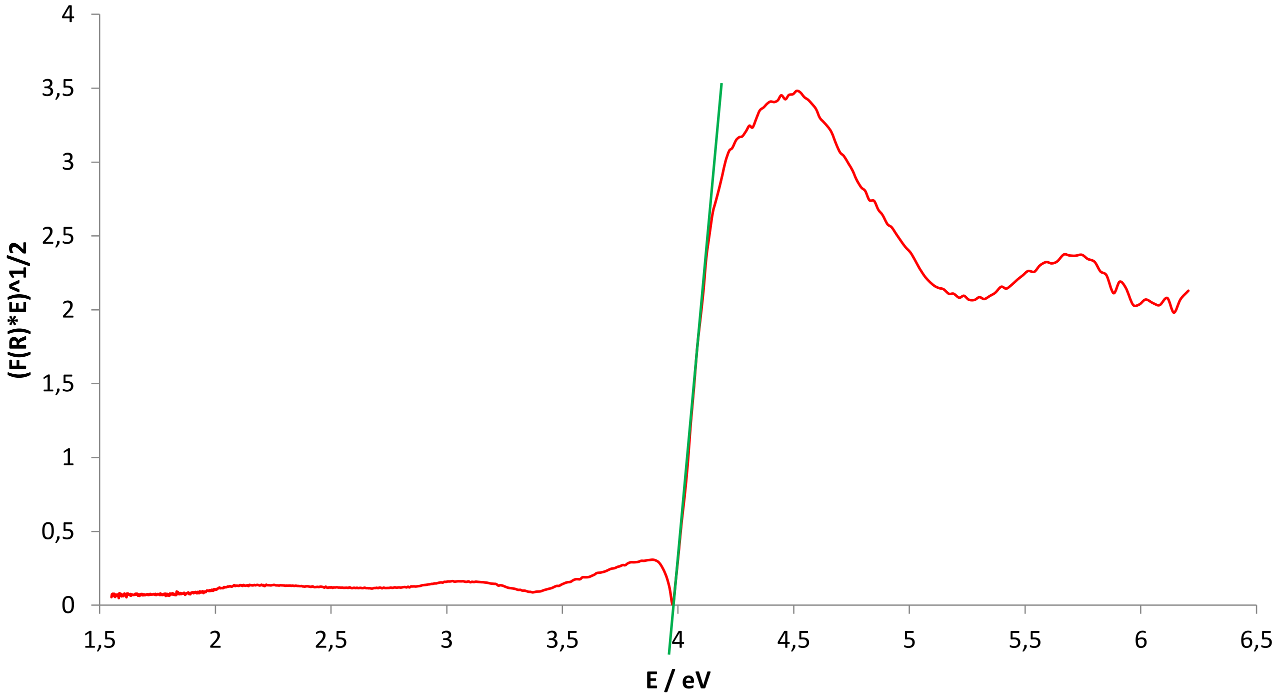

Figure S50. Tauc plot of diacetylfluorescein lactoid form. The band gap of the bulk material is 4.0 eV. There is no evidence for the presence of zwitterionic impurity, which is consistent with the essentially colorless (grey) appearance of the material.

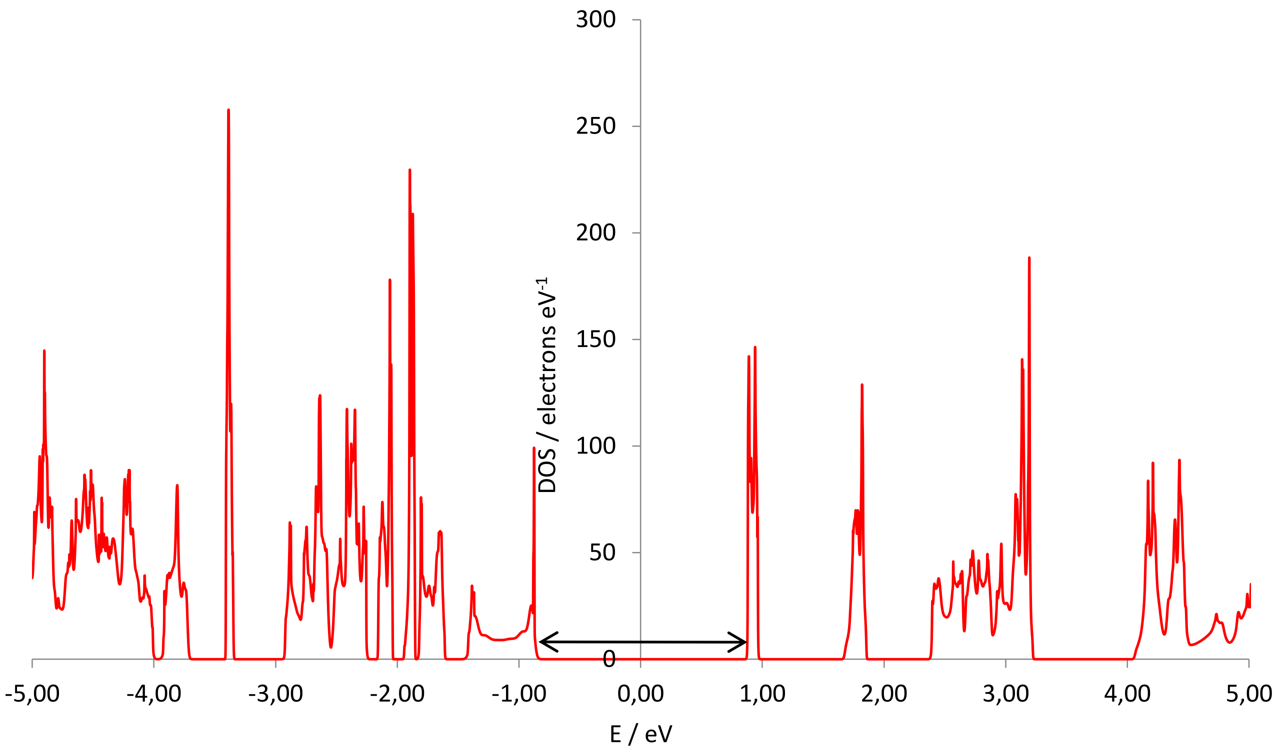

Figure S51. The DOS plot for flsQ (red form). The arrow shows the position of the band gap.

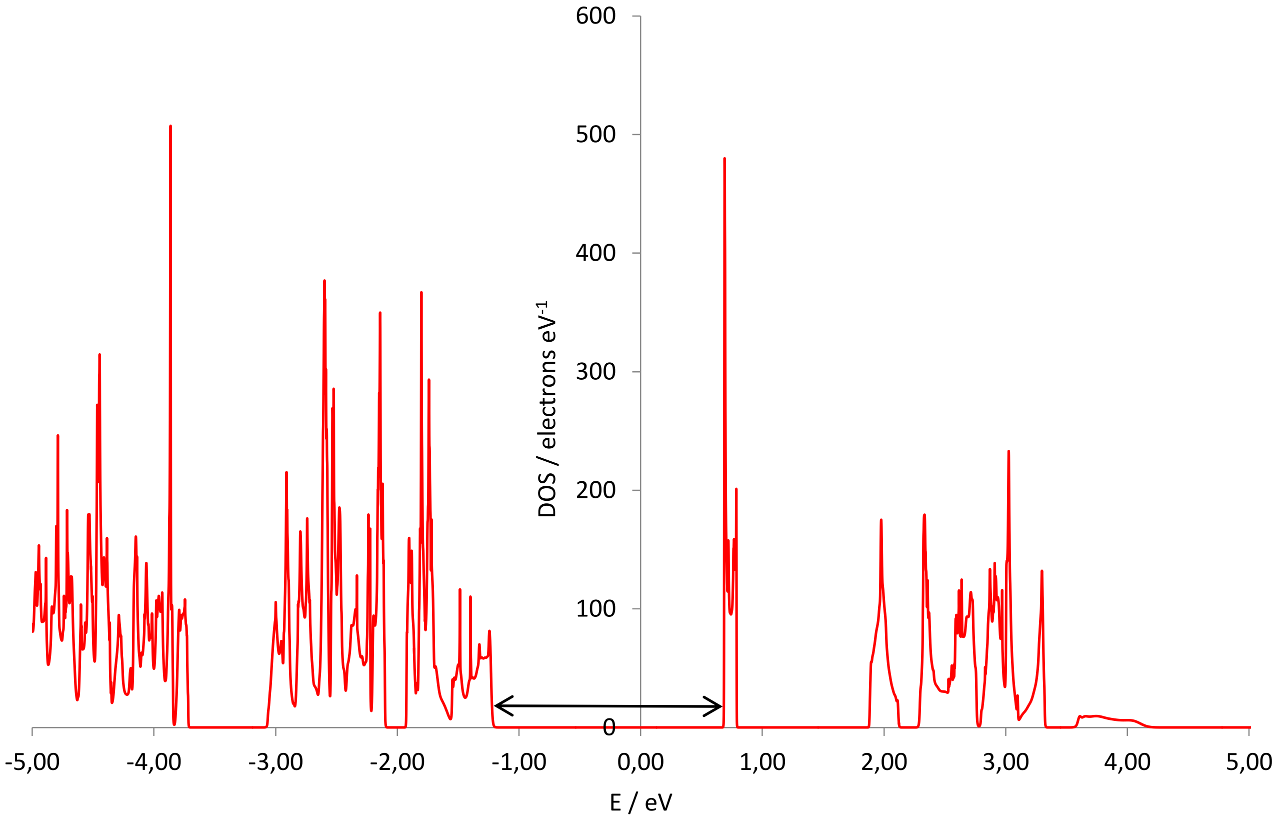

Figure S52. The DOS plot for flsZ (yellow form). The arrow shows the position of the band gap.

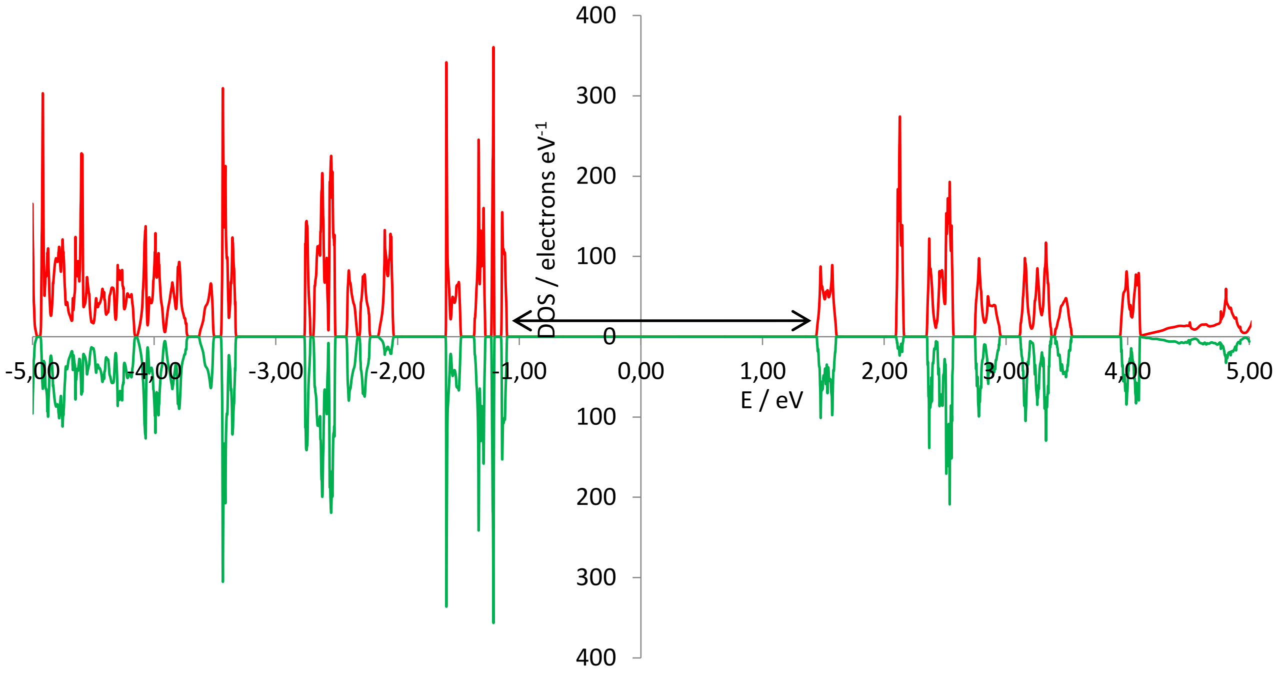

Figure S53. The DOS (top, red) and PDOS (bottom, green) plot for flsL:acetone monosolvate form I. From the comparison of the two plots it is evident that acetone does not contribute significantly to the frontier bands and therefore does not affect the value of the band gap.

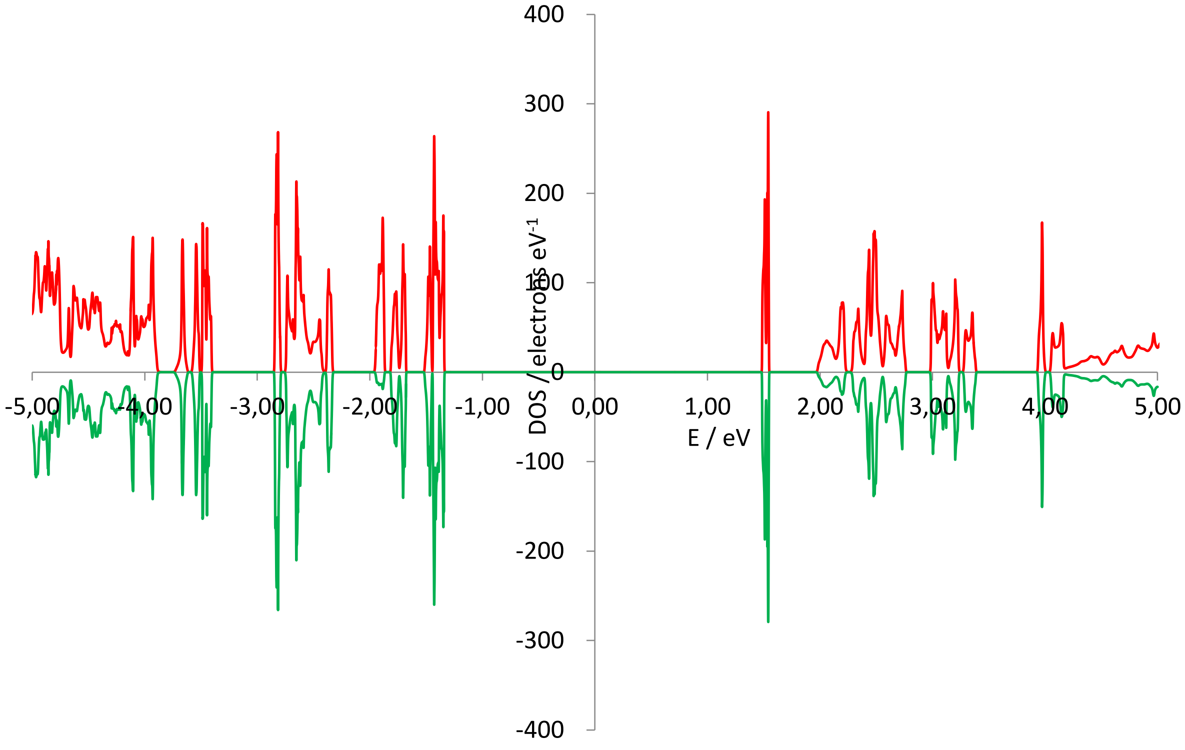

Figure S54. The DOS (top, red) and PDOS (bottom, green) plot for flsL:acetone monosolvate form II. From the comparison of the two plots it is evident that acetone does not contribute significantly to the frontier bands and therefore does not affect the value of the band gap.

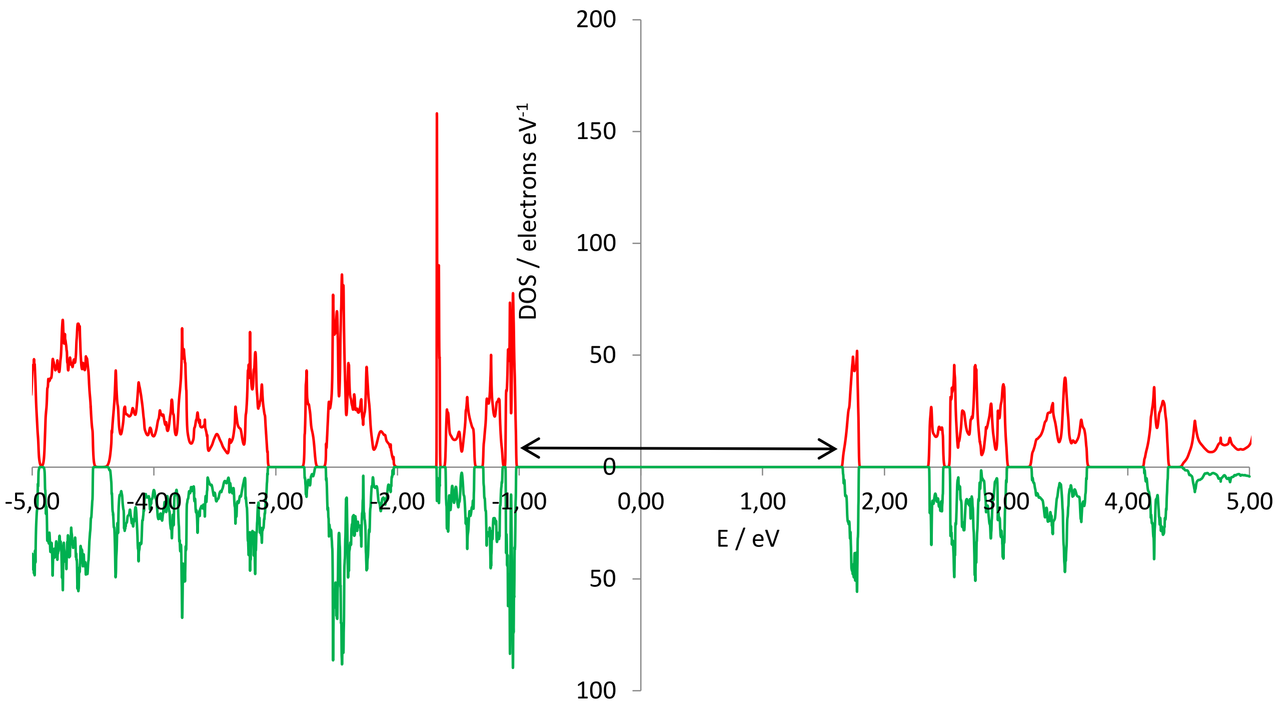

Figure S55. The DOS (top, red) and PDOS (bottom, green) plot for flsL:dioxane hemisolvate. From the comparison of the two plots it is evident that dioxane does not contribute significantly to the frontier bands and therefore does not affect the value of the band gap.

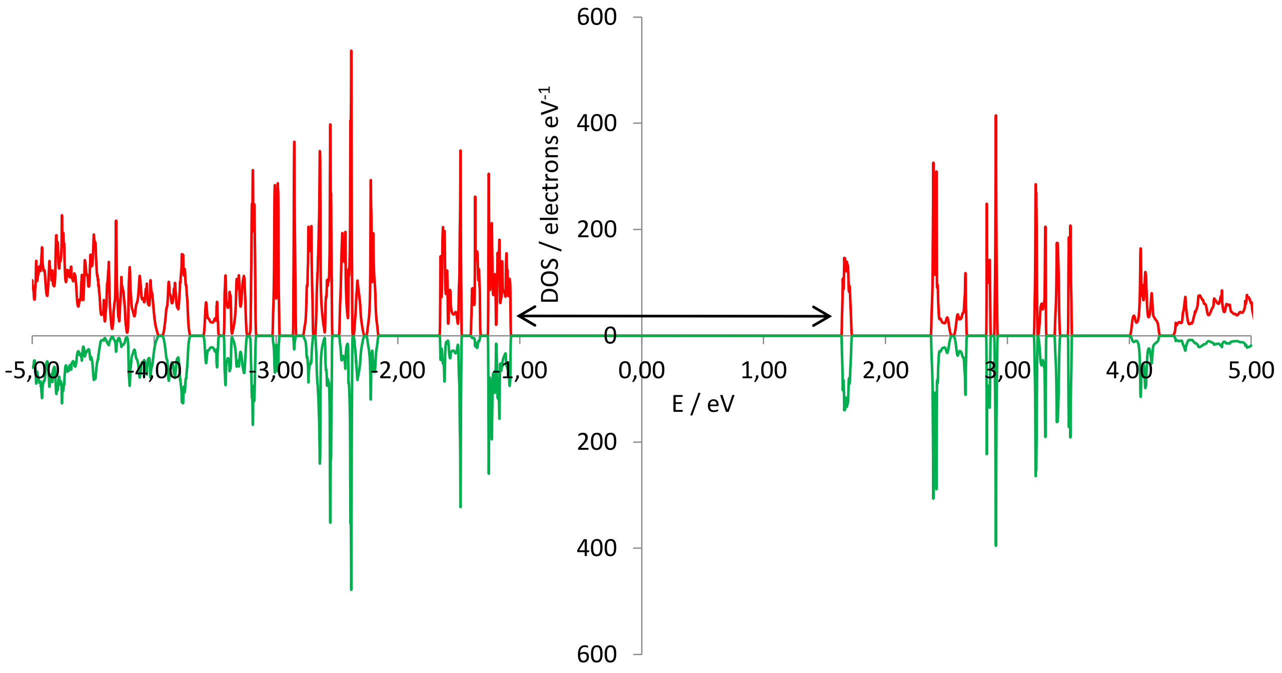

Figure S56. The DOS (top, red) and PDOS (bottom, green) plot for flsL:dioxane hemipentasolvate. From the comparison of the two plots it is evident that dioxane does not contribute significantly to the frontier bands and therefore does not affect the value of the band gap.

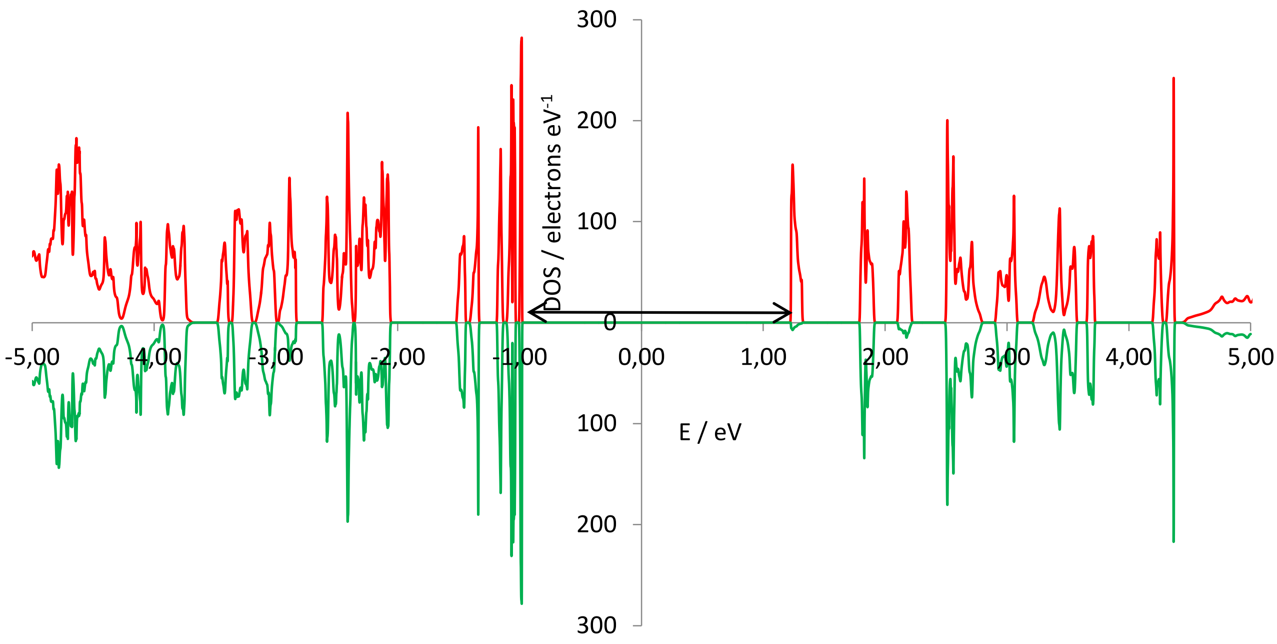

Figure S57. The DOS (top, red) and PDOS (bottom, green) plot for the flsL:pyrazine cocrystal. From the comparison of the two plots it is evident that the lowest unoccupied band at +1.24 eV is almost entirely localized on pyrazine molecules. The presence of pyrazine in the crystal structure therefore lowers the band gap of the material.

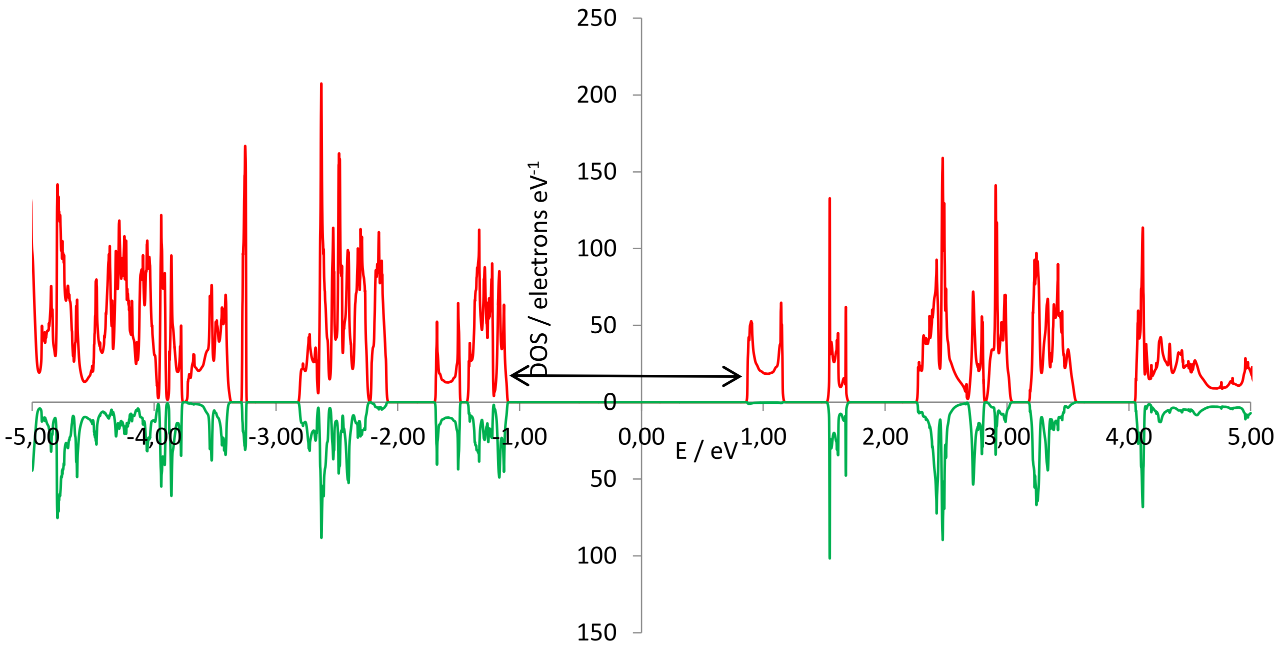

Figure S58. The DOS (top, red) and PDOS (bottom, green) plot for the flsL:acridine cocrystal. From the comparison of the two plots it is evident that the lowest unoccupied band at +0.85 eV is almost entirely localized on acridine molecules. The presence of acridine in the crystal structure therefore lowers the band gap of the material.

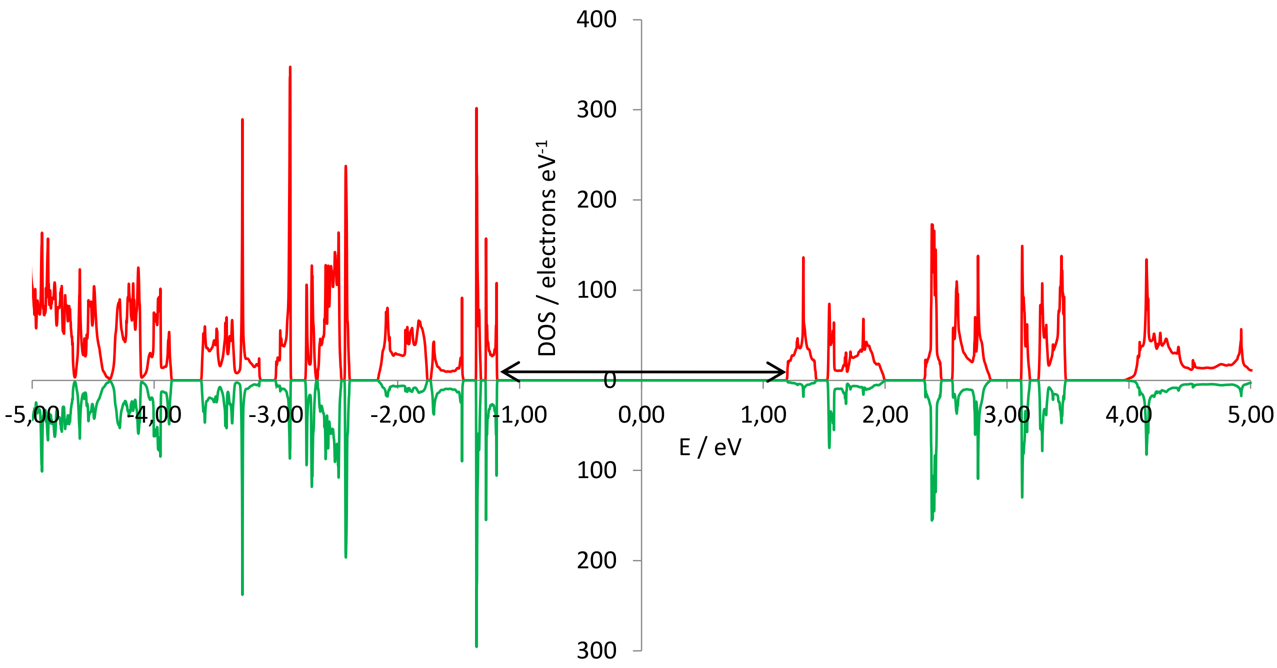

Figure S59. The DOS (top, red) and PDOS (bottom, green) plot for the flsL:phenanthridine cocrystal. From the comparison of the two plots it is evident that the lowest unoccupied band at +1.19 eV is almost entirely localized on phenanthridine molecules. The presence of phenanthridine in the crystal structure therefore lowers the band gap of the material.

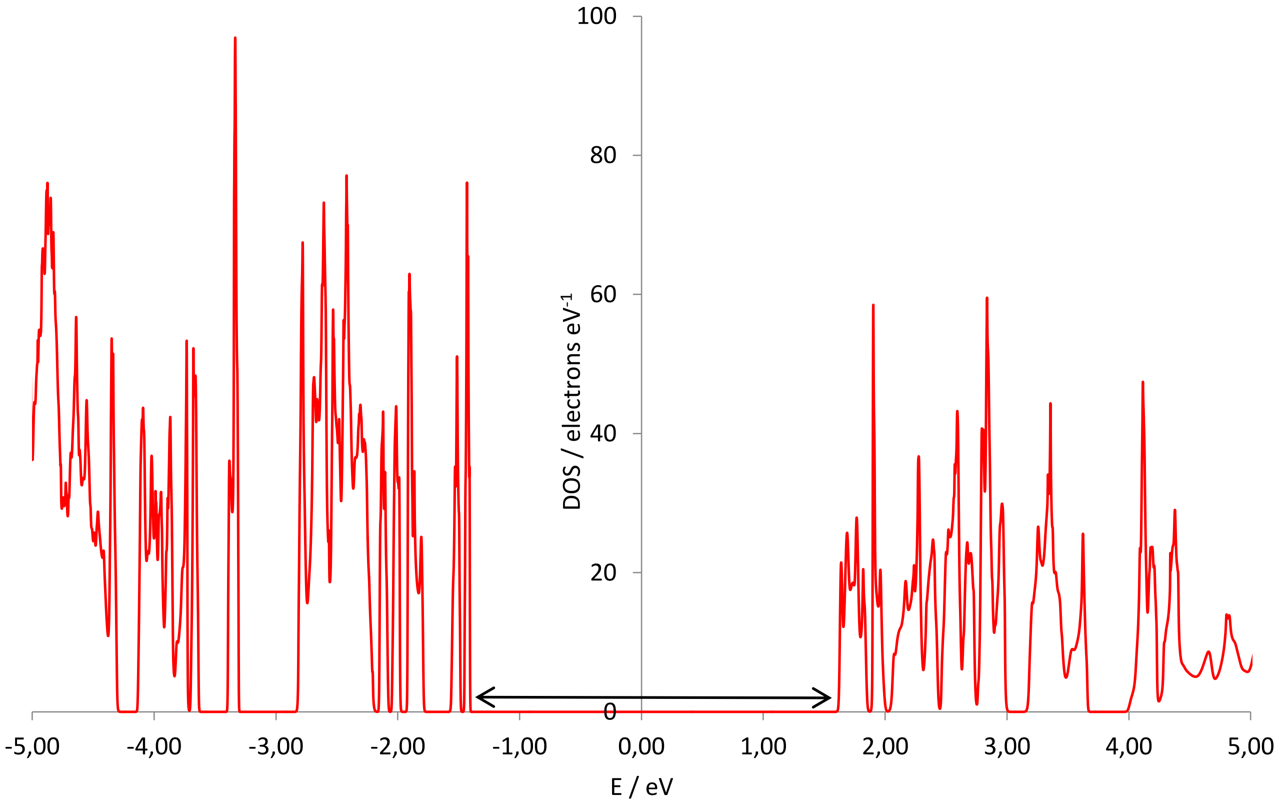

Figure S60. The DOS plot for the lactoid form of diacetylfluorescein.

Table S9. Comparison of the measured and calculated band gaps of different fluorescein crystal forms.

Material	Measured band gap / eV	Calculated band gap / eV	
		full DOS	fluorescein PDOS
flsQ (red form)	1.86	1.75	-
flsZ (yellow form)	2.30	1.94	-
flsL:acetone monosolvate	3.56	2.49	2.49
flsL:dioxane hemisolvate	3.96	2.69	2.69
flsL:dioxane hemipentasolvate	3.91	2.72	2.72
flsL:pyrazine cocrystal	3.60	2.23	2.78
flsL:acridine cocrystal	2.92	1.98	2.64
flsL:phenanthridine cocrystal	3.39	2.40	2.74
diacetylfluorescein	3.97	3.04	-

9. References

[1] R. S. Osborn, D. Rogers, *Acta Crystallogr. Sect. B Struct. Crystallogr. Cryst. Chem.* **1975**, *31*, 359–364.

[2] D.-K. Bučar, S. Filip, M. Arhangelskis, G. O. Lloyd, W. Jones, *CrystEngComm* **2013**, *15*, 6289–6291.

[3] A. Boultif, D. Louër, *J. Appl. Crystallogr.* **2004**, *37*, 724–731.

[4] W. I. F. David, K. Shankland, J. van de Streek, E. Pidcock, W. D. S. Motherwell, J. C. Cole, *J. Appl. Crystallogr.* **2006**, *39*, 910–915.

[5] A. Coelho, **2007**.

[6] G. S. Pawley, *J. Appl. Crystallogr.* **1981**, *14*, 357–361.

[7] A. L. Spek, *Acta Crystallogr. D. Biol. Crystallogr.* **2009**, *65*, 148–55.

[8] H. M. Rietveld, *Acta Crystallogr.* **1967**, *22*, 151–152.

[9] M. Järvinen, *J. Appl. Crystallogr.* **1993**, *26*, 525–531.

[10] Agilent Technologies, **2013**.

[11] G. M. Sheldrick, *Acta Crystallogr. A.* **2008**, *64*, 112–22.

[12] M. D. Eddleston, K. E. Hejczyk, E. G. Bithell, G. M. Day, W. Jones, *Chemistry* **2013**, *19*, 7883–8.

[13] M. D. Eddleston, K. E. Hejczyk, E. G. Bithell, G. M. Day, W. Jones, *Chem. Eur. J.* **2013**, *19*, 7874–82.

[14] Advanced Chemistry Development, Inc.: Toronto, **2014**.

[15] A. D. Becke, *J. Chem. Phys.* **1993**, *98*, 5648–5651.

[16] M. J. Frisch, G. W. Trucks, H. B. Schlegel, G. E. Scuseria, M. A. Robb, J. R. Cheeseman, J. A. Montgomery, T. Vreven, K. N. Kudin, J. C. Burant, J. M. Millam, S. S. Iyengar, J. Tomasi, V. Barone, B. Mennucci, M. Cossi, G. Scalmani, N. Rega, G. A. Petersson, H. Nakatsuji, M. Hada, M. Ehara, K. Toyota, R. Fukuda, J. Hasegawa, M. Ishida, T. Nakajima, Y. Honda, O. Kitao, H. Nakai, M. Klene, X. Li, J. E. Knox, H. P. Hratchian, J. B. Cross, V. Bakken, C. Adamo, J. Jaramillo, R. Gomperts, R. E. Stratmann, O. Yazyev, A. J. Austin, R. Cammi, C. Pomelli, J. W. Ochterski, P. Y. Ayala, K. Morokuma, G. A. Voth, P. Salvador, J. J. Dannenberg, V. G. Zakrzewski, S. Dapprich, A. D. Daniels, M. C. Strain, O. Farkas, D. K. Malick, A. D. Rabuck, K. Raghavachari, J. B. Foresman, J. V Ortiz, Q. Cui, A. G. Baboul, S. Clifford, J. Cioslowski, B. B. Stefanov, G. Liu, A. Liashenko, P. Piskorz, I. Komaromi, R. L. Martin, D. J. Fox, T. Keith, A. M. A. Laham, C. Y. Peng, A. Nanayakkara, M. Challacombe, P. M. W. Gill, B. Johnson, W. Chen, M. W. Wong, C. Gonzalez, J. A. Pople, **2003**.

[17] P. G. Karamertzanis, C. C. Pantelides, *J. Comput. Chem.* **2005**, *26*, 304–24.

[18] D. E. Williams, *J. Mol. Struct.* **1999**, *485-486*, 321–347.

[19] D. E. Williams, *J. Comput. Chem.* **2001**, *22*, 1154–1166.

[20] D. E. Williams, *J. Comput. Chem.* **2001**, *22*, 1–20.

[21] C. M. Breneman, K. B. Wiberg, *J. Comput. Chem.* **1990**, *11*, 361–373.

[22] A. J. Stone, M. Alderton, *Mol. Phys.* **1985**, *56*, 1047–1064.

[23] C. H. Görbitz, B. Dalhus, G. M. Day, *Phys. Chem. Chem. Phys.* **2010**, *12*, 8466–77.

[24] A. V. Kazantsev, P. G. Karamertzanis, C. S. Adjiman, C. C. Pantelides, *J. Chem. Theory Comput.* **2011**, *7*, 1998–2016.

[25] M. Cossi, V. Barone, B. Mennucci, J. Tomasi, *Chem. Phys. Lett.* **1998**, *286*, 253–260.

[26] B. Mennucci, J. Tomasi, *J. Chem. Phys.* **1997**, *106*, 5151–5158.

[27] M. Cossi, G. Scalmani, N. Rega, V. Barone, *J. Chem. Phys.* **2002**, *117*, 43.

[28] T. G. Cooper, K. E. Hejczyk, W. Jones, G. M. Day, *J. Chem. Theory Comput.* **2008**, *4*, 1795–1805.

[29] S. J. Clark, M. D. Segall, C. J. Pickard, P. J. Hasnip, M. I. J. Probert, K. Refson, M. C. Payne, *Zeitschrift für Krist.* **2005**, *220*, 567–570.

[30] J. P. Perdew, K. Burke, M. Ernzerhof, *Phys. Rev. Lett.* **1996**, *77*, 3865–3868.

[31] A. Rappe, K. Rabe, E. Kaxiras, J. Joannopoulos, *Phys. Rev. B* **1990**, *41*, 1227–1230.

[32] H. J. Monkhorst, J. D. Pack, *Phys. Rev. B* **1976**, *13*, 5188–5192.

[33] S. Grimme, *J. Comput. Chem.* **2006**, *27*, 1787–99.

[34] E. R. McNellis, J. Meyer, K. Reuter, *Phys. Rev. B* **2009**, *80*, 205414.

[35] A. J. Morris, R. J. Nicholls, C. J. Pickard, J. R. Yates, *Comput. Phys. Commun.* **2014**, *185*, 1477–1485.

[36] R. J. Nicholls, A. J. Morris, C. J. Pickard, J. R. Yates, *J. Phys. Conf. Ser.* **2012**, *371*, 012062.

[37] J. R. Yates, X. Wang, D. Vanderbilt, I. Souza, *Phys. Rev. B* **2007**, *75*, 195121.

[38] C. J. Pickard, F. Mauri, *Phys. Rev. B* **2001**, *63*, 245101.

[39] J. R. Yates, C. J. Pickard, F. Mauri, *Phys. Rev. B* **2007**, *76*, 024401.

[40] D. Presti, A. Pedone, M. C. Menziani, *Inorg. Chem.* **2014**, 53, 7926–35.
